# A latent class approach for sepsis diagnosis supports use of procalcitonin in the emergency room for diagnosis of severe sepsis

**DOI:** 10.1186/1471-2253-13-23

**Published:** 2013-09-19

**Authors:** Fabián A Jaimes, Gisela D De La Rosa, Marta L Valencia, Clara M Arango, Carlos I Gomez, Alex Garcia, Sigifredo Ospina, Susana C Osorno, Adriana I Henao

**Affiliations:** 1 Department of Internal Medicine, School of Medicine, Universidad de Antioquia, Medellín AA 1226, Colombia; 2 Department of Critical Care, Hospital Pablo Tobón Uribe, Medellín, Colombia; 3 Department of Internal Medicine, Hospital Pablo Tobón Uribe, Medellin, Colombia; 4 Intensive Care Unit, Clínica Universitaria Bolivariana, Medellín, Colombia; 5 Department of Epidemiology, Hospital Universitario San Vicente de Paul, Medellín, Colombia; 6 Research Unit, Hospital Pablo Tobón Uribe, Medellín, Colombia; 7 Grupo Académico de Epidemiología Clínica (GRAEPIC), Universidad de Antioquia, Medellín, Colombia

**Keywords:** Sensitivity, Specificity, Sepsis, Latent class, C-reactive protein, Procalcitonin, D-dimer

## Abstract

**Background:**

Given the acknowledged problems in sepsis diagnosis, we use a novel way with the application of the latent class analysis (LCA) to determine the operative characteristics of C-reactive protein (CRP), D-dimer (DD) and Procalcitonin (PCT) as diagnostic tests for sepsis in patients admitted to hospital care with a presumptive infection.

**Methods:**

Cross-sectional study to determine the diagnostic accuracy of three biological markers against the gold standard of clinical definition of sepsis provided by an expert committee, and also against the likelihood of sepsis according to LCA. Patients were recruited in the emergency room within 24 hours of hospitalization and were follow-up daily until discharge.

**Results:**

Among 765 patients, the expert committee classified 505 patients (66%) with sepsis, 112 (15%) with infection but without sepsis and 148 (19%) without infection. The best cut-offs points for CRP, DD, and PCT were 7.8 mg/dl, 1616 ng/ml and 0.3 ng/ml, respectively; but, neither sensitivity nor specificity reach 70% for any biomarker. The LCA analysis with the same three tests identified a “cluster” of 187 patients with several characteristics suggesting a more severe condition as well as better microbiological confirmation. Assuming this subset of patients as the new prevalence of sepsis, the ROC curve analysis identified new cut-off points for the tests and suggesting a better discriminatory ability for PCT with a value of 2 ng/ml.

**Conclusions:**

Under a “classical” definition of sepsis three typical biomarkers (CRP, PCT and DD) are not capable enough to differentiate septic from non-septic patients in the ER. However, a higher level of PCT discriminates a selected group of patients with severe sepsis.

## Background

Sepsis is defined as the host response to infection and it is an important cause of morbidity and mortality around the world [1,2]. The surviving sepsis campaign issued a call for global action against sepsis and pointed out diagnosis as a fundamental challenge [3,4]. In early stages of the process, the source of infection may be unclear and the related systemic response indistinguishable of no-infectious diseases. Consequently, clinicians often miss or delay this diagnosis. This is especially worrying; since there is strong evidence supporting that early treatment is associated with greater clinical success [5]. An ideal “Gold Standard” is not available for sepsis diagnosis, as microbiology is not enough sensitive and laboratory tests are not specific for using as reference standards. The lack of any reference standard has been overcome by using techniques that avoid the need for comparison with a single accurate test. These techniques can be broadly divided into latent class analysis (LCA) and Bayesian analysis [6]. LCA has been used widely in psychiatry as well as other disciplines [7-11] but, it has not been yet applied to the evaluation of sepsis.

On the other hand, sepsis is associated with the simultaneous activation of the inflammatory and coagulation cascades, and most of their components are markers or mediators in the host response [12,13]. From this close interplay between inflammation and coagulation, which is a recognized way toward organ dysfunction and mortality [14], emerges the rationale to characterize the host response to infection. Three potential biomarkers have shown regular presence in systemic infections: C-reactive protein (CRP), procalcitonin (PCT), and D-dimer (DD); the latter as an unspecific signal of coagulation activation [15-19]. So far, however, no large prospective studies support any of them as a single independent criterion for sepsis. We aimed to estimate the diagnostic accuracy of these three biomarkers as diagnostic tests for sepsis, with the application of the latent class analysis, in patients at the ER admittance with a presumptive infection as main diagnosis.

## Methods

Prospective single center study on the diagnostic accuracy of a test. The study protocol and a pre-specified nested analysis were previously published [20,21].

### Setting

Emergency Room (ER) at the “Hospital Universitario San Vicente de Paúl” (Medellín, Colombia). This is a 550-bed, fourth level University Hospital with an admission rate of approximately 1800 patients per month through the ER and is a reference institution for a region including approximately 3 million habitants.

### Subjects

Inclusion criteria: 1. Patients hospitalized in the ER within 24 hours before admission to the study. 2. Aged 18 years or older. 3. At least one of the following causes as the main admission diagnosis to the hospital: a) any kind of infectious disease (confirmed or suspected), b) fever of unknown origin, c) delirium or any kind of encephalopathy of unknown origin or d) acute hypotension not explained by hemorrhage, myocardial infarction, stroke or heart failure. We selected these relatively broad criteria according with the last consensus conference on sepsis definitions [22].

Exclusion criteria: 1. Negative of the patients, their families, or the attending physician to be part of the study. 2. Antimicrobial treatment received at another medical institution immediately before admission to the study. 3. Medical decision to treat the patient ambulatory or in a different institution within 24 hours after admission. 4. Homeless or inability of the patient to follow up. 5. Previous participation in the same study.

### Recruitment and data collection

We obtained approval for the study from the ethics committee of the Medical Research Centre (University of Antioquia) and the recruited patients provided informed consent. Three physicians (FJ, GDLR, or MLV) and two trained nurses recruited patients by checking admission lists and clinical records and collected data daily from Monday to Saturday of each week. The general protocol for each patient was [20]: collection of baseline clinical data, calculation of entrance Sepsis-related Organ Failure Assessment (SOFA) score [23] and Acute Physiology and Chronic Health Evaluation (APACHE II) score [24] and blood sampling, all of these procedures performed within the first 24 hours of ER admission. During the first 7 days of hospital stay, additionally, the patients were monitored with daily recording of any relevant data registered in medical or nurse records, using a standardized case report form.

### Study tests

CRP, PCT and DD were measured in all patients twice: at admission to the study and on the next day morning (i.e., within 24 hours after the first sample). Serum samples for PCT and CRP were collected in a dry tube with gel separator and centrifuged within the first 2 hours. PCT concentrations were measured by an immunoluminometric assay (VIDAS® B•R•A•H•M•S PCT, Biomeriux, France). CRP was measured quantitatively by an immunoturbidimetric assay using an ARCHITECT® *c*-System (Abbott Laboratories®, USA). Samples for DD were collected in a tube containing citrate as anticoagulant and processed within 2 hours. DD (ng/ml) was measured by a turbidimetric immunoassay in an ACL Elite® coagulometer using a Hemosil™ kit (Instrumentation Laboratory, MA, USA). All previous assays were conducted at the hospital laboratory by trained personnel, under the institution technical standards, and who had no knowledge of the clinical status of the patients, nor the study objectives.

### Gold standards

Clinical gold standard: we used an expert consensus based on clinical, microbiologic, laboratory, and radiologic data collected for each patient during the first 7 days of hospitalization. The experts also took into account the definitions stated in 2001 at the International Sepsis Definitions Conference [22] as well as the Centers for Disease Control and Prevention (CDC) definitions for infection [25]. The consensus was formed by a panel of three physicians with certified training and expertise in intensive care (AG), internal medicine (CMA), and infectious diseases (CIG). First, each physician established a diagnosis individually, in which they agreed on 65% of the cases. The remaining 35% of the patients were fully discussed to determine a final diagnosis. All the experts were blinded to the results of CRP, PCT and DD. The consensus classified the admitted patients into no infected, infected without sepsis and sepsis groups.

Likelihood of sepsis in the study population according to a LCA: this analysis postulates the existence of an unobserved categorical variable that divides the population of interest into classes. Members of the population with a set of observed variables will respond differently depending on the latent class (variable) to which they belong. The problem that the outcome of interest cannot be measured directly occurs in many research situations. Examples include constructs such as intelligence, personality traits or, as in our case, the true sepsis diagnosis. These unobservable outcomes, named also latent variables, can only be measured indirectly by eliciting responses that are related to the construct of interest. These measurable responses are called indicators or manifest variables. Latent variable models are a group of methods that use the information from the manifest variables to identify subtypes of cases defined by the latent variable. The classification appears by modeling the relationship between manifest (CRP, PCT and DD) and latent (sepsis/ no sepsis) variables in such a way that the parameters of interest (prevalence, sensitivity, specificity) are estimable from the implied relations between observable variables. In other words, LCA is just a mathematical model that identifies a subtype or a cluster of observations according to certain defined characteristics or variables that are common to those observations. In this case, we know that different expressions of inflammation and coagulation are common responses in the process of infection. Therefore, we provided these observed variables (DD, PCT and CRP) from all the study population to the model and it is able to uncover the hidden group, i.e. the latent variable, to which the patients belong. In summary, the goal of latent class analysis is to use the observed probabilities to estimate the unobserved ones.

### Sample size and analysis plan

The number of the patients with the disease (ND) that is needed to give a sensitivity of 95%, with a 95% CI +/− 3%, is calculated with the following formula [26]:

ND=Z2α2×sensitivity×1‒sensitivity0,03×22

$$\mathrm{ND}=\frac{1,96^{2}\times 0,95\times \left(0,05\right)}{{\left(0,06\right)}^{2}}=203$$

The ND is also determined by the prevalence (P) of the disease. Hence, the total of patients (TP) required is:

$$\mathrm{TP}=\frac{\mathrm{ND}}{P}$$

We expected a prevalence of sepsis of 30% [2,21], and the sample size would be 700 participants.

Clinical gold standard: the cut points for the study tests were determined using receiver operative characteristics (ROC) curves [27], searching for the best sensitivity and specificity. The method based in the Bayes’ theorem was used to determine the operating characteristics of the tests. Additional analyses were done using changes of the values in the first 24 hours (Δ24) for each test and combining pairs of tests (PCT/DD and CRP/DD). For a Δ24 test, it was considered positive in a patient if her values remain without changes or increase. For combining pair of tests, it was considered positive if both biomarkers were above the cut point. Furthermore, as a sensitivity analysis, two alternative reference standards for sepsis patients were considered: only those who had any microbiologically confirmed infection and only those who were diagnosed as sepsis patients independently by one of the experts (65% among the total population). Patients with missing values were excluded for the corresponding analysis, and results are shown with exact 95% CI using STATA SE (Version 10, Stata Corp, College Station, TX).

LCA: it assumes that results from the three tests in the same subject are independent within the real condition of illness [7]. In other words, if the effect to belong to a latent condition of sepsis would be removed, the effects to the CRP, PCT and DD would have a completely random distribution in the study population. Since that both PCT and CRP values are probably a common expression of the same inflammatory process, we controlled this local independence assumption introducing a random effect through a continuous latent variable [20]. The maximum likelihood estimators of prevalence (the “cluster” of sepsis patients), as well as of sensitivity and specificity of each test if requested, are obtained with an integral that uses an EM iteration algorithm. Analyses were carried out with LATENTGOLD 4.0 (Statistical Innovations, Belmont, MA, USA).

## Results

**Figure 1:**
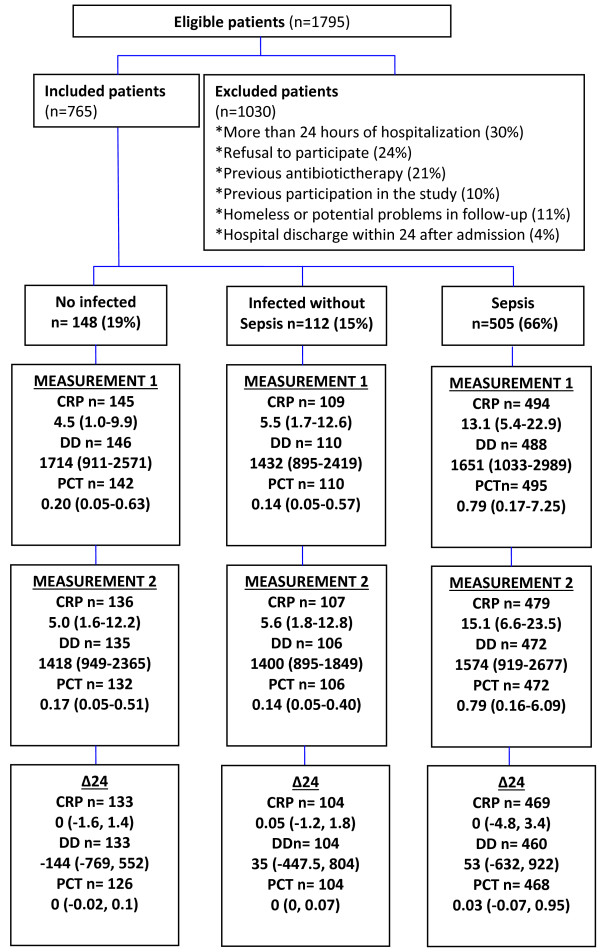
**Flow chart of recruitment, patients’ classification by expert consensus and biomarker results.** The values of biomarkers are presented as number of samples measured, median and interquartile range (IQR). CRP = C-reactive protein (mg/dL), DD = D-dimer (ng/ml), PCT = Procalcitonin (ng/ml), Δ24 = Measurement 2 - Measurement 1.

Enrollment began on August 2007 and concluded on February 2009. A total of 1,795 patients were eligible and 1,030 were excluded, most of them because of more than 24 hours of hospitalization before recruitment and refusal to participate (Figure 1). Among 765 patients included, 683 (89%) had a suspected infection as admission diagnosis, 56 (7%) fever of unknown cause, 20 (3%) delirium or encephalopathy of unknown origin, and 6 (1%) unexplained hypotension. There were 377 males (49%), the mean age was 51.4 years (SD = 20), and the median time of symptoms before consultation was 72 hours (IQR = 24 to192 hours). There was no comorbidity in 307 (40%) of the participants, and the most frequent previous diseases were diabetes mellitus (n *=* 146, 19%), chronic obstructive pulmonary disease (n *=* 94, 12%), chronic renal failure (n *=* 88, 11%), use of corticosteroids or chemotherapy during the past 3 months (n *=* 70, 9%) and trauma or surgery in the previous month (n = 53, 7%). As suspected sources of infection, the most frequent were respiratory (n = 179, 23%) and skin and soft tissues (n = 174, 23%), followed by urinary tract (n = 127, 17%), intrabdominal (n = 93, 12%), undetermined (n = 94, 12%) and others (n = 96, 13%). The median SOFA and APACHE II score were 2 (IQR = 1-4) and 9 (IQR = 5-14), respectively, hospital length of stay was 9 days (IQR = 5 to 17 days), ICU admission was required in 66 patients (9%) and the overall 28-day mortality rate was 12% (n = 91). Due to logistic or technical reasons CRP, DD and PCT at admission were measured in748 (98%), 744 (97%) and 747 (98%) patients, respectively. The median and IQR values for these test were CRP = 9.4 mg/dl (3.5-20), DD = 1673 ng/ml (982–1841) and PCT = 0.4 ng/ml (0.1-3.65).

According to the expert committee 505 patients (66%) were classified with sepsis, 112 (15%) with infection but without sepsis and 148 (19%) without infection. The kappa-statistic measure for multi-rater agreement between experts was 0.65 for sepsis-no sepsis and 0.73 for infection with and without sepsis. Figure 1 shows this classification and their respective biomarker values, and Table 1 shows the main characteristics by group. Among infected patients (with and without sepsis, n = 617) a microbiologic diagnosis was confirmed in 104 patients (17%) by blood culture, in 135 (22%) by urinary culture and in 145 (26%) by other samples. Microorganisms isolated in blood were *E. coli* (n = 29, 28%), *S. aureus* (n = 17, 16%), coagulase-negative staphylococci (n = 15, 14%) and others (n = 43, 42%); and in urine were *E. coli* (n = 79, 59%), *K. pneumoniae* (n = 22, 16%) and others (n = 34, 25%). The main admission diagnosis in the sepsis group (n = 505) was community-acquired pneumonia (n = 115, 23%), followed by urinary tract infection (n = 87, 17%) and soft tissue infection (n = 79, 16%).The main alternative diagnosis in 148 patients without infection were cancer (n = 22,15%), chronic obstructive pulmonary disease (n = 15, 10%), acute pulmonary edema (n = 13, 9%), metabolic diseases (n = 13, 9%), biliary diseases (n = 10, 7%), and others (n = 75, 50%).

**Table 1:** Clinical characteristics at admission according to the groups defined by the expert consensus

**Clinical**	**Sepsis**	**Infected without sepsis**	**No infected**	P* value
**characteristics**	**n = 505**	**n = 112**	**n = 148**	
SOFA score	3 (2–4, 505)	1.5 (1–2, 112)	2 (1–4, 148)	0.001
APACHE II score	10 (6–16, 505)	6 (2–10, 112)	9 (5–14, 148)	0.001
Temperature (°C)	37 (36.5 - 38, 472)	36.9 (36.5 - 37, 102)	37 (36.5 - 37, 131)	0.064
Heart rate	100 (87–115, 493)	83.5 (74–90, 106)	90 (79–108, 146)	0.001
Respiratory rate	22 (20–28, 121)	18 (16–20, 15)	24 (20–36, 27)	0.001
MAP	104 (91–120, 493)	113 (103–126, 106)	108 (95–130, 146)	0.001
WBC (cells/mm^3^)	12900 (8900–17900, 500)	9450 (7500–11400, 112)	10400 (8000–13100, 147)	0.001
Neutrophils (%)	82 (74–89, 500)	71 (60–81, 111)	77 (66–86, 147)	0.001
Hemoglobin (g/dl)	12 (11–14, 500)	12 (11–14, 111)	13 (11–15, 147)	0.069
Creatinin (mg/dl)	1 (0.8 – 1.9, 500)	0.9 (0.8 – 1.3, 110)	0.9 (0.8 – 1.5, 146)	0.035
Lactic Acid (mmol/L)	1.9 (1.2 – 2.9, 494)	1.4 (1.0 – 1.8, 106)	1.7 (1.1 – 2.6, 143)	0.001
Bilirrubin (mg/dl)	0.7 (0.5 – 1.1, 494)	0.6 (0.4 – 0.8, 110)	0.7 (0.4 – 1.1, 142)	0.059
PaO_2_/FiO_2_	304.5 (212–364, 492)	366 (315–407, 104)	307.5 (238 – 387, 142)	0.001
Suspected source of infection				0.001
Respiratory	120 (24%)	16 (14%)	43 (29%)	
Urinary tract	93 (18%)	21 (19%)	13 (9%)	
Skin and soft tissues	115 (23%)	46 (41%)	15 (10%)	
Intra-abdominal	54 (11%)	15 (13%)	24 (16%)	
Undetermined	66 (13%)	3 (3%)	25 (17%)	
Others	57 (11%)	11 (10%)	28 (19%)	
No comorbidity	220 (43%)	55 (49%)	63 (42%)	0.513
Diabetes	98 (19%)	21 (19%)	27 (18%)	0.947
COPD	63 (12%)	4 (3%)	27 (18%)	0.002
CRF	54 (11%)	17 (15%)	17 (11%)	0.404
28-day mortality rate	68 (13.5%)	4 (3.6%)	19 (13%)	0.012

The values are expressed as median (*IQR*, Observations available) or number (percentage). *MAP*, Mean Arterial Pressure, *WBC*, White Blood Cells, *COPD*, Chronic obstructive pulmonary disease, *CRF*, Chronic renal failure.

*Kruskal-Wallis for continuous variables and Chi-square for proportions.

According to the ROC curve analysis, the cut-offs points with the best sensitivity and specificity for CRP, DD, and PCT to discriminate at admission between sepsis and not sepsis (infection without sepsis or not infection) were 7.8 mg/dl, 1616 ng/ml, and 0.3 ng/ml, respectively. Their operating characteristics, at both measurement times, are shown in Table 2. Analyses combining pairs of tests or using changes in the first 24 hours (Δ24) did not show any improvement in diagnostic accuracy. Similar results were seen using the alternative reference standards (data no shown).

**Table 2:** Diagnostic accuracy of CRP, DD and PCT for sepsis diagnosis at admission in the ER according to expert consensus

	**Measurement 1**			**Measurement 2**		
**Operating characteristics**	**CRP**	**DD**	**PCT**	**CRP**	**DD**	**PCT**
	**(7.8 mg/dl)**	**(1616 ng/ml)**	**(0.30 ng/ml)**	**(9.3 mg/dl)**	**(1485 ng/ml)**	**(0.27 ng/ml)**
AUC – ROC	0.71	0.55	0.69	0.72	0.55	0.70
	(0.67 – 0.74)	(0.51 – 0.58)	(0.65 – 0.72)	(0.68 – 0.75)	(0.51 – 0.58)	(0.67 – 0.73)
Sensitivity	66.6%	51.4%	63.8%	68.9%	52.7%	67.2%
	(0.62 – 0.71)	(0.47 – 0.56)	(0.59 – 0.68)	(0.64 – 0.73)	(0.48 – 0.57)	(0.63 – 0.71)
Specificity	66.1%	51.6%	63.9%	68.7%	52.7%	66.4%
	(0.60 – 0.72)	(0.45 – 0.58)	(0.58 – 0.70)	(0.62 – 0.74)	(0.46 – 0.59)	(0.60 – 0.72)
LR +	1.97	1.06	1.77	2.20	1.12	2.00
	(1.64 – 2.36)	(0.91 – 1.24)	(1.48 – 2.11)	(1.81 – 2.68)	(0.95 – 1.31)	(1.65 – 2.41)
LR -	0.50	0.94	0.57	0.45	0.90	0.49
	(0.44 – 0.58)	(0.82 – 1.08)	(0.49 – 0.65)	(0.39 – 0.53)	(0.78 – 1.03)	(0.43 – 0.57)

Values between parentheses are cut-off points and 95% Confidence Interval, respectively. *LR*, Likelihood ratio.

The LCA analysis with the same three tests identified a “cluster” of 187 patients, among those defined as sepsis by the expert committee, with several characteristics suggesting a more severe condition as well as better microbiological confirmation compared to the rest of the study population. According to standard definitions, 70% (n = 131) of these patients had severe sepsis without circulatory failure and 5% (n = 9) had septic shock (Table 3) (Additional file 1). It was not possible to classify 46 patients because of missing values in any of CRP, DD or PCT. Assuming this cluster of 187 patients as the new prevalence of sepsis based on the LCA gold standard, the ROC curve analysis identified new cut-off points for the tests and suggesting a better discriminatory ability for PCT with a value of 2 ng/ml (Table 4).

**Table 3:** Clinical characteristics at admission according to the LCA classification in clusters

**Clinical**	**Cluster 2**	**Cluster 1**	**Missing**	P* value
**characteristics**	**n = 532**	**n = 187**	**n = 46**	
Age	51 (33–68, 532)	54 (37–70, 187)	39 (23–62, 46)	0.067
SOFA score	2 (1–3, 532)	4 (3–6, 187)	2 (1–3, 46)	0.001
APACHE II score	8 (5–13, 532)	13 (9–17,187)	8 (3–11, 46)	0.001
Temperature (°C)	37 (36.5-37.2, 489)	37 (36.7-38, 173)	37 (36.5-38, 43)	0.061
Heart rate	92 (80–108, 520)	100 (88–117, 182)	96 (81–114, 43)	0.001
Respiratory rate	20 (18–28, 90)	24 (20–29, 61)	26.5 (20–38.5, 12)	0.048
MAP	79 (68–90, 495)	72.5 (61–83, 180)	70.5 (60–77, 42)	0.001
WBC (cells/mm^3^)	11200 (8200–15100, 527)	13250 (9300–19300, 186)	13100 (9300–16700, 46)	0.005
Creatinin (mg/dl)	0.9 (0.8-1.3, 529)	1.4 (0.9-2.6, 185)	0.9 (0.7-1.6, 42)	0.001
Platelets (cells/mm^3^)	296000	237000	297000	0.001
	(223000–391000, 525)	(147000–301000, 183)	(186000–404000, 46)	
PaFi	324 (240–384, 515)	292 (190–355, 182)	332 (220–392, 41)	0.001
Lactic Acid (mmo/L)	1.6 (1.1-2.5, 518)	2.0 (1.3-3.1, 184)	1.8 (1.6-2.8, 41)	0.001
Suspected source of infection				0.001
Respiratory	129 (24%)	41 (22%)	9 (19%)	
Urinary tract	76 (14%)	44 (23%)	7 (15%)	
Skin and soft tissues	147 (28%)	20 (11%)	9 (19%)	
Intra-abdominal	60 (11%)	27 (14%)	6 (13%)	
Undetermined	47 (9%)	37 (20%)	10 (22%)	
Others	73 (14%)	18 (10%)	5 (11%)	
No comorbidity	237 (44%)	73 (39%)	28 (61%)	0.027
Diabetes	108 (20%)	33 (18%)	5 (11%)	0.250
COPD	69 (13%)	21 (11%)	4 (9%)	0.614
CRF	54 (10%)	32 (17%)	2 (4%)	0.011
28-day mortality rate	50 (9)	32 (17)	9 (19)	0.005
Blood culture requested	293 (55)	146 (78)	28 (61)	0.001
Positive blood culture	34 (12)	57 (39)	7 (25)	0.001
Procalcitonin (ng/ml)	0.21 (0.05 – 0.64)	15.07 (6.78 – 31.96)	0.64 (0.08 – 3.63)	0.0001
C reactive protein (mg/dl)	7 (2.3 – 15.65)	18.8 (8.3 – 25.9)	13.3 (8.3 – 21.7)	0.0001
D- dimer (ng/ml)	1406 (893 – 2329)	2883 (1386 – 5018)	1700 (1279 – 2398)	0.0001

The values are expressed as median (*IQR*, observations available) or number (percentage).

Missing = patients without information in any of PCT, CRP or DD; Cluster 1 = patients identified by LCA as a sepsis-like syndrome; Cluster 2 = the remaining of the study population; *MAP*, Mean Arterial Pressure; *WBC*, White Blood Cells; *PaFi*, PaO_2_/FiO_2_; *COPD*, Chronic obstructive pulmonary disease; *CRF*, Chronic renal failure.

*Kruskal-Wallis for continuous variables and Chi-square for proportions.

**Table 4:** Diagnostic accuracy of CRP, DD and PCT for sepsis diagnosis according to the LCA gold standard

**Operating characteristics**	**CRP**	**DD**	**PCT**
	**(12 mg/dl)**	**(1848 ng/ml)**	**(2.06 ng/ml)**
AUC - ROC	0.71	0.73	0.95
	(0.68 – 0.74)	(0.69 – 0.76)	(0.93 – 0.96)
Sensitivity	64.17%	65.24%	91.44%
	(0.57 – 0.71)	(0.58 – 0.72)	(0.86 – 0.95)
Specificity	64.47%	65.23%	91.35%
	(0.60 – 0.68)	(0.61 – 0.69)	(0.89 – 0.94)
LR +	1.81	1.88	10.81
	(1.54 – 2.11)	(1.60 – 2.19)	(8.15 – 14.35)
LR -	0.56	0.53	0.09
	(0.45 – 0.68)	(0.43 – 0.66)	(0.06 – 0.15)

Values between parentheses are cut-off points and 95% Confidence Interval, respectively. *LR*, likelihood ratio.

## Discussion

Our results suggest that, under a “classical” definition of sepsis, three typical biomarkers (CRP, PCT and DD) are not capable enough to differentiate septic from non-septic patients in the ER. Indeed, the kappa-statistic measure for multi-rater agreement between experts for this definition was 0.65 for sepsis-no sepsis and 0.73 for infection with and without sepsis, which underlines the limitations for clinical diagnosis in this condition. Using another analytic approach, however, a higher cut-off point for PCT (2 ng/ml) is able to identify and to exclude a specific population more severely ill and with better microbiological confirmation. To the best of our knowledge, this is the first research that incorporates the novel concept of a latent class to the process of diagnosis in sepsis.

The performance of a diagnostic test is judged by how accurately the test result can identify a diseased or no diseased person. The true disease status is the “gold standard” against which a test should be compared. However, there are many conditions for which the definitive diagnosis is very difficult or expensive to establish. This is especially true for the diagnosis of a complex clinical condition as sepsis, in which even within the construct of “systemic response to infection” there is not a real “gold standard” against which the diagnostic criteria can be calibrated [22,28,29]. Psychological and social sciences have a long tradition in coping with primary study objects that are not directly observable. Constructs such as intelligence, fear or trust can only be measured indirectly. Inference proceeds by modeling the relationship between observable and latent variables in such a way that the parameters of interest are estimable from the implied relations between observable variables. When the unobservable variable is categorical, the term latent class analysis (LCA) applies [6,7]. In other words, LCA postulates the existence of an unobserved categorical variable that divides the population of interest in to classes. Members of the population with a set of observed variables will respond differently depending on the latent class to which they belong. This technique can be applied to the problems related to diagnostic testing, with the unobserved categorical variable being “disease present” or “disease absent” [20].

Given the established interplay between inflammation and coagulation in sepsis [14,30-32], it is reasonable to characterize the host response to infection as a potential diagnosis tool on the basis of three recognized markers of these two cascades. The sensible mathematical model of the latent diagnostic classification, using individuals’ values of CRP, DD and PCT, was able to identify a subset of patients attended in the ER with suspicion of infection and with clear differences in clinical status, microbiological profile and 28-day mortality. Although this subset was identified among those patients classified as sepsis by the expert committee, there is not a unique clear cut-off in any variable or test that may define the cluster specifically as severe or bacteremic sepsis (Table 3). Furthermore, among these three potential biomarkers, PCT proved to be the most contributor to the “new” standard of more severe disease but with a higher cut-off point than that usually suggested. Our main result, consequently, is that PCT is useful to identify a subgroup of more severely ill septic patients attending the ER. Such a finding was previously reported by Hausfater P et al. [33], whom studied 243 patients with body temperature of 38.5°C or greater attended in the adult emergency department of an academic tertiary care hospital. They found, using standard statistical methods, that PCT is an independent variable that can predict whether a febrile episode has a bacterial origin, and that at a threshold of 2 μg/l it is independently associated with critical illness. The coincidence with our findings is remarkable, despite the fact that their study population was extremely different: all the patients consulted by a febrile episode, 29% of them were immunocompromised, and only 81% were hospitalized in that consultation.

Needless to say, sepsis is not an illness but a syndrome suspected mainly on clinical criteria, and the misdiagnosis of sepsis is associated with an extremely adverse outcome. Consequently, we are not proposing a new methodological approach for sepsis diagnosis. Instead, we are identifying a new cut-off point for procalcitonin to be able to detect more severely ill patients. This goal was not achieved by the conventional clinical gold standard with expert consensus and, in this way, LCA is just an instrument to show that we can improve the process of sepsis diagnosis in the emergency room. The recent literature is full of studies evaluating PCT for sepsis diagnosis [16,17,34,35], but in the setting of the ER there are less investigations testing its potential usefulness. In a secondary care hospital of Finland, a population of 539 patients admitted to the ER with suspicion of infection and with clinician’s order for blood cultures was studied [16]. In assessing how the parameters differentiated all sepsis patients (n = 358) from patients with no sepsis (n = 181), AUC-ROC for PCT was 0.73 (95% CI 0.69 – 0.78), and PCT emerged as the best marker for severe sepsis with an AUC-ROC = 0.77 (95% CI 0.71 – 0.84). Riedel et al. evaluated the usefulness of PCT as a diagnostic predictive marker of bacteremia and sepsis in 259 patients who had blood cultures obtained in the ER of a tertiary medical center in Baltimore [35]. In16 patients there was evidence of bacteremia and 12(75%) patients had a PCT level of more than 0.1 ng/ml. The PCT cut-off value that maximizes the AUC-ROC (0.79) was 0.1475 ng/ml., but with sensitivity just in 75% and specificity of 79.8%. In a recent meta-analysis [36], Wacker C. et al. analyzed 30 reports, although only two from ER, accounting for 3244 patients. Bivariate analysis yielded a mean sensitivity of 0.77 (95% CI = 0.72–0.81) and specificity of 0.79 (95% CI = 0.74–0.84) and the area under the receiver operating characteristic curve was 0.85 (95% CI = 0.81–0.88). The median cut-off for PCT of the studies included was 1.1 ng/ml (IQR = 0.5–2.0) and the absence of a threshold effect suggests that a cut-off between 1.0 and 2.0 ng/ml, close to our findings, is helpful for discrimination of patients with sepsis from other inflammatory conditions. However, the studies had substantial heterogeneity (*I*^2^ = 96%, 95% CI = 94–99) and none of the subgroups investigated like population, admission category, assay used, severity of disease, and description and masking of the reference standard, could account for that heterogeneity. They concluded that the test may be helpful for diagnosis of sepsis in critically ill patients, but it must be interpreted in context with information from careful medical history, physical examination and microbiological assessment.

Our study has several limitations. First, this is a single center study in a specific geographic location with some particularities from an epidemiological point of view [2,37], which are obstacles for external validity. Moreover, the pre-test probability of sepsis should be significantly different in patients admitted to the ER and in patients admitted to ICU, even with the same clinical suspicion of bacterial infection, and this is an acknowledged consideration in the use and interpretation of any diagnostic test. As we mentioned before, the clinical diagnostic “gold standard” utilized here performed poorly, as the concordance between experts was 0.65 for sepsis-no sepsis and 0.73 for infection with and without sepsis. This weakness, indeed, underlines the limitations for clinical diagnosis in this condition. On the other hand, LCA also has its limitations as “gold” standard. Under this approach, sepsis is not formally defined but rather is a mathematically defined entity that does not necessarily correspond with a clinically relevant status. Additionally, LCA modeling requires sophisticated analytic techniques and software, and the full model or the hypothetical “true” state of disease cannot be fully tested with the observed data. Finally, although blood sampling was performed immediately after the patient was admitted to the study, he/she could be in the ER at any time within the last 24 hours before recruitment. This is important because biomarker’s kinetic, notably PCT, and their levels may varying considerably during 24 hours.

## Conclusions

In summary, the “holy grail” of sepsis diagnosis is an evolving process and the fine exercise of clinical suspicion should be complemented by appropriate laboratory test. In this scenario, PCT emerges as an acceptable choice underscoring both microbiology and prognosis in selected patients. A higher level of PCT seems related more strongly with these two components of the infectious phenomena.

### Key messages

– 1CRP, PCT and DD are not capable enough to differentiate septic from non-septic patients in the ER.

– A higher level of PCT seems related more strongly with microbiology and prognosis, and discriminates a selected group of patients with severe sepsis.

– Determination of the PCT level may be useful for screening and prognosis of more-severely ill ER patients.

## Competing interests

Funded by COLCIENCIAS (Grant 1115-343-19153) and Estrategia de Sostenibilidad 2013–2014 Universidad de Antioquia. The authors declare that they have no competing interests and the investigation was conducted with ethical adherence to investigations in humans.

## Authors’ contributions

FJ and GD conceived and designed the study and obtained research funding. FJ, GD, MV, CA, CG, AG, SO, SO and AH supervised the conduction of the study and data collection. FJ, GD, MV undertook recruitment of participating patients and managed the data, including quality control. FJ provided statistical advice on study design and analyzed the data. FJ chaired the data oversight committee. FJ drafted the manuscript and all authors contributed substantially to its revision and final approval of the version to be published. FJ and GD take responsibility for the paper as a whole. All authors read and approved the final manuscript.

## Pre-publication history

The pre-publication history for this paper can be accessed here:

http://www.biomedcentral.com/1471-2253/13/23/prepub

## Supplementary Material

Additional file 1 Supplementary statistical analysis and results of the LATENT GOLD software. Click here for file
