# Supplementary material for: A latent class approach for sepsis diagnosis supports use of procalcitonin in the emergency room for diagnosis of severe sepsis
Source: BMC Anesthesiol. 2013 Sep 19;13:23. doi: 10.1186/1471-2253-13-23 (PMC3850719; doi:10.1186/1471-2253-13-23)
Supplement: Additional file 1 — Supplementary statistical analysis and results of the LATENT GOLD software. [file 1471-2253-13-23-S1.html]

LatentGold output

#### disepsis\_definitiva\_CL.sav

|  |  |  |  |  |  |
| --- | --- | --- | --- | --- | --- |
| **File name:** | **C:\Users\Fabian Jaimes\Documents\Johana\Disepsis\DISEPSIS\Clases latentes\Clases latentes\_dic16\disepsis\_definitiva\_CL.sav** | | | | |
| **File size:** | 940445 bytes |  |  |  |  |
| **File date:** | 2009-dic-15 | 02:58:30 p.m. |  |  |  |
|  |  | **LL** | **BIC(LL)** | **AIC(LL)** | **Npar** |
| **Model1** | 1-Cluster | -12783.3866 | 25606.2405 | 25578.7733 | 6 |
| **Model2** | 2-Cluster | -11148.3941 | 22382.3004 | 22322.7882 | 13 |
| **Model3** | 2-Cluster 1-CFactor | -11133.0585 | 22371.3627 | 22298.1169 | 16 |
| **Model6** | 3-Cluster |  |  |  |  |

#### Model1 - LL = -12783.3866

|  |  |  |
| --- | --- | --- |
| **1-Cluster Model** | | |
|  |  |  |
| **Number of cases** | 719 |  |
| **Number of parameters (Npar)** | 6 |  |
| **Activated Constraints** | 0 |  |
| **Robustness Effect** | 6.7114 |  |
| **Random Seed** | 507582 |  |
| **Best Start Seed** | 507582 |  |
|  |  |  |
| **Log-likelihood Statistics** |  |  |
| **Log-likelihood (LL)** | -12783.3866 |  |
| **Log-prior** | -15.0226 |  |
| **Log-posterior** | -12798.4092 |  |
| **BIC (based on LL)** | 25606.2405 |  |
| **AIC (based on LL)** | 25578.7733 |  |
| **AIC3 (based on LL)** | 25584.7733 |  |
| **CAIC (based on LL)** | 25612.2405 |  |
|  |  |  |
| **Classification Statistics** | **Clusters** |  |
| **Classification errors** | 0.0000 |  |
| **Reduction of errors (Lambda)** | 1.0000 |  |
| **Entropy R-squared** | 1.0000 |  |
| **Standard R-squared** | 1.0000 |  |
| **Classification log-likelihood** | -12783.3866 |  |
| **AWE** | 25663.7076 |  |
|  |  |  |
| **Classification Table** | **Modal** |  |
| **Probabilistic** | **Cluster1** | **Total** |
| **Cluster1** | 719.0000 | 719.0000 |
| **Total** | 719.0000 | 719.0000 |
|  |  |  |
| **Files** |  |  |
| **Infile** | C:\Users\Fabian Jaimes\Documents\Johana\Disepsis\DISEPSIS\Clases latentes\Clases latentes\_dic16\disepsis\_definitiva\_CL.sav | |
|  |  |  |
| **Variable Detail** |  |  |
| **3 Indicators** |  |  |
| **prote�na\_cm1** | Continuous |  |
| **d�mero\_dm1** | Continuous |  |
| **procalcitonina\_m1** | Continuous |  |

#### Parameters

|  |  |  |  |  |
| --- | --- | --- | --- | --- |
| **Models for Indicators** |  |  |  |  |
|  | **Cluster1** | **Wald** | **p-value** | **R�** |
| **prote�na\_cm1** |  |  |  |  |
|  | . | . | . | 0.0000 |
| **d�mero\_dm1** |  |  |  |  |
|  | . | . | . | 0.0000 |
| **procalcitonina\_m1** |  |  |  |  |
|  | . | . | . | 0.0000 |
|  |  |  |  |  |
| **Intercepts** | **Overall** | **Wald** | **p-value** |  |
| **prote�na\_cm1** |  |  |  |  |
|  | 12.4777 | 938.4814 | 4.2e-206 |  |
| **d�mero\_dm1** |  |  |  |  |
|  | 2344.7232 | 534.6829 | 2.7e-118 |  |
| **procalcitonina\_m1** |  |  |  |  |
|  | 8.3882 | 79.8141 | 4.1e-19 |  |
|  |  |  |  |  |
| **Error Variances** | **Cluster1** |  |  |  |
| **prote�na\_cm1** | 119.1163 |  |  |  |
| **d�mero\_dm1** | 7382633.7996 |  |  |  |
| **procalcitonina\_m1** | 632.9695 |  |  |  |
|  |  |  |  |  |

#### Loadings

|  |  |  |
| --- | --- | --- |
| **Loadings** | **Clusters** | **R�** |
| **prote�na\_cm1** | 0.0000 | 0.0000 |
| **d�mero\_dm1** | 0.0000 | 0.0000 |
| **procalcitonina\_m1** | 0.0000 | 0.0000 |

#### Profile

|  |  |
| --- | --- |
|  | **Cluster1** |
| **Cluster Size** | 1.0000 |
| **Indicators** |  |
| **prote�na\_cm1** |  |
| **Mean** | 12.4777 |
| **d�mero\_dm1** |  |
| **Mean** | 2344.7232 |
| **procalcitonina\_m1** |  |
| **Mean** | 8.3882 |

#### ProbMeans

|  |  |
| --- | --- |
|  | **Cluster1** |
| **Overall** | 1.0000 |
| **Indicators** |  |
| **prote�na\_cm1** |  |
| **0.100 - 2.200** | 1.0000 |
| **2.300 - 6.500** | 1.0000 |
| **6.600 - 13** | 1.0000 |
| **13.10 - 22.60** | 1.0000 |
| **22.90 - 51.80** | 1.0000 |
| **d�mero\_dm1** |  |
| **25 - 870** | 1.0000 |
| **879 - 1308** | 1.0000 |
| **1311 - 1966** | 1.0000 |
| **1975 - 3182** | 1.0000 |
| **3190 - 3e+004** | 1.0000 |
| **procalcitonina\_m1** |  |
| **0.0400 - 0.0500** | 1.0000 |
| **0.0600 - 0.230** | 1.0000 |
| **0.240 - 0.790** | 1.0000 |
| **0.810 - 6.520** | 1.0000 |
| **6.580 - 200** | 1.0000 |

#### Bivariate Residuals

|  |  |  |  |
| --- | --- | --- | --- |
| **Indicators** | **prote�na\_cm1** | **d�mero\_dm1** | **procalcitonina\_m1** |
| **prote�na\_cm1** | . |  |  |
| **d�mero\_dm1** | 4.5218 | . |  |
| **procalcitonina\_m1** | 26.0619 | 18.3255 | . |

#### Classification

|  |  |  |  |  |  |
| --- | --- | --- | --- | --- | --- |
| **prote�na\_cm1** | **d�mero\_dm1** | **procalcitonina\_m1** | **ObsFreq** | **Modal** | **Cluster1** |
| 0.1 | 209 | 0.17 | 1.0000 | 1 | 1.0000 |
| 0.1 | 233 | 14.62 | 1.0000 | 1 | 1.0000 |
| 0.1 | 295 | 0.05 | 1.0000 | 1 | 1.0000 |
| 0.1 | 805 | 0.05 | 1.0000 | 1 | 1.0000 |
| 0.1 | 975 | 0.05 | 1.0000 | 1 | 1.0000 |
| 0.1 | 1010 | 0.28 | 1.0000 | 1 | 1.0000 |
| 0.1 | 1038 | 0.16 | 1.0000 | 1 | 1.0000 |
| 0.1 | 1117 | 0.1 | 1.0000 | 1 | 1.0000 |
| 0.1 | 1212 | 0.1 | 1.0000 | 1 | 1.0000 |
| 0.1 | 1454 | 0.05 | 1.0000 | 1 | 1.0000 |
| 0.1 | 1546 | 0.26 | 1.0000 | 1 | 1.0000 |
| 0.1 | 1604 | 0.11 | 1.0000 | 1 | 1.0000 |
| 0.1 | 1891 | 1.13 | 1.0000 | 1 | 1.0000 |
| 0.1 | 2648 | 0.05 | 1.0000 | 1 | 1.0000 |
| 0.1 | 4787 | 0.05 | 1.0000 | 1 | 1.0000 |
| 0.2 | 187 | 0.05 | 1.0000 | 1 | 1.0000 |
| 0.2 | 217 | 0.05 | 1.0000 | 1 | 1.0000 |
| 0.2 | 841 | 0.15 | 1.0000 | 1 | 1.0000 |
| 0.2 | 911 | 0.05 | 1.0000 | 1 | 1.0000 |
| 0.2 | 955 | 0.05 | 1.0000 | 1 | 1.0000 |
| 0.2 | 1046 | 0.27 | 1.0000 | 1 | 1.0000 |
| 0.2 | 1297 | 0.05 | 1.0000 | 1 | 1.0000 |
| 0.2 | 1690 | 0.05 | 1.0000 | 1 | 1.0000 |
| 0.2 | 1815 | 0.05 | 1.0000 | 1 | 1.0000 |
| 0.2 | 2571 | 0.05 | 1.0000 | 1 | 1.0000 |
| 0.2 | 2587 | 17.12 | 1.0000 | 1 | 1.0000 |
| 0.2 | 2803 | 0.05 | 1.0000 | 1 | 1.0000 |
| 0.2 | 2999 | 0.1 | 1.0000 | 1 | 1.0000 |
| 0.2 | 3107 | 1.82 | 1.0000 | 1 | 1.0000 |
| 0.2 | 4170 | 0.3 | 1.0000 | 1 | 1.0000 |
| 0.3 | 369 | 0.26 | 1.0000 | 1 | 1.0000 |
| 0.3 | 577 | 0.52 | 1.0000 | 1 | 1.0000 |
| 0.3 | 821 | 0.5 | 1.0000 | 1 | 1.0000 |
| 0.3 | 852 | 0.05 | 1.0000 | 1 | 1.0000 |
| 0.3 | 884 | 0.05 | 1.0000 | 1 | 1.0000 |
| 0.3 | 919 | 0.09 | 1.0000 | 1 | 1.0000 |
| 0.3 | 934 | 0.05 | 1.0000 | 1 | 1.0000 |
| 0.3 | 1097 | 0.05 | 1.0000 | 1 | 1.0000 |
| 0.3 | 2418 | 0.05 | 1.0000 | 1 | 1.0000 |
| 0.3 | 2980 | 0.22 | 1.0000 | 1 | 1.0000 |
| 0.3 | 3715 | 0.05 | 1.0000 | 1 | 1.0000 |
| 0.4 | 162 | 0.18 | 1.0000 | 1 | 1.0000 |
| 0.4 | 791 | 0.05 | 1.0000 | 1 | 1.0000 |
| 0.4 | 1062 | 0.27 | 1.0000 | 1 | 1.0000 |
| 0.4 | 1101 | 0.07 | 1.0000 | 1 | 1.0000 |
| 0.5 | 87 | 0.05 | 1.0000 | 1 | 1.0000 |
| 0.5 | 161 | 114.8 | 1.0000 | 1 | 1.0000 |
| 0.5 | 778 | 0.05 | 1.0000 | 1 | 1.0000 |
| 0.5 | 808 | 0.05 | 1.0000 | 1 | 1.0000 |
| 0.5 | 1180 | 1.11 | 1.0000 | 1 | 1.0000 |
| 0.5 | 1444 | 0.07 | 1.0000 | 1 | 1.0000 |
| 0.5 | 1617 | 0.1 | 1.0000 | 1 | 1.0000 |
| 0.5 | 1726 | 0.05 | 1.0000 | 1 | 1.0000 |
| 0.5 | 1966 | 0.05 | 1.0000 | 1 | 1.0000 |
| 0.5 | 20000 | 0.05 | 1.0000 | 1 | 1.0000 |
| 0.6 | 884 | 0.18 | 1.0000 | 1 | 1.0000 |
| 0.6 | 1147 | 0.06 | 1.0000 | 1 | 1.0000 |
| 0.6 | 1160 | 0.05 | 1.0000 | 1 | 1.0000 |
| 0.6 | 1187 | 0.05 | 1.0000 | 1 | 1.0000 |
| 0.6 | 1954 | 0.83 | 1.0000 | 1 | 1.0000 |
| 0.6 | 2799 | 0.2 | 1.0000 | 1 | 1.0000 |
| 0.6 | 3431 | 4.92 | 1.0000 | 1 | 1.0000 |
| 0.6 | 3675 | 0.21 | 1.0000 | 1 | 1.0000 |
| 0.6 | 10000 | 0.1 | 1.0000 | 1 | 1.0000 |
| 0.7 | 224 | 0.05 | 1.0000 | 1 | 1.0000 |
| 0.7 | 704 | 0.05 | 1.0000 | 1 | 1.0000 |
| 0.7 | 1360 | 0.05 | 1.0000 | 1 | 1.0000 |
| 0.7 | 1842 | 18.52 | 1.0000 | 1 | 1.0000 |
| 0.8 | 232 | 0.17 | 1.0000 | 1 | 1.0000 |
| 0.8 | 435 | 0.05 | 1.0000 | 1 | 1.0000 |
| 0.8 | 930 | 0.05 | 1.0000 | 1 | 1.0000 |
| 0.8 | 3070 | 2.92 | 1.0000 | 1 | 1.0000 |
| 0.9 | 302 | 0.05 | 1.0000 | 1 | 1.0000 |
| 0.9 | 1246 | 0.35 | 1.0000 | 1 | 1.0000 |
| 0.9 | 1406 | 0.05 | 1.0000 | 1 | 1.0000 |
| 0.9 | 1630 | 1.05 | 1.0000 | 1 | 1.0000 |
| 0.9 | 1771 | 0.21 | 1.0000 | 1 | 1.0000 |
| 0.9 | 2232 | 0.15 | 1.0000 | 1 | 1.0000 |
| 0.9 | 2882 | 0.05 | 1.0000 | 1 | 1.0000 |
| 0.9 | 3574 | 0.17 | 1.0000 | 1 | 1.0000 |
| 0.9 | 4947 | 124.3 | 1.0000 | 1 | 1.0000 |
| 1 | 249 | 0.05 | 1.0000 | 1 | 1.0000 |
| 1 | 737 | 0.37 | 1.0000 | 1 | 1.0000 |
| 1 | 1008 | 0.05 | 1.0000 | 1 | 1.0000 |
| 1 | 1559 | 0.05 | 1.0000 | 1 | 1.0000 |
| 1 | 1963 | 0.76 | 1.0000 | 1 | 1.0000 |
| 1 | 2069 | 0.27 | 1.0000 | 1 | 1.0000 |
| 1 | 3104 | 0.05 | 1.0000 | 1 | 1.0000 |
| 1.1 | 285 | 0.05 | 1.0000 | 1 | 1.0000 |
| 1.1 | 489 | 0.16 | 1.0000 | 1 | 1.0000 |
| 1.1 | 1313 | 0.05 | 1.0000 | 1 | 1.0000 |
| 1.1 | 1447 | 1.83 | 1.0000 | 1 | 1.0000 |
| 1.2 | 199 | 0.26 | 1.0000 | 1 | 1.0000 |
| 1.2 | 895 | 0.05 | 1.0000 | 1 | 1.0000 |
| 1.2 | 964 | 0.05 | 1.0000 | 1 | 1.0000 |
| 1.2 | 1546 | 0.05 | 1.0000 | 1 | 1.0000 |
| 1.2 | 1779 | 43.39 | 1.0000 | 1 | 1.0000 |
| 1.2 | 2122 | 0.13 | 1.0000 | 1 | 1.0000 |
| 1.2 | 2341 | 0.12 | 1.0000 | 1 | 1.0000 |
| 1.2 | 4093 | 0.05 | 1.0000 | 1 | 1.0000 |
| 1.2 | 5011 | 0.05 | 1.0000 | 1 | 1.0000 |
| 1.2 | 5018 | 3.51 | 1.0000 | 1 | 1.0000 |
| 1.3 | 1149 | 0.05 | 1.0000 | 1 | 1.0000 |
| 1.4 | 2659 | 0.05 | 1.0000 | 1 | 1.0000 |
| 1.4 | 3086 | 0.05 | 1.0000 | 1 | 1.0000 |
| 1.5 | 1084 | 0.05 | 1.0000 | 1 | 1.0000 |
| 1.5 | 1131 | 0.37 | 1.0000 | 1 | 1.0000 |
| 1.5 | 1323 | 0.05 | 1.0000 | 1 | 1.0000 |
| 1.5 | 2330 | 0.05 | 1.0000 | 1 | 1.0000 |
| 1.5 | 3452 | 4.18 | 1.0000 | 1 | 1.0000 |
| 1.5 | 3517 | 0.05 | 1.0000 | 1 | 1.0000 |
| 1.5 | 5036 | 1.77 | 1.0000 | 1 | 1.0000 |
| 1.6 | 1188 | 0.05 | 1.0000 | 1 | 1.0000 |
| 1.6 | 2004 | 0.05 | 1.0000 | 1 | 1.0000 |
| 1.6 | 3075 | 1.62 | 1.0000 | 1 | 1.0000 |
| 1.7 | 613 | 0.53 | 1.0000 | 1 | 1.0000 |
| 1.7 | 2419 | 0.21 | 1.0000 | 1 | 1.0000 |
| 1.7 | 4450 | 0.05 | 1.0000 | 1 | 1.0000 |
| 1.7 | 4893 | 0.05 | 1.0000 | 1 | 1.0000 |
| 1.8 | 1160 | 0.93 | 1.0000 | 1 | 1.0000 |
| 1.8 | 1172 | 0.05 | 1.0000 | 1 | 1.0000 |
| 1.8 | 1865 | 0.24 | 1.0000 | 1 | 1.0000 |
| 1.8 | 2171 | 0.08 | 1.0000 | 1 | 1.0000 |
| 1.8 | 2479 | 0.05 | 1.0000 | 1 | 1.0000 |
| 1.8 | 2506 | 0.05 | 1.0000 | 1 | 1.0000 |
| 1.9 | 1506 | 0.14 | 1.0000 | 1 | 1.0000 |
| 1.9 | 1646 | 0.05 | 1.0000 | 1 | 1.0000 |
| 1.9 | 1766 | 0.05 | 1.0000 | 1 | 1.0000 |
| 1.9 | 3178 | 0.05 | 1.0000 | 1 | 1.0000 |
| 2 | 891 | 0.06 | 1.0000 | 1 | 1.0000 |
| 2 | 1885 | 0.52 | 1.0000 | 1 | 1.0000 |
| 2.1 | 175 | 0.05 | 1.0000 | 1 | 1.0000 |
| 2.1 | 412 | 0.1 | 1.0000 | 1 | 1.0000 |
| 2.1 | 1712 | 0.05 | 1.0000 | 1 | 1.0000 |
| 2.1 | 1897 | 0.05 | 1.0000 | 1 | 1.0000 |
| 2.1 | 6030 | 0.05 | 1.0000 | 1 | 1.0000 |
| 2.2 | 254 | 0.18 | 1.0000 | 1 | 1.0000 |
| 2.2 | 349 | 0.05 | 1.0000 | 1 | 1.0000 |
| 2.2 | 707 | 0.32 | 1.0000 | 1 | 1.0000 |
| 2.2 | 845 | 0.17 | 1.0000 | 1 | 1.0000 |
| 2.2 | 895 | 1.02 | 1.0000 | 1 | 1.0000 |
| 2.2 | 986 | 0.05 | 1.0000 | 1 | 1.0000 |
| 2.2 | 2492 | 5.34 | 1.0000 | 1 | 1.0000 |
| 2.3 | 1188 | 0.05 | 1.0000 | 1 | 1.0000 |
| 2.3 | 1953 | 0.05 | 1.0000 | 1 | 1.0000 |
| 2.3 | 3345 | 0.08 | 1.0000 | 1 | 1.0000 |
| 2.3 | 3774 | 3.37 | 1.0000 | 1 | 1.0000 |
| 2.4 | 1250 | 0.08 | 1.0000 | 1 | 1.0000 |
| 2.4 | 1434 | 0.63 | 1.0000 | 1 | 1.0000 |
| 2.6 | 817 | 0.1 | 1.0000 | 1 | 1.0000 |
| 2.6 | 937 | 6.78 | 1.0000 | 1 | 1.0000 |
| 2.6 | 1182 | 0.28 | 1.0000 | 1 | 1.0000 |
| 2.6 | 1515 | 1.81 | 1.0000 | 1 | 1.0000 |
| 2.6 | 3269 | 0.34 | 1.0000 | 1 | 1.0000 |
| 2.7 | 924 | 40.46 | 1.0000 | 1 | 1.0000 |
| 2.7 | 1294 | 0.06 | 1.0000 | 1 | 1.0000 |
| 2.8 | 131 | 0.27 | 1.0000 | 1 | 1.0000 |
| 2.8 | 1007 | 4.43 | 1.0000 | 1 | 1.0000 |
| 2.8 | 2023 | 0.16 | 1.0000 | 1 | 1.0000 |
| 2.8 | 2620 | 0.05 | 1.0000 | 1 | 1.0000 |
| 2.9 | 269 | 0.28 | 1.0000 | 1 | 1.0000 |
| 2.9 | 467 | 0.17 | 1.0000 | 1 | 1.0000 |
| 2.9 | 704 | 0.05 | 1.0000 | 1 | 1.0000 |
| 2.9 | 791 | 0.26 | 1.0000 | 1 | 1.0000 |
| 2.9 | 1605 | 0.19 | 1.0000 | 1 | 1.0000 |
| 2.9 | 2477 | 0.24 | 1.0000 | 1 | 1.0000 |
| 2.9 | 4247 | 11.51 | 1.0000 | 1 | 1.0000 |
| 3 | 244 | 0.7 | 1.0000 | 1 | 1.0000 |
| 3 | 1011 | 0.33 | 1.0000 | 1 | 1.0000 |
| 3 | 1343 | 0.37 | 1.0000 | 1 | 1.0000 |
| 3 | 1701 | 0.1 | 1.0000 | 1 | 1.0000 |
| 3.1 | 402 | 0.05 | 1.0000 | 1 | 1.0000 |
| 3.1 | 2794 | 0.05 | 1.0000 | 1 | 1.0000 |
| 3.1 | 3956 | 0.05 | 1.0000 | 1 | 1.0000 |
| 3.2 | 1023 | 0.27 | 1.0000 | 1 | 1.0000 |
| 3.2 | 2511 | 0.26 | 1.0000 | 1 | 1.0000 |
| 3.3 | 882 | 2.65 | 1.0000 | 1 | 1.0000 |
| 3.3 | 2350 | 0.4 | 1.0000 | 1 | 1.0000 |
| 3.3 | 3175 | 11.49 | 1.0000 | 1 | 1.0000 |
| 3.4 | 1630 | 0.05 | 1.0000 | 1 | 1.0000 |
| 3.4 | 2037 | 0.05 | 1.0000 | 1 | 1.0000 |
| 3.4 | 8550 | 0.42 | 1.0000 | 1 | 1.0000 |
| 3.5 | 2849 | 0.06 | 1.0000 | 1 | 1.0000 |
| 3.5 | 3750 | 200 | 1.0000 | 1 | 1.0000 |
| 3.5 | 4986 | 2.82 | 1.0000 | 1 | 1.0000 |
| 3.6 | 316 | 0.18 | 1.0000 | 1 | 1.0000 |
| 3.6 | 900 | 4.32 | 1.0000 | 1 | 1.0000 |
| 3.6 | 1388 | 0.05 | 1.0000 | 1 | 1.0000 |
| 3.6 | 1474 | 0.05 | 1.0000 | 1 | 1.0000 |
| 3.6 | 2521 | 0.17 | 1.0000 | 1 | 1.0000 |
| 3.7 | 162 | 34.37 | 1.0000 | 1 | 1.0000 |
| 3.7 | 758 | 0.1 | 1.0000 | 1 | 1.0000 |
| 3.7 | 1730 | 0.05 | 1.0000 | 1 | 1.0000 |
| 3.7 | 2138 | 9.48 | 1.0000 | 1 | 1.0000 |
| 3.8 | 349 | 0.22 | 1.0000 | 1 | 1.0000 |
| 3.8 | 1340 | 0.85 | 1.0000 | 1 | 1.0000 |
| 3.8 | 2222 | 0.08 | 1.0000 | 1 | 1.0000 |
| 3.8 | 3344 | 0.21 | 1.0000 | 1 | 1.0000 |
| 3.9 | 125 | 0.2 | 1.0000 | 1 | 1.0000 |
| 3.9 | 199 | 0.15 | 1.0000 | 1 | 1.0000 |
| 3.9 | 1254 | 0.12 | 1.0000 | 1 | 1.0000 |
| 3.9 | 2318 | 0.05 | 1.0000 | 1 | 1.0000 |
| 4.1 | 789 | 15.58 | 1.0000 | 1 | 1.0000 |
| 4.1 | 816 | 0.05 | 1.0000 | 1 | 1.0000 |
| 4.1 | 1410 | 0.05 | 1.0000 | 1 | 1.0000 |
| 4.1 | 5150 | 17.69 | 1.0000 | 1 | 1.0000 |
| 4.2 | 590 | 0.41 | 1.0000 | 1 | 1.0000 |
| 4.2 | 2041 | 0.05 | 1.0000 | 1 | 1.0000 |
| 4.2 | 4937 | 0.16 | 1.0000 | 1 | 1.0000 |
| 4.3 | 956 | 0.05 | 1.0000 | 1 | 1.0000 |
| 4.4 | 483 | 0.28 | 1.0000 | 1 | 1.0000 |
| 4.4 | 950 | 8.2 | 1.0000 | 1 | 1.0000 |
| 4.4 | 2120 | 1.36 | 1.0000 | 1 | 1.0000 |
| 4.5 | 394 | 1.05 | 1.0000 | 1 | 1.0000 |
| 4.5 | 3500 | 27.92 | 1.0000 | 1 | 1.0000 |
| 4.5 | 3772 | 5.84 | 1.0000 | 1 | 1.0000 |
| 4.5 | 22290 | 0.36 | 1.0000 | 1 | 1.0000 |
| 4.6 | 366 | 0.29 | 1.0000 | 1 | 1.0000 |
| 4.6 | 5180 | 0.14 | 1.0000 | 1 | 1.0000 |
| 4.7 | 261 | 0.74 | 1.0000 | 1 | 1.0000 |
| 4.7 | 674 | 0.05 | 1.0000 | 1 | 1.0000 |
| 4.7 | 1258 | 0.59 | 1.0000 | 1 | 1.0000 |
| 4.7 | 1262 | 0.13 | 1.0000 | 1 | 1.0000 |
| 4.7 | 1747 | 0.6 | 1.0000 | 1 | 1.0000 |
| 4.8 | 242 | 0.05 | 1.0000 | 1 | 1.0000 |
| 4.8 | 1497 | 0.05 | 1.0000 | 1 | 1.0000 |
| 4.8 | 5790 | 15.07 | 1.0000 | 1 | 1.0000 |
| 4.9 | 1407 | 0.12 | 1.0000 | 1 | 1.0000 |
| 4.9 | 1646 | 0.13 | 1.0000 | 1 | 1.0000 |
| 4.9 | 1842 | 0.33 | 1.0000 | 1 | 1.0000 |
| 5 | 485 | 0.27 | 1.0000 | 1 | 1.0000 |
| 5 | 1255 | 0.08 | 1.0000 | 1 | 1.0000 |
| 5 | 2054 | 0.05 | 1.0000 | 1 | 1.0000 |
| 5 | 2854 | 3.92 | 1.0000 | 1 | 1.0000 |
| 5.1 | 179 | 0.05 | 1.0000 | 1 | 1.0000 |
| 5.1 | 1045 | 0.86 | 1.0000 | 1 | 1.0000 |
| 5.1 | 1455 | 3.9 | 1.0000 | 1 | 1.0000 |
| 5.1 | 1951 | 0.05 | 1.0000 | 1 | 1.0000 |
| 5.1 | 2142 | 0.96 | 1.0000 | 1 | 1.0000 |
| 5.1 | 4699 | 0.23 | 1.0000 | 1 | 1.0000 |
| 5.1 | 7150 | 5.91 | 1.0000 | 1 | 1.0000 |
| 5.2 | 907 | 0.51 | 1.0000 | 1 | 1.0000 |
| 5.2 | 1210 | 0.05 | 1.0000 | 1 | 1.0000 |
| 5.2 | 2367 | 0.05 | 1.0000 | 1 | 1.0000 |
| 5.3 | 255 | 1.69 | 1.0000 | 1 | 1.0000 |
| 5.3 | 1639 | 1.12 | 1.0000 | 1 | 1.0000 |
| 5.3 | 1712 | 0.49 | 1.0000 | 1 | 1.0000 |
| 5.3 | 3060 | 0.18 | 1.0000 | 1 | 1.0000 |
| 5.3 | 3847 | 0.13 | 1.0000 | 1 | 1.0000 |
| 5.3 | 10000 | 0.05 | 1.0000 | 1 | 1.0000 |
| 5.4 | 1358 | 0.1 | 1.0000 | 1 | 1.0000 |
| 5.4 | 1857 | 0.08 | 1.0000 | 1 | 1.0000 |
| 5.4 | 2018 | 22.09 | 1.0000 | 1 | 1.0000 |
| 5.5 | 25 | 0.05 | 1.0000 | 1 | 1.0000 |
| 5.5 | 817 | 0.14 | 1.0000 | 1 | 1.0000 |
| 5.5 | 959 | 0.2 | 1.0000 | 1 | 1.0000 |
| 5.5 | 1090 | 0.05 | 1.0000 | 1 | 1.0000 |
| 5.6 | 882 | 0.05 | 1.0000 | 1 | 1.0000 |
| 5.6 | 1208 | 0.1 | 1.0000 | 1 | 1.0000 |
| 5.7 | 573 | 0.12 | 1.0000 | 1 | 1.0000 |
| 5.7 | 954 | 0.05 | 1.0000 | 1 | 1.0000 |
| 5.7 | 2465 | 4.4 | 1.0000 | 1 | 1.0000 |
| 5.8 | 927 | 0.51 | 1.0000 | 1 | 1.0000 |
| 5.9 | 1348 | 0.05 | 1.0000 | 1 | 1.0000 |
| 5.9 | 1420 | 0.06 | 1.0000 | 1 | 1.0000 |
| 5.9 | 2136 | 0.41 | 1.0000 | 1 | 1.0000 |
| 5.9 | 2145 | 5.1 | 1.0000 | 1 | 1.0000 |
| 5.9 | 10000 | 5.07 | 1.0000 | 1 | 1.0000 |
| 6 | 3154 | 4.18 | 1.0000 | 1 | 1.0000 |
| 6 | 3182 | 0.05 | 1.0000 | 1 | 1.0000 |
| 6.1 | 338 | 0.17 | 1.0000 | 1 | 1.0000 |
| 6.1 | 14800 | 2.58 | 1.0000 | 1 | 1.0000 |
| 6.2 | 172 | 0.11 | 1.0000 | 1 | 1.0000 |
| 6.2 | 2268 | 0.1 | 1.0000 | 1 | 1.0000 |
| 6.2 | 3224 | 15 | 1.0000 | 1 | 1.0000 |
| 6.2 | 3774 | 0.43 | 1.0000 | 1 | 1.0000 |
| 6.2 | 15160 | 2.27 | 1.0000 | 1 | 1.0000 |
| 6.3 | 171 | 0.74 | 1.0000 | 1 | 1.0000 |
| 6.3 | 947 | 0.17 | 1.0000 | 1 | 1.0000 |
| 6.3 | 2276 | 0.5 | 1.0000 | 1 | 1.0000 |
| 6.3 | 4638 | 0.05 | 1.0000 | 1 | 1.0000 |
| 6.4 | 386 | 0.07 | 1.0000 | 1 | 1.0000 |
| 6.4 | 1038 | 1.1 | 1.0000 | 1 | 1.0000 |
| 6.5 | 121 | 0.35 | 1.0000 | 1 | 1.0000 |
| 6.5 | 1464 | 0.83 | 1.0000 | 1 | 1.0000 |
| 6.5 | 1769 | 0.44 | 1.0000 | 1 | 1.0000 |
| 6.5 | 2719 | 0.05 | 1.0000 | 1 | 1.0000 |
| 6.5 | 10000 | 29.02 | 1.0000 | 1 | 1.0000 |
| 6.6 | 390 | 2.22 | 1.0000 | 1 | 1.0000 |
| 6.6 | 1607 | 0.05 | 1.0000 | 1 | 1.0000 |
| 6.6 | 1961 | 0.06 | 1.0000 | 1 | 1.0000 |
| 6.6 | 5970 | 0.38 | 1.0000 | 1 | 1.0000 |
| 6.7 | 1167 | 0.05 | 1.0000 | 1 | 1.0000 |
| 6.7 | 1698 | 0.14 | 1.0000 | 1 | 1.0000 |
| 6.7 | 2988 | 0.14 | 1.0000 | 1 | 1.0000 |
| 6.7 | 3074 | 0.25 | 1.0000 | 1 | 1.0000 |
| 6.8 | 1265 | 0.39 | 1.0000 | 1 | 1.0000 |
| 6.8 | 4053 | 0.36 | 1.0000 | 1 | 1.0000 |
| 6.9 | 547 | 2.56 | 1.0000 | 1 | 1.0000 |
| 6.9 | 1190 | 0.19 | 1.0000 | 1 | 1.0000 |
| 6.9 | 1829 | 0.05 | 1.0000 | 1 | 1.0000 |
| 6.9 | 3737 | 2.11 | 1.0000 | 1 | 1.0000 |
| 7 | 378 | 0.05 | 1.0000 | 1 | 1.0000 |
| 7 | 810 | 0.05 | 1.0000 | 1 | 1.0000 |
| 7 | 1524 | 0.04 | 1.0000 | 1 | 1.0000 |
| 7.1 | 1048 | 0.32 | 1.0000 | 1 | 1.0000 |
| 7.1 | 1433 | 0.42 | 1.0000 | 1 | 1.0000 |
| 7.2 | 739 | 0.09 | 1.0000 | 1 | 1.0000 |
| 7.2 | 783 | 0.05 | 1.0000 | 1 | 1.0000 |
| 7.2 | 3855 | 2.9 | 1.0000 | 1 | 1.0000 |
| 7.3 | 702 | 0.05 | 1.0000 | 1 | 1.0000 |
| 7.3 | 2069 | 0.61 | 1.0000 | 1 | 1.0000 |
| 7.4 | 1412 | 0.05 | 1.0000 | 1 | 1.0000 |
| 7.4 | 7774 | 0.05 | 1.0000 | 1 | 1.0000 |
| 7.4 | 10000 | 0.12 | 1.0000 | 1 | 1.0000 |
| 7.5 | 457 | 0.05 | 1.0000 | 1 | 1.0000 |
| 7.5 | 1773 | 0.21 | 1.0000 | 1 | 1.0000 |
| 7.5 | 5018 | 0.05 | 1.0000 | 1 | 1.0000 |
| 7.5 | 5880 | 0.87 | 1.0000 | 1 | 1.0000 |
| 7.6 | 101 | 0.05 | 1.0000 | 1 | 1.0000 |
| 7.6 | 291 | 0.28 | 1.0000 | 1 | 1.0000 |
| 7.6 | 2092 | 6.62 | 1.0000 | 1 | 1.0000 |
| 7.7 | 1235 | 31.96 | 1.0000 | 1 | 1.0000 |
| 7.7 | 2173 | 0.23 | 1.0000 | 1 | 1.0000 |
| 7.7 | 2402 | 0.35 | 1.0000 | 1 | 1.0000 |
| 7.7 | 2985 | 0.05 | 1.0000 | 1 | 1.0000 |
| 7.7 | 10000 | 0.08 | 1.0000 | 1 | 1.0000 |
| 7.8 | 1313 | 29.95 | 1.0000 | 1 | 1.0000 |
| 7.8 | 1323 | 56.71 | 1.0000 | 1 | 1.0000 |
| 7.8 | 1562 | 0.06 | 1.0000 | 1 | 1.0000 |
| 7.8 | 2679 | 5.74 | 1.0000 | 1 | 1.0000 |
| 7.9 | 440 | 0.2 | 1.0000 | 1 | 1.0000 |
| 7.9 | 3510 | 0.48 | 1.0000 | 1 | 1.0000 |
| 7.9 | 4038 | 0.14 | 1.0000 | 1 | 1.0000 |
| 8 | 1311 | 0.29 | 1.0000 | 1 | 1.0000 |
| 8.1 | 1070 | 0.53 | 1.0000 | 1 | 1.0000 |
| 8.1 | 2124 | 2.24 | 1.0000 | 1 | 1.0000 |
| 8.2 | 215 | 0.14 | 1.0000 | 1 | 1.0000 |
| 8.2 | 1671 | 0.12 | 1.0000 | 1 | 1.0000 |
| 8.2 | 3908 | 2.83 | 1.0000 | 1 | 1.0000 |
| 8.3 | 981 | 43.49 | 1.0000 | 1 | 1.0000 |
| 8.3 | 1663 | 57.58 | 1.0000 | 1 | 1.0000 |
| 8.3 | 1768 | 1.97 | 1.0000 | 1 | 1.0000 |
| 8.3 | 4616 | 142.4 | 1.0000 | 1 | 1.0000 |
| 8.4 | 655 | 0.43 | 1.0000 | 1 | 1.0000 |
| 8.4 | 958 | 140.2 | 1.0000 | 1 | 1.0000 |
| 8.4 | 2389 | 113.6 | 1.0000 | 1 | 1.0000 |
| 8.4 | 2538 | 0.85 | 1.0000 | 1 | 1.0000 |
| 8.4 | 2810 | 3.4 | 1.0000 | 1 | 1.0000 |
| 8.5 | 3976 | 10.91 | 1.0000 | 1 | 1.0000 |
| 8.6 | 239 | 0.65 | 1.0000 | 1 | 1.0000 |
| 8.6 | 1500 | 0.05 | 1.0000 | 1 | 1.0000 |
| 8.7 | 903 | 0.25 | 1.0000 | 1 | 1.0000 |
| 8.9 | 1380 | 0.05 | 1.0000 | 1 | 1.0000 |
| 8.9 | 2205 | 0.05 | 1.0000 | 1 | 1.0000 |
| 9 | 1860 | 0.05 | 1.0000 | 1 | 1.0000 |
| 9 | 2569 | 0.15 | 1.0000 | 1 | 1.0000 |
| 9.1 | 988 | 0.1 | 1.0000 | 1 | 1.0000 |
| 9.1 | 8325 | 18.7 | 1.0000 | 1 | 1.0000 |
| 9.2 | 1301 | 31.75 | 1.0000 | 1 | 1.0000 |
| 9.2 | 1537 | 0.09 | 1.0000 | 1 | 1.0000 |
| 9.2 | 2642 | 9.1 | 1.0000 | 1 | 1.0000 |
| 9.2 | 2920 | 13.05 | 1.0000 | 1 | 1.0000 |
| 9.3 | 2627 | 1.26 | 1.0000 | 1 | 1.0000 |
| 9.3 | 5490 | 0.16 | 1.0000 | 1 | 1.0000 |
| 9.4 | 851 | 1.46 | 1.0000 | 1 | 1.0000 |
| 9.4 | 1525 | 7.15 | 1.0000 | 1 | 1.0000 |
| 9.6 | 704 | 3.2 | 1.0000 | 1 | 1.0000 |
| 9.6 | 1243 | 0.99 | 1.0000 | 1 | 1.0000 |
| 9.6 | 1381 | 2.14 | 1.0000 | 1 | 1.0000 |
| 9.6 | 17875 | 199.3 | 1.0000 | 1 | 1.0000 |
| 9.7 | 1073 | 0.13 | 1.0000 | 1 | 1.0000 |
| 9.9 | 1387 | 3.79 | 1.0000 | 1 | 1.0000 |
| 10.1 | 784 | 0.96 | 1.0000 | 1 | 1.0000 |
| 10.2 | 1141 | 0.06 | 1.0000 | 1 | 1.0000 |
| 10.3 | 295 | 0.06 | 1.0000 | 1 | 1.0000 |
| 10.3 | 331 | 0.15 | 1.0000 | 1 | 1.0000 |
| 10.3 | 699 | 0.2 | 1.0000 | 1 | 1.0000 |
| 10.3 | 2545 | 2.86 | 1.0000 | 1 | 1.0000 |
| 10.4 | 870 | 0.33 | 1.0000 | 1 | 1.0000 |
| 10.4 | 20000 | 0.53 | 1.0000 | 1 | 1.0000 |
| 10.5 | 848 | 0.05 | 1.0000 | 1 | 1.0000 |
| 10.5 | 1204 | 0.37 | 1.0000 | 1 | 1.0000 |
| 10.5 | 1230 | 0.05 | 1.0000 | 1 | 1.0000 |
| 10.5 | 2893 | 0.21 | 1.0000 | 1 | 1.0000 |
| 10.6 | 979 | 0.05 | 1.0000 | 1 | 1.0000 |
| 10.6 | 1929 | 0.75 | 1.0000 | 1 | 1.0000 |
| 10.6 | 3210 | 11.9 | 1.0000 | 1 | 1.0000 |
| 10.7 | 3122 | 20.35 | 1.0000 | 1 | 1.0000 |
| 10.8 | 417 | 0.05 | 1.0000 | 1 | 1.0000 |
| 10.8 | 3810 | 0.43 | 1.0000 | 1 | 1.0000 |
| 10.9 | 1280 | 0.18 | 1.0000 | 1 | 1.0000 |
| 10.9 | 2497 | 0.33 | 1.0000 | 1 | 1.0000 |
| 11 | 250 | 0.67 | 1.0000 | 1 | 1.0000 |
| 11.1 | 2532 | 10.14 | 1.0000 | 1 | 1.0000 |
| 11.1 | 3190 | 0.19 | 1.0000 | 1 | 1.0000 |
| 11.2 | 4482 | 0.07 | 1.0000 | 1 | 1.0000 |
| 11.3 | 803 | 0.05 | 1.0000 | 1 | 1.0000 |
| 11.4 | 912 | 0.05 | 1.0000 | 1 | 1.0000 |
| 11.4 | 2305 | 19.17 | 1.0000 | 1 | 1.0000 |
| 11.4 | 12480 | 0.09 | 1.0000 | 1 | 1.0000 |
| 11.5 | 1196 | 3.04 | 1.0000 | 1 | 1.0000 |
| 11.5 | 1319 | 0.05 | 1.0000 | 1 | 1.0000 |
| 11.6 | 4579 | 6.93 | 1.0000 | 1 | 1.0000 |
| 11.7 | 1003 | 2.63 | 1.0000 | 1 | 1.0000 |
| 11.7 | 1443 | 118.7 | 1.0000 | 1 | 1.0000 |
| 11.7 | 2912 | 0.29 | 1.0000 | 1 | 1.0000 |
| 11.8 | 2205 | 24.3 | 1.0000 | 1 | 1.0000 |
| 11.9 | 1130 | 0.05 | 1.0000 | 1 | 1.0000 |
| 11.9 | 3369 | 7.68 | 1.0000 | 1 | 1.0000 |
| 12 | 497 | 3.67 | 1.0000 | 1 | 1.0000 |
| 12 | 3504 | 39.56 | 1.0000 | 1 | 1.0000 |
| 12.1 | 1083 | 0.18 | 1.0000 | 1 | 1.0000 |
| 12.1 | 2047 | 0.24 | 1.0000 | 1 | 1.0000 |
| 12.2 | 4346 | 11.93 | 1.0000 | 1 | 1.0000 |
| 12.2 | 4732 | 0.07 | 1.0000 | 1 | 1.0000 |
| 12.3 | 1848 | 0.12 | 1.0000 | 1 | 1.0000 |
| 12.5 | 755 | 0.15 | 1.0000 | 1 | 1.0000 |
| 12.5 | 759 | 0.51 | 1.0000 | 1 | 1.0000 |
| 12.5 | 1340 | 0.05 | 1.0000 | 1 | 1.0000 |
| 12.5 | 2237 | 0.09 | 1.0000 | 1 | 1.0000 |
| 12.5 | 3416 | 0.05 | 1.0000 | 1 | 1.0000 |
| 12.5 | 7430 | 9.47 | 1.0000 | 1 | 1.0000 |
| 12.6 | 31 | 0.66 | 1.0000 | 1 | 1.0000 |
| 12.6 | 851 | 0.68 | 1.0000 | 1 | 1.0000 |
| 12.7 | 1701 | 0.54 | 1.0000 | 1 | 1.0000 |
| 12.8 | 1053 | 1.17 | 1.0000 | 1 | 1.0000 |
| 12.8 | 3730 | 0.44 | 1.0000 | 1 | 1.0000 |
| 12.9 | 190 | 0.55 | 1.0000 | 1 | 1.0000 |
| 12.9 | 1816 | 0.09 | 1.0000 | 1 | 1.0000 |
| 13 | 879 | 0.05 | 1.0000 | 1 | 1.0000 |
| 13 | 1229 | 0.3 | 1.0000 | 1 | 1.0000 |
| 13.1 | 1088 | 0.15 | 1.0000 | 1 | 1.0000 |
| 13.1 | 1246 | 0.38 | 1.0000 | 1 | 1.0000 |
| 13.1 | 1600 | 0.28 | 1.0000 | 1 | 1.0000 |
| 13.1 | 2070 | 0.12 | 1.0000 | 1 | 1.0000 |
| 13.2 | 788 | 25.91 | 1.0000 | 1 | 1.0000 |
| 13.2 | 3843 | 0.33 | 1.0000 | 1 | 1.0000 |
| 13.3 | 2833 | 0.05 | 1.0000 | 1 | 1.0000 |
| 13.4 | 501 | 0.09 | 1.0000 | 1 | 1.0000 |
| 13.6 | 1142 | 0.06 | 1.0000 | 1 | 1.0000 |
| 13.6 | 1350 | 0.86 | 1.0000 | 1 | 1.0000 |
| 13.6 | 1980 | 0.13 | 1.0000 | 1 | 1.0000 |
| 13.8 | 427 | 0.2 | 1.0000 | 1 | 1.0000 |
| 13.8 | 4830 | 0.12 | 1.0000 | 1 | 1.0000 |
| 14.1 | 4915 | 7.57 | 1.0000 | 1 | 1.0000 |
| 14.2 | 1216 | 11.06 | 1.0000 | 1 | 1.0000 |
| 14.2 | 2675 | 0.68 | 1.0000 | 1 | 1.0000 |
| 14.3 | 1721 | 1.47 | 1.0000 | 1 | 1.0000 |
| 14.4 | 974 | 0.2 | 1.0000 | 1 | 1.0000 |
| 14.4 | 1421 | 0.19 | 1.0000 | 1 | 1.0000 |
| 14.5 | 808 | 0.14 | 1.0000 | 1 | 1.0000 |
| 14.5 | 1944 | 0.81 | 1.0000 | 1 | 1.0000 |
| 14.6 | 1034 | 0.05 | 1.0000 | 1 | 1.0000 |
| 14.6 | 3350 | 0.51 | 1.0000 | 1 | 1.0000 |
| 14.7 | 270 | 0.98 | 1.0000 | 1 | 1.0000 |
| 14.7 | 844 | 80 | 1.0000 | 1 | 1.0000 |
| 14.7 | 1484 | 1.52 | 1.0000 | 1 | 1.0000 |
| 14.7 | 2464 | 0.51 | 1.0000 | 1 | 1.0000 |
| 14.8 | 271 | 26.81 | 1.0000 | 1 | 1.0000 |
| 14.9 | 337 | 0.33 | 1.0000 | 1 | 1.0000 |
| 14.9 | 1039 | 0.82 | 1.0000 | 1 | 1.0000 |
| 14.9 | 2114 | 0.29 | 1.0000 | 1 | 1.0000 |
| 15 | 1550 | 0.96 | 1.0000 | 1 | 1.0000 |
| 15.1 | 751 | 16.41 | 1.0000 | 1 | 1.0000 |
| 15.1 | 1814 | 0.14 | 1.0000 | 1 | 1.0000 |
| 15.1 | 2117 | 0.43 | 1.0000 | 1 | 1.0000 |
| 15.2 | 1106 | 1.87 | 1.0000 | 1 | 1.0000 |
| 15.2 | 1256 | 0.17 | 1.0000 | 1 | 1.0000 |
| 15.3 | 779 | 3.53 | 1.0000 | 1 | 1.0000 |
| 15.3 | 1975 | 12.61 | 1.0000 | 1 | 1.0000 |
| 15.3 | 2560 | 1.58 | 1.0000 | 1 | 1.0000 |
| 15.3 | 2709 | 0.28 | 1.0000 | 1 | 1.0000 |
| 15.5 | 2013 | 2.53 | 1.0000 | 1 | 1.0000 |
| 15.5 | 2054 | 0.31 | 1.0000 | 1 | 1.0000 |
| 15.6 | 3606 | 0.25 | 1.0000 | 1 | 1.0000 |
| 15.7 | 494 | 0.28 | 1.0000 | 1 | 1.0000 |
| 15.7 | 944 | 0.66 | 1.0000 | 1 | 1.0000 |
| 15.7 | 1283 | 0.05 | 1.0000 | 1 | 1.0000 |
| 15.7 | 1650 | 18.61 | 1.0000 | 1 | 1.0000 |
| 15.9 | 2911 | 2.25 | 1.0000 | 1 | 1.0000 |
| 15.9 | 3338 | 0.33 | 1.0000 | 1 | 1.0000 |
| 16 | 1623 | 0.13 | 1.0000 | 1 | 1.0000 |
| 16.1 | 514 | 0.41 | 1.0000 | 1 | 1.0000 |
| 16.1 | 2078 | 0.32 | 1.0000 | 1 | 1.0000 |
| 16.1 | 9310 | 5.39 | 1.0000 | 1 | 1.0000 |
| 16.2 | 2905 | 200 | 1.0000 | 1 | 1.0000 |
| 16.3 | 426 | 6.68 | 1.0000 | 1 | 1.0000 |
| 16.4 | 491 | 0.16 | 1.0000 | 1 | 1.0000 |
| 16.4 | 1379 | 0.08 | 1.0000 | 1 | 1.0000 |
| 16.4 | 33440 | 0.21 | 1.0000 | 1 | 1.0000 |
| 16.6 | 1194 | 7.39 | 1.0000 | 1 | 1.0000 |
| 16.6 | 4595 | 14.86 | 1.0000 | 1 | 1.0000 |
| 16.7 | 1308 | 0.05 | 1.0000 | 1 | 1.0000 |
| 16.7 | 1382 | 44.14 | 1.0000 | 1 | 1.0000 |
| 16.7 | 1702 | 0.06 | 1.0000 | 1 | 1.0000 |
| 16.7 | 4326 | 10.36 | 1.0000 | 1 | 1.0000 |
| 16.8 | 1623 | 0.05 | 1.0000 | 1 | 1.0000 |
| 16.8 | 2284 | 20.1 | 1.0000 | 1 | 1.0000 |
| 16.8 | 3000 | 0.4 | 1.0000 | 1 | 1.0000 |
| 16.9 | 444 | 0.56 | 1.0000 | 1 | 1.0000 |
| 17.1 | 702 | 0.19 | 1.0000 | 1 | 1.0000 |
| 17.1 | 2988 | 15.09 | 1.0000 | 1 | 1.0000 |
| 17.2 | 1028 | 0.23 | 1.0000 | 1 | 1.0000 |
| 17.2 | 1739 | 0.79 | 1.0000 | 1 | 1.0000 |
| 17.2 | 2918 | 0.08 | 1.0000 | 1 | 1.0000 |
| 17.2 | 2997 | 0.25 | 1.0000 | 1 | 1.0000 |
| 17.3 | 1427 | 0.25 | 1.0000 | 1 | 1.0000 |
| 17.4 | 1835 | 0.58 | 1.0000 | 1 | 1.0000 |
| 17.4 | 4236 | 0.5 | 1.0000 | 1 | 1.0000 |
| 17.7 | 603 | 0.42 | 1.0000 | 1 | 1.0000 |
| 17.7 | 1141 | 1.99 | 1.0000 | 1 | 1.0000 |
| 17.7 | 4812 | 3.35 | 1.0000 | 1 | 1.0000 |
| 17.8 | 2050 | 0.49 | 1.0000 | 1 | 1.0000 |
| 18 | 327 | 0.26 | 1.0000 | 1 | 1.0000 |
| 18 | 2055 | 60.68 | 1.0000 | 1 | 1.0000 |
| 18.1 | 1697 | 51.24 | 1.0000 | 1 | 1.0000 |
| 18.3 | 4573 | 0.69 | 1.0000 | 1 | 1.0000 |
| 18.3 | 6800 | 19.56 | 1.0000 | 1 | 1.0000 |
| 18.4 | 1136 | 1.45 | 1.0000 | 1 | 1.0000 |
| 18.4 | 2636 | 0.29 | 1.0000 | 1 | 1.0000 |
| 18.5 | 1764 | 17.23 | 1.0000 | 1 | 1.0000 |
| 18.6 | 311 | 1.89 | 1.0000 | 1 | 1.0000 |
| 18.6 | 2269 | 0.23 | 1.0000 | 1 | 1.0000 |
| 18.7 | 2334 | 11.71 | 1.0000 | 1 | 1.0000 |
| 18.7 | 2606 | 4.69 | 1.0000 | 1 | 1.0000 |
| 18.8 | 1547 | 5.6 | 1.0000 | 1 | 1.0000 |
| 19 | 1171 | 0.42 | 1.0000 | 1 | 1.0000 |
| 19.2 | 996 | 0.05 | 1.0000 | 1 | 1.0000 |
| 19.2 | 1007 | 5.76 | 1.0000 | 1 | 1.0000 |
| 19.3 | 20000 | 28.92 | 1.0000 | 1 | 1.0000 |
| 19.4 | 3517 | 11.43 | 1.0000 | 1 | 1.0000 |
| 19.4 | 5139 | 21.97 | 1.0000 | 1 | 1.0000 |
| 19.6 | 2936 | 20.09 | 1.0000 | 1 | 1.0000 |
| 19.7 | 1779 | 0.59 | 1.0000 | 1 | 1.0000 |
| 19.8 | 964 | 0.3 | 1.0000 | 1 | 1.0000 |
| 19.8 | 1400 | 0.47 | 1.0000 | 1 | 1.0000 |
| 19.8 | 1504 | 3.49 | 1.0000 | 1 | 1.0000 |
| 19.8 | 3632 | 0.24 | 1.0000 | 1 | 1.0000 |
| 19.8 | 5120 | 3.68 | 1.0000 | 1 | 1.0000 |
| 19.9 | 1170 | 3.1 | 1.0000 | 1 | 1.0000 |
| 20 | 829 | 5.63 | 1.0000 | 1 | 1.0000 |
| 20 | 3707 | 31.48 | 1.0000 | 1 | 1.0000 |
| 20.1 | 933 | 7.74 | 1.0000 | 1 | 1.0000 |
| 20.3 | 3138 | 0.23 | 1.0000 | 1 | 1.0000 |
| 20.4 | 1193 | 0.1 | 1.0000 | 1 | 1.0000 |
| 20.4 | 3670 | 0.05 | 1.0000 | 1 | 1.0000 |
| 20.4 | 6500 | 5.2 | 1.0000 | 1 | 1.0000 |
| 20.7 | 1114 | 5.24 | 1.0000 | 1 | 1.0000 |
| 20.8 | 700 | 0.23 | 1.0000 | 1 | 1.0000 |
| 20.8 | 1504 | 0.28 | 1.0000 | 1 | 1.0000 |
| 20.8 | 2142 | 6.52 | 1.0000 | 1 | 1.0000 |
| 21 | 2990 | 0.51 | 1.0000 | 1 | 1.0000 |
| 21.2 | 1014 | 2.82 | 1.0000 | 1 | 1.0000 |
| 21.2 | 2180 | 12.81 | 1.0000 | 1 | 1.0000 |
| 21.2 | 3173 | 53.78 | 1.0000 | 1 | 1.0000 |
| 21.3 | 4440 | 75.54 | 1.0000 | 1 | 1.0000 |
| 21.4 | 984 | 0.07 | 1.0000 | 1 | 1.0000 |
| 21.5 | 396 | 0.11 | 1.0000 | 1 | 1.0000 |
| 21.5 | 1231 | 0.32 | 1.0000 | 1 | 1.0000 |
| 21.6 | 737 | 0.11 | 1.0000 | 1 | 1.0000 |
| 21.6 | 1160 | 0.5 | 1.0000 | 1 | 1.0000 |
| 21.6 | 6910 | 21.97 | 1.0000 | 1 | 1.0000 |
| 21.7 | 1091 | 21.75 | 1.0000 | 1 | 1.0000 |
| 21.7 | 2368 | 0.22 | 1.0000 | 1 | 1.0000 |
| 21.8 | 8350 | 106.4 | 1.0000 | 1 | 1.0000 |
| 21.9 | 1562 | 12.36 | 1.0000 | 1 | 1.0000 |
| 22 | 3406 | 0.62 | 1.0000 | 1 | 1.0000 |
| 22.1 | 1227 | 4.49 | 1.0000 | 1 | 1.0000 |
| 22.1 | 3890 | 12.87 | 1.0000 | 1 | 1.0000 |
| 22.3 | 1104 | 1.02 | 1.0000 | 1 | 1.0000 |
| 22.4 | 605 | 0.25 | 1.0000 | 1 | 1.0000 |
| 22.4 | 3222 | 9.08 | 1.0000 | 1 | 1.0000 |
| 22.5 | 581 | 0.57 | 1.0000 | 1 | 1.0000 |
| 22.6 | 1394 | 3.03 | 1.0000 | 1 | 1.0000 |
| 22.6 | 3356 | 0.73 | 1.0000 | 1 | 1.0000 |
| 22.9 | 1229 | 0.99 | 1.0000 | 1 | 1.0000 |
| 22.9 | 2419 | 0.22 | 1.0000 | 1 | 1.0000 |
| 22.9 | 2909 | 3.59 | 1.0000 | 1 | 1.0000 |
| 22.9 | 10000 | 20 | 1.0000 | 1 | 1.0000 |
| 23 | 1653 | 0.96 | 1.0000 | 1 | 1.0000 |
| 23.1 | 993 | 1.22 | 1.0000 | 1 | 1.0000 |
| 23.1 | 1475 | 8.37 | 1.0000 | 1 | 1.0000 |
| 23.1 | 1955 | 79.35 | 1.0000 | 1 | 1.0000 |
| 23.2 | 4092 | 7.09 | 1.0000 | 1 | 1.0000 |
| 23.3 | 488 | 26.99 | 1.0000 | 1 | 1.0000 |
| 23.3 | 2135 | 0.86 | 1.0000 | 1 | 1.0000 |
| 23.4 | 1176 | 0.6 | 1.0000 | 1 | 1.0000 |
| 23.4 | 1312 | 120.4 | 1.0000 | 1 | 1.0000 |
| 23.4 | 1802 | 0.44 | 1.0000 | 1 | 1.0000 |
| 23.4 | 4819 | 0.35 | 1.0000 | 1 | 1.0000 |
| 23.5 | 294 | 0.08 | 1.0000 | 1 | 1.0000 |
| 23.5 | 3427 | 0.36 | 1.0000 | 1 | 1.0000 |
| 23.6 | 966 | 29.77 | 1.0000 | 1 | 1.0000 |
| 23.6 | 2680 | 0.59 | 1.0000 | 1 | 1.0000 |
| 23.7 | 1230 | 0.51 | 1.0000 | 1 | 1.0000 |
| 23.8 | 1137 | 1.18 | 1.0000 | 1 | 1.0000 |
| 24 | 1698 | 53.11 | 1.0000 | 1 | 1.0000 |
| 24 | 5560 | 0.17 | 1.0000 | 1 | 1.0000 |
| 24.1 | 2573 | 5.87 | 1.0000 | 1 | 1.0000 |
| 24.1 | 2605 | 0.26 | 1.0000 | 1 | 1.0000 |
| 24.4 | 790 | 200 | 1.0000 | 1 | 1.0000 |
| 24.4 | 3748 | 4.54 | 1.0000 | 1 | 1.0000 |
| 24.5 | 826 | 2.71 | 1.0000 | 1 | 1.0000 |
| 24.6 | 4634 | 27.65 | 1.0000 | 1 | 1.0000 |
| 24.6 | 10000 | 200 | 1.0000 | 1 | 1.0000 |
| 24.9 | 2700 | 42.34 | 1.0000 | 1 | 1.0000 |
| 24.9 | 3922 | 0.09 | 1.0000 | 1 | 1.0000 |
| 24.9 | 4316 | 11.03 | 1.0000 | 1 | 1.0000 |
| 24.9 | 6570 | 3.69 | 1.0000 | 1 | 1.0000 |
| 24.9 | 9510 | 0.32 | 1.0000 | 1 | 1.0000 |
| 25 | 708 | 20.12 | 1.0000 | 1 | 1.0000 |
| 25 | 934 | 25.01 | 1.0000 | 1 | 1.0000 |
| 25 | 1830 | 0.31 | 1.0000 | 1 | 1.0000 |
| 25 | 6110 | 18.08 | 1.0000 | 1 | 1.0000 |
| 25.1 | 2619 | 10.24 | 1.0000 | 1 | 1.0000 |
| 25.2 | 1364 | 13.71 | 1.0000 | 1 | 1.0000 |
| 25.2 | 3468 | 1.23 | 1.0000 | 1 | 1.0000 |
| 25.3 | 1717 | 0.88 | 1.0000 | 1 | 1.0000 |
| 25.4 | 2320 | 20.79 | 1.0000 | 1 | 1.0000 |
| 25.5 | 4420 | 0.45 | 1.0000 | 1 | 1.0000 |
| 25.6 | 1027 | 8.72 | 1.0000 | 1 | 1.0000 |
| 25.9 | 5950 | 1.75 | 1.0000 | 1 | 1.0000 |
| 26.1 | 47 | 0.18 | 1.0000 | 1 | 1.0000 |
| 26.1 | 2883 | 25.47 | 1.0000 | 1 | 1.0000 |
| 26.3 | 731 | 0.98 | 1.0000 | 1 | 1.0000 |
| 26.6 | 1076 | 0.98 | 1.0000 | 1 | 1.0000 |
| 26.6 | 3115 | 35.42 | 1.0000 | 1 | 1.0000 |
| 26.7 | 1167 | 34.45 | 1.0000 | 1 | 1.0000 |
| 26.8 | 647 | 7.56 | 1.0000 | 1 | 1.0000 |
| 27 | 793 | 1.13 | 1.0000 | 1 | 1.0000 |
| 27 | 3225 | 21.09 | 1.0000 | 1 | 1.0000 |
| 27.1 | 1120 | 0.85 | 1.0000 | 1 | 1.0000 |
| 27.1 | 1800 | 0.13 | 1.0000 | 1 | 1.0000 |
| 27.5 | 1487 | 2.97 | 1.0000 | 1 | 1.0000 |
| 27.5 | 2982 | 200 | 1.0000 | 1 | 1.0000 |
| 27.6 | 5270 | 20.52 | 1.0000 | 1 | 1.0000 |
| 27.8 | 3328 | 70.15 | 1.0000 | 1 | 1.0000 |
| 28 | 1593 | 0.31 | 1.0000 | 1 | 1.0000 |
| 28.2 | 2783 | 0.28 | 1.0000 | 1 | 1.0000 |
| 28.3 | 176 | 0.81 | 1.0000 | 1 | 1.0000 |
| 28.3 | 2033 | 0.2 | 1.0000 | 1 | 1.0000 |
| 28.4 | 2644 | 34.35 | 1.0000 | 1 | 1.0000 |
| 28.4 | 4633 | 61.35 | 1.0000 | 1 | 1.0000 |
| 28.5 | 817 | 0.55 | 1.0000 | 1 | 1.0000 |
| 28.5 | 1252 | 53.31 | 1.0000 | 1 | 1.0000 |
| 28.6 | 1244 | 1.79 | 1.0000 | 1 | 1.0000 |
| 28.6 | 1422 | 0.24 | 1.0000 | 1 | 1.0000 |
| 28.7 | 397 | 0.27 | 1.0000 | 1 | 1.0000 |
| 28.8 | 7230 | 12.2 | 1.0000 | 1 | 1.0000 |
| 28.9 | 3242 | 5.15 | 1.0000 | 1 | 1.0000 |
| 29 | 1587 | 1.72 | 1.0000 | 1 | 1.0000 |
| 29.1 | 2875 | 1.52 | 1.0000 | 1 | 1.0000 |
| 29.3 | 1887 | 1.01 | 1.0000 | 1 | 1.0000 |
| 29.5 | 1591 | 0.79 | 1.0000 | 1 | 1.0000 |
| 29.5 | 2185 | 49.36 | 1.0000 | 1 | 1.0000 |
| 29.5 | 2609 | 2.86 | 1.0000 | 1 | 1.0000 |
| 29.5 | 4502 | 71.2 | 1.0000 | 1 | 1.0000 |
| 29.7 | 986 | 4.14 | 1.0000 | 1 | 1.0000 |
| 29.9 | 249 | 0.69 | 1.0000 | 1 | 1.0000 |
| 29.9 | 964 | 32.05 | 1.0000 | 1 | 1.0000 |
| 29.9 | 1616 | 0.18 | 1.0000 | 1 | 1.0000 |
| 30 | 1384 | 31.73 | 1.0000 | 1 | 1.0000 |
| 30 | 4688 | 0.93 | 1.0000 | 1 | 1.0000 |
| 30.2 | 906 | 0.94 | 1.0000 | 1 | 1.0000 |
| 30.2 | 1712 | 1.68 | 1.0000 | 1 | 1.0000 |
| 30.5 | 762 | 1.82 | 1.0000 | 1 | 1.0000 |
| 30.7 | 2468 | 5.34 | 1.0000 | 1 | 1.0000 |
| 30.7 | 5020 | 24.37 | 1.0000 | 1 | 1.0000 |
| 30.8 | 1121 | 5.31 | 1.0000 | 1 | 1.0000 |
| 31 | 1569 | 23.95 | 1.0000 | 1 | 1.0000 |
| 31.1 | 2284 | 2.66 | 1.0000 | 1 | 1.0000 |
| 31.1 | 2765 | 98 | 1.0000 | 1 | 1.0000 |
| 31.4 | 4852 | 38.8 | 1.0000 | 1 | 1.0000 |
| 31.6 | 1687 | 29.18 | 1.0000 | 1 | 1.0000 |
| 31.9 | 1386 | 18.68 | 1.0000 | 1 | 1.0000 |
| 32.3 | 13230 | 8.93 | 1.0000 | 1 | 1.0000 |
| 32.5 | 1180 | 12.95 | 1.0000 | 1 | 1.0000 |
| 32.5 | 3037 | 0.47 | 1.0000 | 1 | 1.0000 |
| 32.5 | 4058 | 10.14 | 1.0000 | 1 | 1.0000 |
| 32.5 | 5880 | 56.79 | 1.0000 | 1 | 1.0000 |
| 32.8 | 2450 | 1.92 | 1.0000 | 1 | 1.0000 |
| 33.5 | 5000 | 0.37 | 1.0000 | 1 | 1.0000 |
| 33.8 | 4902 | 11.59 | 1.0000 | 1 | 1.0000 |
| 33.9 | 5082 | 0.14 | 1.0000 | 1 | 1.0000 |
| 34 | 1704 | 0.14 | 1.0000 | 1 | 1.0000 |
| 34.1 | 3498 | 6.58 | 1.0000 | 1 | 1.0000 |
| 34.3 | 1927 | 0.08 | 1.0000 | 1 | 1.0000 |
| 34.6 | 1788 | 12.27 | 1.0000 | 1 | 1.0000 |
| 34.7 | 4270 | 7.14 | 1.0000 | 1 | 1.0000 |
| 34.9 | 1206 | 1.45 | 1.0000 | 1 | 1.0000 |
| 35.7 | 830 | 7.25 | 1.0000 | 1 | 1.0000 |
| 36 | 729 | 2.63 | 1.0000 | 1 | 1.0000 |
| 36.3 | 3264 | 0.64 | 1.0000 | 1 | 1.0000 |
| 36.9 | 1046 | 5.26 | 1.0000 | 1 | 1.0000 |
| 37 | 1887 | 35.08 | 1.0000 | 1 | 1.0000 |
| 37.8 | 1507 | 4.5 | 1.0000 | 1 | 1.0000 |
| 37.9 | 2830 | 0.85 | 1.0000 | 1 | 1.0000 |
| 38 | 546 | 5.26 | 1.0000 | 1 | 1.0000 |
| 39 | 1836 | 12.52 | 1.0000 | 1 | 1.0000 |
| 39 | 2409 | 2.64 | 1.0000 | 1 | 1.0000 |
| 39.1 | 1807 | 55.61 | 1.0000 | 1 | 1.0000 |
| 39.3 | 1805 | 0.84 | 1.0000 | 1 | 1.0000 |
| 39.7 | 1274 | 1.78 | 1.0000 | 1 | 1.0000 |
| 40.1 | 1092 | 28.06 | 1.0000 | 1 | 1.0000 |
| 40.5 | 1813 | 25.54 | 1.0000 | 1 | 1.0000 |
| 40.5 | 7810 | 7.01 | 1.0000 | 1 | 1.0000 |
| 40.6 | 2821 | 2.06 | 1.0000 | 1 | 1.0000 |
| 40.8 | 737 | 1.52 | 1.0000 | 1 | 1.0000 |
| 41.4 | 799 | 1.05 | 1.0000 | 1 | 1.0000 |
| 41.6 | 5127 | 117.9 | 1.0000 | 1 | 1.0000 |
| 41.7 | 3725 | 77.96 | 1.0000 | 1 | 1.0000 |
| 42.5 | 2381 | 1.77 | 1.0000 | 1 | 1.0000 |
| 43.9 | 1771 | 0.76 | 1.0000 | 1 | 1.0000 |
| 44.6 | 2328 | 3.68 | 1.0000 | 1 | 1.0000 |
| 44.7 | 1160 | 0.26 | 1.0000 | 1 | 1.0000 |
| 45.1 | 1142 | 5.71 | 1.0000 | 1 | 1.0000 |
| 48.4 | 1266 | 1.47 | 1.0000 | 1 | 1.0000 |
| 51.8 | 8253 | 24.35 | 1.0000 | 1 | 1.0000 |

#### Model2 - LL = -11148.3941

|  |  |  |  |
| --- | --- | --- | --- |
| **2-Cluster Model** | | | |
|  |  |  |  |
| **Number of cases** | 719 |  |  |
| **Number of parameters (Npar)** | 13 |  |  |
| **Activated Constraints** | 0 |  |  |
| **Robustness Effect** | 1.9748 |  |  |
| **Random Seed** | 395671 |  |  |
| **Best Start Seed** | 1319630 |  |  |
|  |  |  |  |
| **Log-likelihood Statistics** |  |  |  |
| **Log-likelihood (LL)** | -11148.3941 |  |  |
| **Log-prior** | -126.3664 |  |  |
| **Log-posterior** | -11274.7605 |  |  |
| **BIC (based on LL)** | 22382.3004 |  |  |
| **AIC (based on LL)** | 22322.7882 |  |  |
| **AIC3 (based on LL)** | 22335.7882 |  |  |
| **CAIC (based on LL)** | 22395.3004 |  |  |
|  |  |  |  |
| **Classification Statistics** | **Clusters** |  |  |
| **Classification errors** | 0.0179 |  |  |
| **Reduction of errors (Lambda)** | 0.9338 |  |  |
| **Entropy R-squared** | 0.9140 |  |  |
| **Standard R-squared** | 0.9323 |  |  |
| **Classification log-likelihood** | -11184.4471 |  |  |
| **AWE** | 22578.9186 |  |  |
|  |  |  |  |
| **Classification Table** | **Modal** |  |  |
| **Probabilistic** | **Cluster1** | **Cluster2** | **Total** |
| **Cluster1** | 520.6429 | 4.4991 | 525.1420 |
| **Cluster2** | 8.3571 | 185.5009 | 193.8580 |
| **Total** | 529.0000 | 190.0000 | 719.0000 |
|  |  |  |  |
| **Files** |  |  |  |
| **Infile** | C:\Users\Fabian Jaimes\Documents\Johana\Disepsis\DISEPSIS\Clases latentes\Clases latentes\_dic16\disepsis\_definitiva\_CL.sav | | |
|  |  |  |  |
| **Variable Detail** |  |  |  |
| **3 Indicators** |  |  |  |
| **prote�na\_cm1** | Continuous |  |  |
| **d�mero\_dm1** | Continuous |  |  |
| **procalcitonina\_m1** | Continuous |  |  |

#### Parameters

|  |  |  |  |  |  |
| --- | --- | --- | --- | --- | --- |
| **Models for Indicators** |  |  |  |  |  |
|  | **Cluster1** | **Cluster2** | **Wald** | **p-value** | **R�** |
| **prote�na\_cm1** |  |  |  |  |  |
|  | -4.2676 | 4.2676 | 65.8978 | 4.7e-16 | 0.1205 |
| **d�mero\_dm1** |  |  |  |  |  |
|  | -1208.1402 | 1208.1402 | 54.0829 | 1.9e-13 | 0.1559 |
| **procalcitonina\_m1** |  |  |  |  |  |
|  | -14.4753 | 14.4753 | 80.4696 | 3.0e-19 | 0.2610 |
|  |  |  |  |  |  |
| **Intercepts** | **Overall** | **Wald** | **p-value** |  |  |
| **prote�na\_cm1** |  |  |  |  |  |
|  | 14.4441 | 949.1949 | 2.0e-208 |  |  |
| **d�mero\_dm1** |  |  |  |  |  |
|  | 2901.3818 | 295.3452 | 3.4e-66 |  |  |
| **procalcitonina\_m1** |  |  |  |  |  |
|  | 15.0578 | 85.4164 | 2.4e-20 |  |  |
|  |  |  |  |  |  |
| **Error Variances** | **Cluster1** | **Cluster2** |  |  |  |
| **prote�na\_cm1** | 95.3033 | 130.4471 |  |  |  |
| **d�mero\_dm1** | 1350566.8863 | 19443105.1305 |  |  |  |
| **procalcitonina\_m1** | 1.4201 | 1730.4181 |  |  |  |
|  |  |  |  |  |  |
|  |  |  |  |  |  |
| **Model for Clusters** |  |  |  |  |  |
| **Intercept** | **Cluster1** | **Cluster2** | **Wald** | **p-value** |  |
|  | 0.4975 | -0.4975 | 114.8149 | 8.6e-27 |  |
|  |  |  |  |  |  |

#### Loadings

|  |  |  |
| --- | --- | --- |
| **Loadings** | **Clusters** | **R�** |
| **prote�na\_cm1** | 0.3471 | 0.1205 |
| **d�mero\_dm1** | 0.3949 | 0.1559 |
| **procalcitonina\_m1** | 0.5109 | 0.2610 |

#### Profile

|  |  |  |
| --- | --- | --- |
|  | **Cluster1** | **Cluster2** |
| **Cluster Size** | 0.7301 | 0.2699 |
| **Indicators** |  |  |
| **prote�na\_cm1** |  |  |
| **Mean** | 10.1765 | 18.7117 |
| **d�mero\_dm1** |  |  |
| **Mean** | 1693.2416 | 4109.5221 |
| **procalcitonina\_m1** |  |  |
| **Mean** | 0.5825 | 29.5330 |

#### ProbMeans

|  |  |  |
| --- | --- | --- |
|  | **Cluster1** | **Cluster2** |
| **Overall** | 0.7301 | 0.2699 |
| **Indicators** |  |  |
| **prote�na\_cm1** |  |  |
| **0.100 - 2.200** | 0.9224 | 0.0776 |
| **2.300 - 6.500** | 0.8273 | 0.1727 |
| **6.600 - 13** | 0.7588 | 0.2412 |
| **13.10 - 22.60** | 0.6744 | 0.3256 |
| **22.90 - 51.80** | 0.4670 | 0.5330 |
| **d�mero\_dm1** |  |  |
| **25 - 870** | 0.8841 | 0.1159 |
| **879 - 1308** | 0.8234 | 0.1766 |
| **1311 - 1966** | 0.8034 | 0.1966 |
| **1975 - 3182** | 0.7359 | 0.2641 |
| **3190 - 3e+004** | 0.4059 | 0.5941 |
| **procalcitonina\_m1** |  |  |
| **0.0400 - 0.0500** | 0.9723 | 0.0277 |
| **0.0600 - 0.230** | 0.9519 | 0.0481 |
| **0.240 - 0.790** | 0.9599 | 0.0401 |
| **0.810 - 6.520** | 0.7692 | 0.2308 |
| **6.580 - 200** | 0.0000 | 1.0000 |

#### Bivariate Residuals

|  |  |  |  |
| --- | --- | --- | --- |
| **Indicators** | **prote�na\_cm1** | **d�mero\_dm1** | **procalcitonina\_m1** |
| **prote�na\_cm1** | . |  |  |
| **d�mero\_dm1** | 3.4800 | . |  |
| **procalcitonina\_m1** | 13.1199 | 1.4823 | . |

#### Classification

|  |  |  |  |  |  |  |
| --- | --- | --- | --- | --- | --- | --- |
| **prote�na\_cm1** | **d�mero\_dm1** | **procalcitonina\_m1** | **ObsFreq** | **Modal** | **Cluster1** | **Cluster2** |
| 0.1 | 209 | 0.17 | 1.0000 | 1 | 0.9986 | 0.0014 |
| 0.1 | 233 | 14.62 | 1.0000 | 2 | 0.0000 | 1.0000 |
| 0.1 | 295 | 0.05 | 1.0000 | 1 | 0.9987 | 0.0013 |
| 0.1 | 805 | 0.05 | 1.0000 | 1 | 0.9991 | 0.0009 |
| 0.1 | 975 | 0.05 | 1.0000 | 1 | 0.9991 | 0.0009 |
| 0.1 | 1010 | 0.28 | 1.0000 | 1 | 0.9992 | 0.0008 |
| 0.1 | 1038 | 0.16 | 1.0000 | 1 | 0.9992 | 0.0008 |
| 0.1 | 1117 | 0.1 | 1.0000 | 1 | 0.9992 | 0.0008 |
| 0.1 | 1212 | 0.1 | 1.0000 | 1 | 0.9992 | 0.0008 |
| 0.1 | 1454 | 0.05 | 1.0000 | 1 | 0.9992 | 0.0008 |
| 0.1 | 1546 | 0.26 | 1.0000 | 1 | 0.9993 | 0.0007 |
| 0.1 | 1604 | 0.11 | 1.0000 | 1 | 0.9992 | 0.0008 |
| 0.1 | 1891 | 1.13 | 1.0000 | 1 | 0.9992 | 0.0008 |
| 0.1 | 2648 | 0.05 | 1.0000 | 1 | 0.9988 | 0.0012 |
| 0.1 | 4787 | 0.05 | 1.0000 | 1 | 0.9693 | 0.0307 |
| 0.2 | 187 | 0.05 | 1.0000 | 1 | 0.9986 | 0.0014 |
| 0.2 | 217 | 0.05 | 1.0000 | 1 | 0.9986 | 0.0014 |
| 0.2 | 841 | 0.15 | 1.0000 | 1 | 0.9991 | 0.0009 |
| 0.2 | 911 | 0.05 | 1.0000 | 1 | 0.9991 | 0.0009 |
| 0.2 | 955 | 0.05 | 1.0000 | 1 | 0.9991 | 0.0009 |
| 0.2 | 1046 | 0.27 | 1.0000 | 1 | 0.9992 | 0.0008 |
| 0.2 | 1297 | 0.05 | 1.0000 | 1 | 0.9992 | 0.0008 |
| 0.2 | 1690 | 0.05 | 1.0000 | 1 | 0.9992 | 0.0008 |
| 0.2 | 1815 | 0.05 | 1.0000 | 1 | 0.9992 | 0.0008 |
| 0.2 | 2571 | 0.05 | 1.0000 | 1 | 0.9988 | 0.0012 |
| 0.2 | 2587 | 17.12 | 1.0000 | 2 | 0.0000 | 1.0000 |
| 0.2 | 2803 | 0.05 | 1.0000 | 1 | 0.9986 | 0.0014 |
| 0.2 | 2999 | 0.1 | 1.0000 | 1 | 0.9983 | 0.0017 |
| 0.2 | 3107 | 1.82 | 1.0000 | 1 | 0.9970 | 0.0030 |
| 0.2 | 4170 | 0.3 | 1.0000 | 1 | 0.9917 | 0.0083 |
| 0.3 | 369 | 0.26 | 1.0000 | 1 | 0.9988 | 0.0012 |
| 0.3 | 577 | 0.52 | 1.0000 | 1 | 0.9990 | 0.0010 |
| 0.3 | 821 | 0.5 | 1.0000 | 1 | 0.9991 | 0.0009 |
| 0.3 | 852 | 0.05 | 1.0000 | 1 | 0.9991 | 0.0009 |
| 0.3 | 884 | 0.05 | 1.0000 | 1 | 0.9991 | 0.0009 |
| 0.3 | 919 | 0.09 | 1.0000 | 1 | 0.9991 | 0.0009 |
| 0.3 | 934 | 0.05 | 1.0000 | 1 | 0.9991 | 0.0009 |
| 0.3 | 1097 | 0.05 | 1.0000 | 1 | 0.9992 | 0.0008 |
| 0.3 | 2418 | 0.05 | 1.0000 | 1 | 0.9989 | 0.0011 |
| 0.3 | 2980 | 0.22 | 1.0000 | 1 | 0.9984 | 0.0016 |
| 0.3 | 3715 | 0.05 | 1.0000 | 1 | 0.9958 | 0.0042 |
| 0.4 | 162 | 0.18 | 1.0000 | 1 | 0.9986 | 0.0014 |
| 0.4 | 791 | 0.05 | 1.0000 | 1 | 0.9990 | 0.0010 |
| 0.4 | 1062 | 0.27 | 1.0000 | 1 | 0.9992 | 0.0008 |
| 0.4 | 1101 | 0.07 | 1.0000 | 1 | 0.9992 | 0.0008 |
| 0.5 | 87 | 0.05 | 1.0000 | 1 | 0.9984 | 0.0016 |
| 0.5 | 161 | 114.8 | 1.0000 | 2 | 0.0000 | 1.0000 |
| 0.5 | 778 | 0.05 | 1.0000 | 1 | 0.9990 | 0.0010 |
| 0.5 | 808 | 0.05 | 1.0000 | 1 | 0.9991 | 0.0009 |
| 0.5 | 1180 | 1.11 | 1.0000 | 1 | 0.9992 | 0.0008 |
| 0.5 | 1444 | 0.07 | 1.0000 | 1 | 0.9992 | 0.0008 |
| 0.5 | 1617 | 0.1 | 1.0000 | 1 | 0.9992 | 0.0008 |
| 0.5 | 1726 | 0.05 | 1.0000 | 1 | 0.9992 | 0.0008 |
| 0.5 | 1966 | 0.05 | 1.0000 | 1 | 0.9991 | 0.0009 |
| 0.5 | 20000 | 0.05 | 1.0000 | 2 | 0.0000 | 1.0000 |
| 0.6 | 884 | 0.18 | 1.0000 | 1 | 0.9991 | 0.0009 |
| 0.6 | 1147 | 0.06 | 1.0000 | 1 | 0.9992 | 0.0008 |
| 0.6 | 1160 | 0.05 | 1.0000 | 1 | 0.9992 | 0.0008 |
| 0.6 | 1187 | 0.05 | 1.0000 | 1 | 0.9992 | 0.0008 |
| 0.6 | 1954 | 0.83 | 1.0000 | 1 | 0.9992 | 0.0008 |
| 0.6 | 2799 | 0.2 | 1.0000 | 1 | 0.9986 | 0.0014 |
| 0.6 | 3431 | 4.92 | 1.0000 | 2 | 0.3228 | 0.6772 |
| 0.6 | 3675 | 0.21 | 1.0000 | 1 | 0.9962 | 0.0038 |
| 0.6 | 10000 | 0.1 | 1.0000 | 2 | 0.0000 | 1.0000 |
| 0.7 | 224 | 0.05 | 1.0000 | 1 | 0.9986 | 0.0014 |
| 0.7 | 704 | 0.05 | 1.0000 | 1 | 0.9990 | 0.0010 |
| 0.7 | 1360 | 0.05 | 1.0000 | 1 | 0.9992 | 0.0008 |
| 0.7 | 1842 | 18.52 | 1.0000 | 2 | 0.0000 | 1.0000 |
| 0.8 | 232 | 0.17 | 1.0000 | 1 | 0.9986 | 0.0014 |
| 0.8 | 435 | 0.05 | 1.0000 | 1 | 0.9988 | 0.0012 |
| 0.8 | 930 | 0.05 | 1.0000 | 1 | 0.9991 | 0.0009 |
| 0.8 | 3070 | 2.92 | 1.0000 | 1 | 0.9880 | 0.0120 |
| 0.9 | 302 | 0.05 | 1.0000 | 1 | 0.9987 | 0.0013 |
| 0.9 | 1246 | 0.35 | 1.0000 | 1 | 0.9992 | 0.0008 |
| 0.9 | 1406 | 0.05 | 1.0000 | 1 | 0.9992 | 0.0008 |
| 0.9 | 1630 | 1.05 | 1.0000 | 1 | 0.9992 | 0.0008 |
| 0.9 | 1771 | 0.21 | 1.0000 | 1 | 0.9992 | 0.0008 |
| 0.9 | 2232 | 0.15 | 1.0000 | 1 | 0.9991 | 0.0009 |
| 0.9 | 2882 | 0.05 | 1.0000 | 1 | 0.9985 | 0.0015 |
| 0.9 | 3574 | 0.17 | 1.0000 | 1 | 0.9966 | 0.0034 |
| 0.9 | 4947 | 124.3 | 1.0000 | 2 | 0.0000 | 1.0000 |
| 1 | 249 | 0.05 | 1.0000 | 1 | 0.9986 | 0.0014 |
| 1 | 737 | 0.37 | 1.0000 | 1 | 0.9991 | 0.0009 |
| 1 | 1008 | 0.05 | 1.0000 | 1 | 0.9991 | 0.0009 |
| 1 | 1559 | 0.05 | 1.0000 | 1 | 0.9992 | 0.0008 |
| 1 | 1963 | 0.76 | 1.0000 | 1 | 0.9992 | 0.0008 |
| 1 | 2069 | 0.27 | 1.0000 | 1 | 0.9991 | 0.0009 |
| 1 | 3104 | 0.05 | 1.0000 | 1 | 0.9981 | 0.0019 |
| 1.1 | 285 | 0.05 | 1.0000 | 1 | 0.9986 | 0.0014 |
| 1.1 | 489 | 0.16 | 1.0000 | 1 | 0.9989 | 0.0011 |
| 1.1 | 1313 | 0.05 | 1.0000 | 1 | 0.9992 | 0.0008 |
| 1.1 | 1447 | 1.83 | 1.0000 | 1 | 0.9987 | 0.0013 |
| 1.2 | 199 | 0.26 | 1.0000 | 1 | 0.9986 | 0.0014 |
| 1.2 | 895 | 0.05 | 1.0000 | 1 | 0.9991 | 0.0009 |
| 1.2 | 964 | 0.05 | 1.0000 | 1 | 0.9991 | 0.0009 |
| 1.2 | 1546 | 0.05 | 1.0000 | 1 | 0.9992 | 0.0008 |
| 1.2 | 1779 | 43.39 | 1.0000 | 2 | 0.0000 | 1.0000 |
| 1.2 | 2122 | 0.13 | 1.0000 | 1 | 0.9991 | 0.0009 |
| 1.2 | 2341 | 0.12 | 1.0000 | 1 | 0.9990 | 0.0010 |
| 1.2 | 4093 | 0.05 | 1.0000 | 1 | 0.9919 | 0.0081 |
| 1.2 | 5011 | 0.05 | 1.0000 | 1 | 0.9472 | 0.0528 |
| 1.2 | 5018 | 3.51 | 1.0000 | 2 | 0.4745 | 0.5255 |
| 1.3 | 1149 | 0.05 | 1.0000 | 1 | 0.9991 | 0.0009 |
| 1.4 | 2659 | 0.05 | 1.0000 | 1 | 0.9987 | 0.0013 |
| 1.4 | 3086 | 0.05 | 1.0000 | 1 | 0.9981 | 0.0019 |
| 1.5 | 1084 | 0.05 | 1.0000 | 1 | 0.9991 | 0.0009 |
| 1.5 | 1131 | 0.37 | 1.0000 | 1 | 0.9992 | 0.0008 |
| 1.5 | 1323 | 0.05 | 1.0000 | 1 | 0.9992 | 0.0008 |
| 1.5 | 2330 | 0.05 | 1.0000 | 1 | 0.9990 | 0.0010 |
| 1.5 | 3452 | 4.18 | 1.0000 | 1 | 0.7814 | 0.2186 |
| 1.5 | 3517 | 0.05 | 1.0000 | 1 | 0.9967 | 0.0033 |
| 1.5 | 5036 | 1.77 | 1.0000 | 1 | 0.9161 | 0.0839 |
| 1.6 | 1188 | 0.05 | 1.0000 | 1 | 0.9991 | 0.0009 |
| 1.6 | 2004 | 0.05 | 1.0000 | 1 | 0.9991 | 0.0009 |
| 1.6 | 3075 | 1.62 | 1.0000 | 1 | 0.9974 | 0.0026 |
| 1.7 | 613 | 0.53 | 1.0000 | 1 | 0.9990 | 0.0010 |
| 1.7 | 2419 | 0.21 | 1.0000 | 1 | 0.9989 | 0.0011 |
| 1.7 | 4450 | 0.05 | 1.0000 | 1 | 0.9839 | 0.0161 |
| 1.7 | 4893 | 0.05 | 1.0000 | 1 | 0.9588 | 0.0412 |
| 1.8 | 1160 | 0.93 | 1.0000 | 1 | 0.9992 | 0.0008 |
| 1.8 | 1172 | 0.05 | 1.0000 | 1 | 0.9991 | 0.0009 |
| 1.8 | 1865 | 0.24 | 1.0000 | 1 | 0.9992 | 0.0008 |
| 1.8 | 2171 | 0.08 | 1.0000 | 1 | 0.9990 | 0.0010 |
| 1.8 | 2479 | 0.05 | 1.0000 | 1 | 0.9988 | 0.0012 |
| 1.8 | 2506 | 0.05 | 1.0000 | 1 | 0.9988 | 0.0012 |
| 1.9 | 1506 | 0.14 | 1.0000 | 1 | 0.9992 | 0.0008 |
| 1.9 | 1646 | 0.05 | 1.0000 | 1 | 0.9991 | 0.0009 |
| 1.9 | 1766 | 0.05 | 1.0000 | 1 | 0.9991 | 0.0009 |
| 1.9 | 3178 | 0.05 | 1.0000 | 1 | 0.9978 | 0.0022 |
| 2 | 891 | 0.06 | 1.0000 | 1 | 0.9990 | 0.0010 |
| 2 | 1885 | 0.52 | 1.0000 | 1 | 0.9992 | 0.0008 |
| 2.1 | 175 | 0.05 | 1.0000 | 1 | 0.9984 | 0.0016 |
| 2.1 | 412 | 0.1 | 1.0000 | 1 | 0.9987 | 0.0013 |
| 2.1 | 1712 | 0.05 | 1.0000 | 1 | 0.9991 | 0.0009 |
| 2.1 | 1897 | 0.05 | 1.0000 | 1 | 0.9991 | 0.0009 |
| 2.1 | 6030 | 0.05 | 1.0000 | 1 | 0.5092 | 0.4908 |
| 2.2 | 254 | 0.18 | 1.0000 | 1 | 0.9986 | 0.0014 |
| 2.2 | 349 | 0.05 | 1.0000 | 1 | 0.9986 | 0.0014 |
| 2.2 | 707 | 0.32 | 1.0000 | 1 | 0.9990 | 0.0010 |
| 2.2 | 845 | 0.17 | 1.0000 | 1 | 0.9990 | 0.0010 |
| 2.2 | 895 | 1.02 | 1.0000 | 1 | 0.9990 | 0.0010 |
| 2.2 | 986 | 0.05 | 1.0000 | 1 | 0.9991 | 0.0009 |
| 2.2 | 2492 | 5.34 | 1.0000 | 2 | 0.2281 | 0.7719 |
| 2.3 | 1188 | 0.05 | 1.0000 | 1 | 0.9991 | 0.0009 |
| 2.3 | 1953 | 0.05 | 1.0000 | 1 | 0.9991 | 0.0009 |
| 2.3 | 3345 | 0.08 | 1.0000 | 1 | 0.9973 | 0.0027 |
| 2.3 | 3774 | 3.37 | 1.0000 | 1 | 0.9313 | 0.0687 |
| 2.4 | 1250 | 0.08 | 1.0000 | 1 | 0.9991 | 0.0009 |
| 2.4 | 1434 | 0.63 | 1.0000 | 1 | 0.9992 | 0.0008 |
| 2.6 | 817 | 0.1 | 1.0000 | 1 | 0.9990 | 0.0010 |
| 2.6 | 937 | 6.78 | 1.0000 | 2 | 0.0014 | 0.9986 |
| 2.6 | 1182 | 0.28 | 1.0000 | 1 | 0.9992 | 0.0008 |
| 2.6 | 1515 | 1.81 | 1.0000 | 1 | 0.9986 | 0.0014 |
| 2.6 | 3269 | 0.34 | 1.0000 | 1 | 0.9977 | 0.0023 |
| 2.7 | 924 | 40.46 | 1.0000 | 2 | 0.0000 | 1.0000 |
| 2.7 | 1294 | 0.06 | 1.0000 | 1 | 0.9991 | 0.0009 |
| 2.8 | 131 | 0.27 | 1.0000 | 1 | 0.9984 | 0.0016 |
| 2.8 | 1007 | 4.43 | 1.0000 | 1 | 0.8540 | 0.1460 |
| 2.8 | 2023 | 0.16 | 1.0000 | 1 | 0.9991 | 0.0009 |
| 2.8 | 2620 | 0.05 | 1.0000 | 1 | 0.9987 | 0.0013 |
| 2.9 | 269 | 0.28 | 1.0000 | 1 | 0.9986 | 0.0014 |
| 2.9 | 467 | 0.17 | 1.0000 | 1 | 0.9988 | 0.0012 |
| 2.9 | 704 | 0.05 | 1.0000 | 1 | 0.9989 | 0.0011 |
| 2.9 | 791 | 0.26 | 1.0000 | 1 | 0.9990 | 0.0010 |
| 2.9 | 1605 | 0.19 | 1.0000 | 1 | 0.9992 | 0.0008 |
| 2.9 | 2477 | 0.24 | 1.0000 | 1 | 0.9988 | 0.0012 |
| 2.9 | 4247 | 11.51 | 1.0000 | 2 | 0.0000 | 1.0000 |
| 3 | 244 | 0.7 | 1.0000 | 1 | 0.9986 | 0.0014 |
| 3 | 1011 | 0.33 | 1.0000 | 1 | 0.9991 | 0.0009 |
| 3 | 1343 | 0.37 | 1.0000 | 1 | 0.9992 | 0.0008 |
| 3 | 1701 | 0.1 | 1.0000 | 1 | 0.9991 | 0.0009 |
| 3.1 | 402 | 0.05 | 1.0000 | 1 | 0.9986 | 0.0014 |
| 3.1 | 2794 | 0.05 | 1.0000 | 1 | 0.9984 | 0.0016 |
| 3.1 | 3956 | 0.05 | 1.0000 | 1 | 0.9931 | 0.0069 |
| 3.2 | 1023 | 0.27 | 1.0000 | 1 | 0.9991 | 0.0009 |
| 3.2 | 2511 | 0.26 | 1.0000 | 1 | 0.9988 | 0.0012 |
| 3.3 | 882 | 2.65 | 1.0000 | 1 | 0.9956 | 0.0044 |
| 3.3 | 2350 | 0.4 | 1.0000 | 1 | 0.9989 | 0.0011 |
| 3.3 | 3175 | 11.49 | 1.0000 | 2 | 0.0000 | 1.0000 |
| 3.4 | 1630 | 0.05 | 1.0000 | 1 | 0.9991 | 0.0009 |
| 3.4 | 2037 | 0.05 | 1.0000 | 1 | 0.9990 | 0.0010 |
| 3.4 | 8550 | 0.42 | 1.0000 | 2 | 0.0000 | 1.0000 |
| 3.5 | 2849 | 0.06 | 1.0000 | 1 | 0.9983 | 0.0017 |
| 3.5 | 3750 | 200 | 1.0000 | 2 | 0.0000 | 1.0000 |
| 3.5 | 4986 | 2.82 | 1.0000 | 1 | 0.7578 | 0.2422 |
| 3.6 | 316 | 0.18 | 1.0000 | 1 | 0.9986 | 0.0014 |
| 3.6 | 900 | 4.32 | 1.0000 | 1 | 0.8791 | 0.1209 |
| 3.6 | 1388 | 0.05 | 1.0000 | 1 | 0.9991 | 0.0009 |
| 3.6 | 1474 | 0.05 | 1.0000 | 1 | 0.9991 | 0.0009 |
| 3.6 | 2521 | 0.17 | 1.0000 | 1 | 0.9988 | 0.0012 |
| 3.7 | 162 | 34.37 | 1.0000 | 2 | 0.0000 | 1.0000 |
| 3.7 | 758 | 0.1 | 1.0000 | 1 | 0.9989 | 0.0011 |
| 3.7 | 1730 | 0.05 | 1.0000 | 1 | 0.9991 | 0.0009 |
| 3.7 | 2138 | 9.48 | 1.0000 | 2 | 0.0000 | 1.0000 |
| 3.8 | 349 | 0.22 | 1.0000 | 1 | 0.9986 | 0.0014 |
| 3.8 | 1340 | 0.85 | 1.0000 | 1 | 0.9991 | 0.0009 |
| 3.8 | 2222 | 0.08 | 1.0000 | 1 | 0.9989 | 0.0011 |
| 3.8 | 3344 | 0.21 | 1.0000 | 1 | 0.9972 | 0.0028 |
| 3.9 | 125 | 0.2 | 1.0000 | 1 | 0.9983 | 0.0017 |
| 3.9 | 199 | 0.15 | 1.0000 | 1 | 0.9984 | 0.0016 |
| 3.9 | 1254 | 0.12 | 1.0000 | 1 | 0.9991 | 0.0009 |
| 3.9 | 2318 | 0.05 | 1.0000 | 1 | 0.9988 | 0.0012 |
| 4.1 | 789 | 15.58 | 1.0000 | 2 | 0.0000 | 1.0000 |
| 4.1 | 816 | 0.05 | 1.0000 | 1 | 0.9989 | 0.0011 |
| 4.1 | 1410 | 0.05 | 1.0000 | 1 | 0.9991 | 0.0009 |
| 4.1 | 5150 | 17.69 | 1.0000 | 2 | 0.0000 | 1.0000 |
| 4.2 | 590 | 0.41 | 1.0000 | 1 | 0.9988 | 0.0012 |
| 4.2 | 2041 | 0.05 | 1.0000 | 1 | 0.9990 | 0.0010 |
| 4.2 | 4937 | 0.16 | 1.0000 | 1 | 0.9510 | 0.0490 |
| 4.3 | 956 | 0.05 | 1.0000 | 1 | 0.9990 | 0.0010 |
| 4.4 | 483 | 0.28 | 1.0000 | 1 | 0.9987 | 0.0013 |
| 4.4 | 950 | 8.2 | 1.0000 | 2 | 0.0000 | 1.0000 |
| 4.4 | 2120 | 1.36 | 1.0000 | 1 | 0.9988 | 0.0012 |
| 4.5 | 394 | 1.05 | 1.0000 | 1 | 0.9985 | 0.0015 |
| 4.5 | 3500 | 27.92 | 1.0000 | 2 | 0.0000 | 1.0000 |
| 4.5 | 3772 | 5.84 | 1.0000 | 2 | 0.0107 | 0.9893 |
| 4.5 | 22290 | 0.36 | 1.0000 | 2 | 0.0000 | 1.0000 |
| 4.6 | 366 | 0.29 | 1.0000 | 1 | 0.9986 | 0.0014 |
| 4.6 | 5180 | 0.14 | 1.0000 | 1 | 0.9127 | 0.0873 |
| 4.7 | 261 | 0.74 | 1.0000 | 1 | 0.9985 | 0.0015 |
| 4.7 | 674 | 0.05 | 1.0000 | 1 | 0.9988 | 0.0012 |
| 4.7 | 1258 | 0.59 | 1.0000 | 1 | 0.9991 | 0.0009 |
| 4.7 | 1262 | 0.13 | 1.0000 | 1 | 0.9990 | 0.0010 |
| 4.7 | 1747 | 0.6 | 1.0000 | 1 | 0.9991 | 0.0009 |
| 4.8 | 242 | 0.05 | 1.0000 | 1 | 0.9983 | 0.0017 |
| 4.8 | 1497 | 0.05 | 1.0000 | 1 | 0.9990 | 0.0010 |
| 4.8 | 5790 | 15.07 | 1.0000 | 2 | 0.0000 | 1.0000 |
| 4.9 | 1407 | 0.12 | 1.0000 | 1 | 0.9990 | 0.0010 |
| 4.9 | 1646 | 0.13 | 1.0000 | 1 | 0.9990 | 0.0010 |
| 4.9 | 1842 | 0.33 | 1.0000 | 1 | 0.9991 | 0.0009 |
| 5 | 485 | 0.27 | 1.0000 | 1 | 0.9987 | 0.0013 |
| 5 | 1255 | 0.08 | 1.0000 | 1 | 0.9990 | 0.0010 |
| 5 | 2054 | 0.05 | 1.0000 | 1 | 0.9989 | 0.0011 |
| 5 | 2854 | 3.92 | 1.0000 | 1 | 0.9189 | 0.0811 |
| 5.1 | 179 | 0.05 | 1.0000 | 1 | 0.9982 | 0.0018 |
| 5.1 | 1045 | 0.86 | 1.0000 | 1 | 0.9990 | 0.0010 |
| 5.1 | 1455 | 3.9 | 1.0000 | 1 | 0.9564 | 0.0436 |
| 5.1 | 1951 | 0.05 | 1.0000 | 1 | 0.9990 | 0.0010 |
| 5.1 | 2142 | 0.96 | 1.0000 | 1 | 0.9989 | 0.0011 |
| 5.1 | 4699 | 0.23 | 1.0000 | 1 | 0.9702 | 0.0298 |
| 5.1 | 7150 | 5.91 | 1.0000 | 2 | 0.0000 | 1.0000 |
| 5.2 | 907 | 0.51 | 1.0000 | 1 | 0.9990 | 0.0010 |
| 5.2 | 1210 | 0.05 | 1.0000 | 1 | 0.9990 | 0.0010 |
| 5.2 | 2367 | 0.05 | 1.0000 | 1 | 0.9987 | 0.0013 |
| 5.3 | 255 | 1.69 | 1.0000 | 1 | 0.9976 | 0.0024 |
| 5.3 | 1639 | 1.12 | 1.0000 | 1 | 0.9990 | 0.0010 |
| 5.3 | 1712 | 0.49 | 1.0000 | 1 | 0.9991 | 0.0009 |
| 5.3 | 3060 | 0.18 | 1.0000 | 1 | 0.9978 | 0.0022 |
| 5.3 | 3847 | 0.13 | 1.0000 | 1 | 0.9937 | 0.0063 |
| 5.3 | 10000 | 0.05 | 1.0000 | 2 | 0.0000 | 1.0000 |
| 5.4 | 1358 | 0.1 | 1.0000 | 1 | 0.9990 | 0.0010 |
| 5.4 | 1857 | 0.08 | 1.0000 | 1 | 0.9990 | 0.0010 |
| 5.4 | 2018 | 22.09 | 1.0000 | 2 | 0.0000 | 1.0000 |
| 5.5 | 25 | 0.05 | 1.0000 | 1 | 0.9979 | 0.0021 |
| 5.5 | 817 | 0.14 | 1.0000 | 1 | 0.9989 | 0.0011 |
| 5.5 | 959 | 0.2 | 1.0000 | 1 | 0.9989 | 0.0011 |
| 5.5 | 1090 | 0.05 | 1.0000 | 1 | 0.9989 | 0.0011 |
| 5.6 | 882 | 0.05 | 1.0000 | 1 | 0.9988 | 0.0012 |
| 5.6 | 1208 | 0.1 | 1.0000 | 1 | 0.9990 | 0.0010 |
| 5.7 | 573 | 0.12 | 1.0000 | 1 | 0.9987 | 0.0013 |
| 5.7 | 954 | 0.05 | 1.0000 | 1 | 0.9989 | 0.0011 |
| 5.7 | 2465 | 4.4 | 1.0000 | 1 | 0.8150 | 0.1850 |
| 5.8 | 927 | 0.51 | 1.0000 | 1 | 0.9990 | 0.0010 |
| 5.9 | 1348 | 0.05 | 1.0000 | 1 | 0.9990 | 0.0010 |
| 5.9 | 1420 | 0.06 | 1.0000 | 1 | 0.9990 | 0.0010 |
| 5.9 | 2136 | 0.41 | 1.0000 | 1 | 0.9989 | 0.0011 |
| 5.9 | 2145 | 5.1 | 1.0000 | 2 | 0.3971 | 0.6029 |
| 5.9 | 10000 | 5.07 | 1.0000 | 2 | 0.0000 | 1.0000 |
| 6 | 3154 | 4.18 | 1.0000 | 1 | 0.8067 | 0.1933 |
| 6 | 3182 | 0.05 | 1.0000 | 1 | 0.9973 | 0.0027 |
| 6.1 | 338 | 0.17 | 1.0000 | 1 | 0.9984 | 0.0016 |
| 6.1 | 14800 | 2.58 | 1.0000 | 2 | 0.0000 | 1.0000 |
| 6.2 | 172 | 0.11 | 1.0000 | 1 | 0.9981 | 0.0019 |
| 6.2 | 2268 | 0.1 | 1.0000 | 1 | 0.9988 | 0.0012 |
| 6.2 | 3224 | 15 | 1.0000 | 2 | 0.0000 | 1.0000 |
| 6.2 | 3774 | 0.43 | 1.0000 | 1 | 0.9945 | 0.0055 |
| 6.2 | 15160 | 2.27 | 1.0000 | 2 | 0.0000 | 1.0000 |
| 6.3 | 171 | 0.74 | 1.0000 | 1 | 0.9982 | 0.0018 |
| 6.3 | 947 | 0.17 | 1.0000 | 1 | 0.9989 | 0.0011 |
| 6.3 | 2276 | 0.5 | 1.0000 | 1 | 0.9988 | 0.0012 |
| 6.3 | 4638 | 0.05 | 1.0000 | 1 | 0.9706 | 0.0294 |
| 6.4 | 386 | 0.07 | 1.0000 | 1 | 0.9984 | 0.0016 |
| 6.4 | 1038 | 1.1 | 1.0000 | 1 | 0.9989 | 0.0011 |
| 6.5 | 121 | 0.35 | 1.0000 | 1 | 0.9981 | 0.0019 |
| 6.5 | 1464 | 0.83 | 1.0000 | 1 | 0.9990 | 0.0010 |
| 6.5 | 1769 | 0.44 | 1.0000 | 1 | 0.9990 | 0.0010 |
| 6.5 | 2719 | 0.05 | 1.0000 | 1 | 0.9983 | 0.0017 |
| 6.5 | 10000 | 29.02 | 1.0000 | 2 | 0.0000 | 1.0000 |
| 6.6 | 390 | 2.22 | 1.0000 | 1 | 0.9961 | 0.0039 |
| 6.6 | 1607 | 0.05 | 1.0000 | 1 | 0.9989 | 0.0011 |
| 6.6 | 1961 | 0.06 | 1.0000 | 1 | 0.9989 | 0.0011 |
| 6.6 | 5970 | 0.38 | 1.0000 | 1 | 0.5205 | 0.4795 |
| 6.7 | 1167 | 0.05 | 1.0000 | 1 | 0.9989 | 0.0011 |
| 6.7 | 1698 | 0.14 | 1.0000 | 1 | 0.9989 | 0.0011 |
| 6.7 | 2988 | 0.14 | 1.0000 | 1 | 0.9978 | 0.0022 |
| 6.7 | 3074 | 0.25 | 1.0000 | 1 | 0.9977 | 0.0023 |
| 6.8 | 1265 | 0.39 | 1.0000 | 1 | 0.9990 | 0.0010 |
| 6.8 | 4053 | 0.36 | 1.0000 | 1 | 0.9909 | 0.0091 |
| 6.9 | 547 | 2.56 | 1.0000 | 1 | 0.9945 | 0.0055 |
| 6.9 | 1190 | 0.19 | 1.0000 | 1 | 0.9989 | 0.0011 |
| 6.9 | 1829 | 0.05 | 1.0000 | 1 | 0.9989 | 0.0011 |
| 6.9 | 3737 | 2.11 | 1.0000 | 1 | 0.9875 | 0.0125 |
| 7 | 378 | 0.05 | 1.0000 | 1 | 0.9983 | 0.0017 |
| 7 | 810 | 0.05 | 1.0000 | 1 | 0.9987 | 0.0013 |
| 7 | 1524 | 0.04 | 1.0000 | 1 | 0.9989 | 0.0011 |
| 7.1 | 1048 | 0.32 | 1.0000 | 1 | 0.9989 | 0.0011 |
| 7.1 | 1433 | 0.42 | 1.0000 | 1 | 0.9990 | 0.0010 |
| 7.2 | 739 | 0.09 | 1.0000 | 1 | 0.9987 | 0.0013 |
| 7.2 | 783 | 0.05 | 1.0000 | 1 | 0.9987 | 0.0013 |
| 7.2 | 3855 | 2.9 | 1.0000 | 1 | 0.9563 | 0.0437 |
| 7.3 | 702 | 0.05 | 1.0000 | 1 | 0.9986 | 0.0014 |
| 7.3 | 2069 | 0.61 | 1.0000 | 1 | 0.9989 | 0.0011 |
| 7.4 | 1412 | 0.05 | 1.0000 | 1 | 0.9989 | 0.0011 |
| 7.4 | 7774 | 0.05 | 1.0000 | 2 | 0.0012 | 0.9988 |
| 7.4 | 10000 | 0.12 | 1.0000 | 2 | 0.0000 | 1.0000 |
| 7.5 | 457 | 0.05 | 1.0000 | 1 | 0.9984 | 0.0016 |
| 7.5 | 1773 | 0.21 | 1.0000 | 1 | 0.9989 | 0.0011 |
| 7.5 | 5018 | 0.05 | 1.0000 | 1 | 0.9284 | 0.0716 |
| 7.5 | 5880 | 0.87 | 1.0000 | 1 | 0.5700 | 0.4300 |
| 7.6 | 101 | 0.05 | 1.0000 | 1 | 0.9978 | 0.0022 |
| 7.6 | 291 | 0.28 | 1.0000 | 1 | 0.9982 | 0.0018 |
| 7.6 | 2092 | 6.62 | 1.0000 | 2 | 0.0021 | 0.9979 |
| 7.7 | 1235 | 31.96 | 1.0000 | 2 | 0.0000 | 1.0000 |
| 7.7 | 2173 | 0.23 | 1.0000 | 1 | 0.9988 | 0.0012 |
| 7.7 | 2402 | 0.35 | 1.0000 | 1 | 0.9986 | 0.0014 |
| 7.7 | 2985 | 0.05 | 1.0000 | 1 | 0.9976 | 0.0024 |
| 7.7 | 10000 | 0.08 | 1.0000 | 2 | 0.0000 | 1.0000 |
| 7.8 | 1313 | 29.95 | 1.0000 | 2 | 0.0000 | 1.0000 |
| 7.8 | 1323 | 56.71 | 1.0000 | 2 | 0.0000 | 1.0000 |
| 7.8 | 1562 | 0.06 | 1.0000 | 1 | 0.9989 | 0.0011 |
| 7.8 | 2679 | 5.74 | 1.0000 | 2 | 0.0455 | 0.9545 |
| 7.9 | 440 | 0.2 | 1.0000 | 1 | 0.9984 | 0.0016 |
| 7.9 | 3510 | 0.48 | 1.0000 | 1 | 0.9959 | 0.0041 |
| 7.9 | 4038 | 0.14 | 1.0000 | 1 | 0.9901 | 0.0099 |
| 8 | 1311 | 0.29 | 1.0000 | 1 | 0.9989 | 0.0011 |
| 8.1 | 1070 | 0.53 | 1.0000 | 1 | 0.9989 | 0.0011 |
| 8.1 | 2124 | 2.24 | 1.0000 | 1 | 0.9968 | 0.0032 |
| 8.2 | 215 | 0.14 | 1.0000 | 1 | 0.9980 | 0.0020 |
| 8.2 | 1671 | 0.12 | 1.0000 | 1 | 0.9989 | 0.0011 |
| 8.2 | 3908 | 2.83 | 1.0000 | 1 | 0.9549 | 0.0451 |
| 8.3 | 981 | 43.49 | 1.0000 | 2 | 0.0000 | 1.0000 |
| 8.3 | 1663 | 57.58 | 1.0000 | 2 | 0.0000 | 1.0000 |
| 8.3 | 1768 | 1.97 | 1.0000 | 1 | 0.9978 | 0.0022 |
| 8.3 | 4616 | 142.4 | 1.0000 | 2 | 0.0000 | 1.0000 |
| 8.4 | 655 | 0.43 | 1.0000 | 1 | 0.9986 | 0.0014 |
| 8.4 | 958 | 140.2 | 1.0000 | 2 | 0.0000 | 1.0000 |
| 8.4 | 2389 | 113.6 | 1.0000 | 2 | 0.0000 | 1.0000 |
| 8.4 | 2538 | 0.85 | 1.0000 | 1 | 0.9984 | 0.0016 |
| 8.4 | 2810 | 3.4 | 1.0000 | 1 | 0.9681 | 0.0319 |
| 8.5 | 3976 | 10.91 | 1.0000 | 2 | 0.0000 | 1.0000 |
| 8.6 | 239 | 0.65 | 1.0000 | 1 | 0.9981 | 0.0019 |
| 8.6 | 1500 | 0.05 | 1.0000 | 1 | 0.9988 | 0.0012 |
| 8.7 | 903 | 0.25 | 1.0000 | 1 | 0.9987 | 0.0013 |
| 8.9 | 1380 | 0.05 | 1.0000 | 1 | 0.9988 | 0.0012 |
| 8.9 | 2205 | 0.05 | 1.0000 | 1 | 0.9986 | 0.0014 |
| 9 | 1860 | 0.05 | 1.0000 | 1 | 0.9987 | 0.0013 |
| 9 | 2569 | 0.15 | 1.0000 | 1 | 0.9983 | 0.0017 |
| 9.1 | 988 | 0.1 | 1.0000 | 1 | 0.9987 | 0.0013 |
| 9.1 | 8325 | 18.7 | 1.0000 | 2 | 0.0000 | 1.0000 |
| 9.2 | 1301 | 31.75 | 1.0000 | 2 | 0.0000 | 1.0000 |
| 9.2 | 1537 | 0.09 | 1.0000 | 1 | 0.9988 | 0.0012 |
| 9.2 | 2642 | 9.1 | 1.0000 | 2 | 0.0000 | 1.0000 |
| 9.2 | 2920 | 13.05 | 1.0000 | 2 | 0.0000 | 1.0000 |
| 9.3 | 2627 | 1.26 | 1.0000 | 1 | 0.9979 | 0.0021 |
| 9.3 | 5490 | 0.16 | 1.0000 | 1 | 0.7811 | 0.2189 |
| 9.4 | 851 | 1.46 | 1.0000 | 1 | 0.9982 | 0.0018 |
| 9.4 | 1525 | 7.15 | 1.0000 | 2 | 0.0002 | 0.9998 |
| 9.6 | 704 | 3.2 | 1.0000 | 1 | 0.9834 | 0.0166 |
| 9.6 | 1243 | 0.99 | 1.0000 | 1 | 0.9987 | 0.0013 |
| 9.6 | 1381 | 2.14 | 1.0000 | 1 | 0.9972 | 0.0028 |
| 9.6 | 17875 | 199.3 | 1.0000 | 2 | 0.0000 | 1.0000 |
| 9.7 | 1073 | 0.13 | 1.0000 | 1 | 0.9987 | 0.0013 |
| 9.9 | 1387 | 3.79 | 1.0000 | 1 | 0.9552 | 0.0448 |
| 10.1 | 784 | 0.96 | 1.0000 | 1 | 0.9985 | 0.0015 |
| 10.2 | 1141 | 0.06 | 1.0000 | 1 | 0.9986 | 0.0014 |
| 10.3 | 295 | 0.06 | 1.0000 | 1 | 0.9978 | 0.0022 |
| 10.3 | 331 | 0.15 | 1.0000 | 1 | 0.9979 | 0.0021 |
| 10.3 | 699 | 0.2 | 1.0000 | 1 | 0.9984 | 0.0016 |
| 10.3 | 2545 | 2.86 | 1.0000 | 1 | 0.9888 | 0.0112 |
| 10.4 | 870 | 0.33 | 1.0000 | 1 | 0.9986 | 0.0014 |
| 10.4 | 20000 | 0.53 | 1.0000 | 2 | 0.0000 | 1.0000 |
| 10.5 | 848 | 0.05 | 1.0000 | 1 | 0.9984 | 0.0016 |
| 10.5 | 1204 | 0.37 | 1.0000 | 1 | 0.9987 | 0.0013 |
| 10.5 | 1230 | 0.05 | 1.0000 | 1 | 0.9986 | 0.0014 |
| 10.5 | 2893 | 0.21 | 1.0000 | 1 | 0.9975 | 0.0025 |
| 10.6 | 979 | 0.05 | 1.0000 | 1 | 0.9985 | 0.0015 |
| 10.6 | 1929 | 0.75 | 1.0000 | 1 | 0.9987 | 0.0013 |
| 10.6 | 3210 | 11.9 | 1.0000 | 2 | 0.0000 | 1.0000 |
| 10.7 | 3122 | 20.35 | 1.0000 | 2 | 0.0000 | 1.0000 |
| 10.8 | 417 | 0.05 | 1.0000 | 1 | 0.9979 | 0.0021 |
| 10.8 | 3810 | 0.43 | 1.0000 | 1 | 0.9923 | 0.0077 |
| 10.9 | 1280 | 0.18 | 1.0000 | 1 | 0.9986 | 0.0014 |
| 10.9 | 2497 | 0.33 | 1.0000 | 1 | 0.9982 | 0.0018 |
| 11 | 250 | 0.67 | 1.0000 | 1 | 0.9978 | 0.0022 |
| 11.1 | 2532 | 10.14 | 1.0000 | 2 | 0.0000 | 1.0000 |
| 11.1 | 3190 | 0.19 | 1.0000 | 1 | 0.9965 | 0.0035 |
| 11.2 | 4482 | 0.07 | 1.0000 | 1 | 0.9716 | 0.0284 |
| 11.3 | 803 | 0.05 | 1.0000 | 1 | 0.9983 | 0.0017 |
| 11.4 | 912 | 0.05 | 1.0000 | 1 | 0.9984 | 0.0016 |
| 11.4 | 2305 | 19.17 | 1.0000 | 2 | 0.0000 | 1.0000 |
| 11.4 | 12480 | 0.09 | 1.0000 | 2 | 0.0000 | 1.0000 |
| 11.5 | 1196 | 3.04 | 1.0000 | 1 | 0.9882 | 0.0118 |
| 11.5 | 1319 | 0.05 | 1.0000 | 1 | 0.9985 | 0.0015 |
| 11.6 | 4579 | 6.93 | 1.0000 | 2 | 0.0000 | 1.0000 |
| 11.7 | 1003 | 2.63 | 1.0000 | 1 | 0.9934 | 0.0066 |
| 11.7 | 1443 | 118.7 | 1.0000 | 2 | 0.0000 | 1.0000 |
| 11.7 | 2912 | 0.29 | 1.0000 | 1 | 0.9973 | 0.0027 |
| 11.8 | 2205 | 24.3 | 1.0000 | 2 | 0.0000 | 1.0000 |
| 11.9 | 1130 | 0.05 | 1.0000 | 1 | 0.9984 | 0.0016 |
| 11.9 | 3369 | 7.68 | 1.0000 | 2 | 0.0000 | 1.0000 |
| 12 | 497 | 3.67 | 1.0000 | 1 | 0.9446 | 0.0554 |
| 12 | 3504 | 39.56 | 1.0000 | 2 | 0.0000 | 1.0000 |
| 12.1 | 1083 | 0.18 | 1.0000 | 1 | 0.9985 | 0.0015 |
| 12.1 | 2047 | 0.24 | 1.0000 | 1 | 0.9984 | 0.0016 |
| 12.2 | 4346 | 11.93 | 1.0000 | 2 | 0.0000 | 1.0000 |
| 12.2 | 4732 | 0.07 | 1.0000 | 1 | 0.9493 | 0.0507 |
| 12.3 | 1848 | 0.12 | 1.0000 | 1 | 0.9985 | 0.0015 |
| 12.5 | 755 | 0.15 | 1.0000 | 1 | 0.9982 | 0.0018 |
| 12.5 | 759 | 0.51 | 1.0000 | 1 | 0.9983 | 0.0017 |
| 12.5 | 1340 | 0.05 | 1.0000 | 1 | 0.9984 | 0.0016 |
| 12.5 | 2237 | 0.09 | 1.0000 | 1 | 0.9982 | 0.0018 |
| 12.5 | 3416 | 0.05 | 1.0000 | 1 | 0.9946 | 0.0054 |
| 12.5 | 7430 | 9.47 | 1.0000 | 2 | 0.0000 | 1.0000 |
| 12.6 | 31 | 0.66 | 1.0000 | 1 | 0.9970 | 0.0030 |
| 12.6 | 851 | 0.68 | 1.0000 | 1 | 0.9983 | 0.0017 |
| 12.7 | 1701 | 0.54 | 1.0000 | 1 | 0.9985 | 0.0015 |
| 12.8 | 1053 | 1.17 | 1.0000 | 1 | 0.9982 | 0.0018 |
| 12.8 | 3730 | 0.44 | 1.0000 | 1 | 0.9922 | 0.0078 |
| 12.9 | 190 | 0.55 | 1.0000 | 1 | 0.9973 | 0.0027 |
| 12.9 | 1816 | 0.09 | 1.0000 | 1 | 0.9984 | 0.0016 |
| 13 | 879 | 0.05 | 1.0000 | 1 | 0.9982 | 0.0018 |
| 13 | 1229 | 0.3 | 1.0000 | 1 | 0.9985 | 0.0015 |
| 13.1 | 1088 | 0.15 | 1.0000 | 1 | 0.9983 | 0.0017 |
| 13.1 | 1246 | 0.38 | 1.0000 | 1 | 0.9985 | 0.0015 |
| 13.1 | 1600 | 0.28 | 1.0000 | 1 | 0.9985 | 0.0015 |
| 13.1 | 2070 | 0.12 | 1.0000 | 1 | 0.9982 | 0.0018 |
| 13.2 | 788 | 25.91 | 1.0000 | 2 | 0.0000 | 1.0000 |
| 13.2 | 3843 | 0.33 | 1.0000 | 1 | 0.9903 | 0.0097 |
| 13.3 | 2833 | 0.05 | 1.0000 | 1 | 0.9970 | 0.0030 |
| 13.4 | 501 | 0.09 | 1.0000 | 1 | 0.9977 | 0.0023 |
| 13.6 | 1142 | 0.06 | 1.0000 | 1 | 0.9982 | 0.0018 |
| 13.6 | 1350 | 0.86 | 1.0000 | 1 | 0.9984 | 0.0016 |
| 13.6 | 1980 | 0.13 | 1.0000 | 1 | 0.9982 | 0.0018 |
| 13.8 | 427 | 0.2 | 1.0000 | 1 | 0.9976 | 0.0024 |
| 13.8 | 4830 | 0.12 | 1.0000 | 1 | 0.9313 | 0.0687 |
| 14.1 | 4915 | 7.57 | 1.0000 | 2 | 0.0000 | 1.0000 |
| 14.2 | 1216 | 11.06 | 1.0000 | 2 | 0.0000 | 1.0000 |
| 14.2 | 2675 | 0.68 | 1.0000 | 1 | 0.9974 | 0.0026 |
| 14.3 | 1721 | 1.47 | 1.0000 | 1 | 0.9978 | 0.0022 |
| 14.4 | 974 | 0.2 | 1.0000 | 1 | 0.9981 | 0.0019 |
| 14.4 | 1421 | 0.19 | 1.0000 | 1 | 0.9983 | 0.0017 |
| 14.5 | 808 | 0.14 | 1.0000 | 1 | 0.9979 | 0.0021 |
| 14.5 | 1944 | 0.81 | 1.0000 | 1 | 0.9982 | 0.0018 |
| 14.6 | 1034 | 0.05 | 1.0000 | 1 | 0.9980 | 0.0020 |
| 14.6 | 3350 | 0.51 | 1.0000 | 1 | 0.9947 | 0.0053 |
| 14.7 | 270 | 0.98 | 1.0000 | 1 | 0.9970 | 0.0030 |
| 14.7 | 844 | 80 | 1.0000 | 2 | 0.0000 | 1.0000 |
| 14.7 | 1484 | 1.52 | 1.0000 | 1 | 0.9977 | 0.0023 |
| 14.7 | 2464 | 0.51 | 1.0000 | 1 | 0.9977 | 0.0023 |
| 14.8 | 271 | 26.81 | 1.0000 | 2 | 0.0000 | 1.0000 |
| 14.9 | 337 | 0.33 | 1.0000 | 1 | 0.9972 | 0.0028 |
| 14.9 | 1039 | 0.82 | 1.0000 | 1 | 0.9981 | 0.0019 |
| 14.9 | 2114 | 0.29 | 1.0000 | 1 | 0.9980 | 0.0020 |
| 15 | 1550 | 0.96 | 1.0000 | 1 | 0.9982 | 0.0018 |
| 15.1 | 751 | 16.41 | 1.0000 | 2 | 0.0000 | 1.0000 |
| 15.1 | 1814 | 0.14 | 1.0000 | 1 | 0.9981 | 0.0019 |
| 15.1 | 2117 | 0.43 | 1.0000 | 1 | 0.9980 | 0.0020 |
| 15.2 | 1106 | 1.87 | 1.0000 | 1 | 0.9966 | 0.0034 |
| 15.2 | 1256 | 0.17 | 1.0000 | 1 | 0.9981 | 0.0019 |
| 15.3 | 779 | 3.53 | 1.0000 | 1 | 0.9551 | 0.0449 |
| 15.3 | 1975 | 12.61 | 1.0000 | 2 | 0.0000 | 1.0000 |
| 15.3 | 2560 | 1.58 | 1.0000 | 1 | 0.9963 | 0.0037 |
| 15.3 | 2709 | 0.28 | 1.0000 | 1 | 0.9971 | 0.0029 |
| 15.5 | 2013 | 2.53 | 1.0000 | 1 | 0.9925 | 0.0075 |
| 15.5 | 2054 | 0.31 | 1.0000 | 1 | 0.9980 | 0.0020 |
| 15.6 | 3606 | 0.25 | 1.0000 | 1 | 0.9917 | 0.0083 |
| 15.7 | 494 | 0.28 | 1.0000 | 1 | 0.9974 | 0.0026 |
| 15.7 | 944 | 0.66 | 1.0000 | 1 | 0.9980 | 0.0020 |
| 15.7 | 1283 | 0.05 | 1.0000 | 1 | 0.9980 | 0.0020 |
| 15.7 | 1650 | 18.61 | 1.0000 | 2 | 0.0000 | 1.0000 |
| 15.9 | 2911 | 2.25 | 1.0000 | 1 | 0.9902 | 0.0098 |
| 15.9 | 3338 | 0.33 | 1.0000 | 1 | 0.9941 | 0.0059 |
| 16 | 1623 | 0.13 | 1.0000 | 1 | 0.9980 | 0.0020 |
| 16.1 | 514 | 0.41 | 1.0000 | 1 | 0.9974 | 0.0026 |
| 16.1 | 2078 | 0.32 | 1.0000 | 1 | 0.9979 | 0.0021 |
| 16.1 | 9310 | 5.39 | 1.0000 | 2 | 0.0000 | 1.0000 |
| 16.2 | 2905 | 200 | 1.0000 | 2 | 0.0000 | 1.0000 |
| 16.3 | 426 | 6.68 | 1.0000 | 2 | 0.0007 | 0.9993 |
| 16.4 | 491 | 0.16 | 1.0000 | 1 | 0.9971 | 0.0029 |
| 16.4 | 1379 | 0.08 | 1.0000 | 1 | 0.9979 | 0.0021 |
| 16.4 | 33440 | 0.21 | 1.0000 | 2 | 0.0000 | 1.0000 |
| 16.6 | 1194 | 7.39 | 1.0000 | 2 | 0.0000 | 1.0000 |
| 16.6 | 4595 | 14.86 | 1.0000 | 2 | 0.0000 | 1.0000 |
| 16.7 | 1308 | 0.05 | 1.0000 | 1 | 0.9978 | 0.0022 |
| 16.7 | 1382 | 44.14 | 1.0000 | 2 | 0.0000 | 1.0000 |
| 16.7 | 1702 | 0.06 | 1.0000 | 1 | 0.9978 | 0.0022 |
| 16.7 | 4326 | 10.36 | 1.0000 | 2 | 0.0000 | 1.0000 |
| 16.8 | 1623 | 0.05 | 1.0000 | 1 | 0.9978 | 0.0022 |
| 16.8 | 2284 | 20.1 | 1.0000 | 2 | 0.0000 | 1.0000 |
| 16.8 | 3000 | 0.4 | 1.0000 | 1 | 0.9957 | 0.0043 |
| 16.9 | 444 | 0.56 | 1.0000 | 1 | 0.9971 | 0.0029 |
| 17.1 | 702 | 0.19 | 1.0000 | 1 | 0.9973 | 0.0027 |
| 17.1 | 2988 | 15.09 | 1.0000 | 2 | 0.0000 | 1.0000 |
| 17.2 | 1028 | 0.23 | 1.0000 | 1 | 0.9977 | 0.0023 |
| 17.2 | 1739 | 0.79 | 1.0000 | 1 | 0.9979 | 0.0021 |
| 17.2 | 2918 | 0.08 | 1.0000 | 1 | 0.9956 | 0.0044 |
| 17.2 | 2997 | 0.25 | 1.0000 | 1 | 0.9955 | 0.0045 |
| 17.3 | 1427 | 0.25 | 1.0000 | 1 | 0.9979 | 0.0021 |
| 17.4 | 1835 | 0.58 | 1.0000 | 1 | 0.9978 | 0.0022 |
| 17.4 | 4236 | 0.5 | 1.0000 | 1 | 0.9739 | 0.0261 |
| 17.7 | 603 | 0.42 | 1.0000 | 1 | 0.9971 | 0.0029 |
| 17.7 | 1141 | 1.99 | 1.0000 | 1 | 0.9954 | 0.0046 |
| 17.7 | 4812 | 3.35 | 1.0000 | 2 | 0.4154 | 0.5846 |
| 17.8 | 2050 | 0.49 | 1.0000 | 1 | 0.9976 | 0.0024 |
| 18 | 327 | 0.26 | 1.0000 | 1 | 0.9963 | 0.0037 |
| 18 | 2055 | 60.68 | 1.0000 | 2 | 0.0000 | 1.0000 |
| 18.1 | 1697 | 51.24 | 1.0000 | 2 | 0.0000 | 1.0000 |
| 18.3 | 4573 | 0.69 | 1.0000 | 1 | 0.9460 | 0.0540 |
| 18.3 | 6800 | 19.56 | 1.0000 | 2 | 0.0000 | 1.0000 |
| 18.4 | 1136 | 1.45 | 1.0000 | 1 | 0.9969 | 0.0031 |
| 18.4 | 2636 | 0.29 | 1.0000 | 1 | 0.9964 | 0.0036 |
| 18.5 | 1764 | 17.23 | 1.0000 | 2 | 0.0000 | 1.0000 |
| 18.6 | 311 | 1.89 | 1.0000 | 1 | 0.9930 | 0.0070 |
| 18.6 | 2269 | 0.23 | 1.0000 | 1 | 0.9971 | 0.0029 |
| 18.7 | 2334 | 11.71 | 1.0000 | 2 | 0.0000 | 1.0000 |
| 18.7 | 2606 | 4.69 | 1.0000 | 2 | 0.4121 | 0.5879 |
| 18.8 | 1547 | 5.6 | 1.0000 | 2 | 0.0527 | 0.9473 |
| 19 | 1171 | 0.42 | 1.0000 | 1 | 0.9975 | 0.0025 |
| 19.2 | 996 | 0.05 | 1.0000 | 1 | 0.9971 | 0.0029 |
| 19.2 | 1007 | 5.76 | 1.0000 | 2 | 0.0269 | 0.9731 |
| 19.3 | 20000 | 28.92 | 1.0000 | 2 | 0.0000 | 1.0000 |
| 19.4 | 3517 | 11.43 | 1.0000 | 2 | 0.0000 | 1.0000 |
| 19.4 | 5139 | 21.97 | 1.0000 | 2 | 0.0000 | 1.0000 |
| 19.6 | 2936 | 20.09 | 1.0000 | 2 | 0.0000 | 1.0000 |
| 19.7 | 1779 | 0.59 | 1.0000 | 1 | 0.9974 | 0.0026 |
| 19.8 | 964 | 0.3 | 1.0000 | 1 | 0.9971 | 0.0029 |
| 19.8 | 1400 | 0.47 | 1.0000 | 1 | 0.9974 | 0.0026 |
| 19.8 | 1504 | 3.49 | 1.0000 | 1 | 0.9497 | 0.0503 |
| 19.8 | 3632 | 0.24 | 1.0000 | 1 | 0.9876 | 0.0124 |
| 19.8 | 5120 | 3.68 | 1.0000 | 2 | 0.1246 | 0.8754 |
| 19.9 | 1170 | 3.1 | 1.0000 | 1 | 0.9744 | 0.0256 |
| 20 | 829 | 5.63 | 1.0000 | 2 | 0.0367 | 0.9633 |
| 20 | 3707 | 31.48 | 1.0000 | 2 | 0.0000 | 1.0000 |
| 20.1 | 933 | 7.74 | 1.0000 | 2 | 0.0000 | 1.0000 |
| 20.3 | 3138 | 0.23 | 1.0000 | 1 | 0.9931 | 0.0069 |
| 20.4 | 1193 | 0.1 | 1.0000 | 1 | 0.9970 | 0.0030 |
| 20.4 | 3670 | 0.05 | 1.0000 | 1 | 0.9854 | 0.0146 |
| 20.4 | 6500 | 5.2 | 1.0000 | 2 | 0.0000 | 1.0000 |
| 20.7 | 1114 | 5.24 | 1.0000 | 2 | 0.1315 | 0.8685 |
| 20.8 | 700 | 0.23 | 1.0000 | 1 | 0.9963 | 0.0037 |
| 20.8 | 1504 | 0.28 | 1.0000 | 1 | 0.9971 | 0.0029 |
| 20.8 | 2142 | 6.52 | 1.0000 | 2 | 0.0011 | 0.9989 |
| 21 | 2990 | 0.51 | 1.0000 | 1 | 0.9939 | 0.0061 |
| 21.2 | 1014 | 2.82 | 1.0000 | 1 | 0.9810 | 0.0190 |
| 21.2 | 2180 | 12.81 | 1.0000 | 2 | 0.0000 | 1.0000 |
| 21.2 | 3173 | 53.78 | 1.0000 | 2 | 0.0000 | 1.0000 |
| 21.3 | 4440 | 75.54 | 1.0000 | 2 | 0.0000 | 1.0000 |
| 21.4 | 984 | 0.07 | 1.0000 | 1 | 0.9964 | 0.0036 |
| 21.5 | 396 | 0.11 | 1.0000 | 1 | 0.9950 | 0.0050 |
| 21.5 | 1231 | 0.32 | 1.0000 | 1 | 0.9968 | 0.0032 |
| 21.6 | 737 | 0.11 | 1.0000 | 1 | 0.9960 | 0.0040 |
| 21.6 | 1160 | 0.5 | 1.0000 | 1 | 0.9968 | 0.0032 |
| 21.6 | 6910 | 21.97 | 1.0000 | 2 | 0.0000 | 1.0000 |
| 21.7 | 1091 | 21.75 | 1.0000 | 2 | 0.0000 | 1.0000 |
| 21.7 | 2368 | 0.22 | 1.0000 | 1 | 0.9959 | 0.0041 |
| 21.8 | 8350 | 106.4 | 1.0000 | 2 | 0.0000 | 1.0000 |
| 21.9 | 1562 | 12.36 | 1.0000 | 2 | 0.0000 | 1.0000 |
| 22 | 3406 | 0.62 | 1.0000 | 1 | 0.9892 | 0.0108 |
| 22.1 | 1227 | 4.49 | 1.0000 | 1 | 0.5683 | 0.4317 |
| 22.1 | 3890 | 12.87 | 1.0000 | 2 | 0.0000 | 1.0000 |
| 22.3 | 1104 | 1.02 | 1.0000 | 1 | 0.9963 | 0.0037 |
| 22.4 | 605 | 0.25 | 1.0000 | 1 | 0.9955 | 0.0045 |
| 22.4 | 3222 | 9.08 | 1.0000 | 2 | 0.0000 | 1.0000 |
| 22.5 | 581 | 0.57 | 1.0000 | 1 | 0.9955 | 0.0045 |
| 22.6 | 1394 | 3.03 | 1.0000 | 1 | 0.9717 | 0.0283 |
| 22.6 | 3356 | 0.73 | 1.0000 | 1 | 0.9891 | 0.0109 |
| 22.9 | 1229 | 0.99 | 1.0000 | 1 | 0.9962 | 0.0038 |
| 22.9 | 2419 | 0.22 | 1.0000 | 1 | 0.9952 | 0.0048 |
| 22.9 | 2909 | 3.59 | 1.0000 | 1 | 0.8527 | 0.1473 |
| 22.9 | 10000 | 20 | 1.0000 | 2 | 0.0000 | 1.0000 |
| 23 | 1653 | 0.96 | 1.0000 | 1 | 0.9963 | 0.0037 |
| 23.1 | 993 | 1.22 | 1.0000 | 1 | 0.9955 | 0.0045 |
| 23.1 | 1475 | 8.37 | 1.0000 | 2 | 0.0000 | 1.0000 |
| 23.1 | 1955 | 79.35 | 1.0000 | 2 | 0.0000 | 1.0000 |
| 23.2 | 4092 | 7.09 | 1.0000 | 2 | 0.0000 | 1.0000 |
| 23.3 | 488 | 26.99 | 1.0000 | 2 | 0.0000 | 1.0000 |
| 23.3 | 2135 | 0.86 | 1.0000 | 1 | 0.9957 | 0.0043 |
| 23.4 | 1176 | 0.6 | 1.0000 | 1 | 0.9962 | 0.0038 |
| 23.4 | 1312 | 120.4 | 1.0000 | 2 | 0.0000 | 1.0000 |
| 23.4 | 1802 | 0.44 | 1.0000 | 1 | 0.9962 | 0.0038 |
| 23.4 | 4819 | 0.35 | 1.0000 | 1 | 0.8615 | 0.1385 |
| 23.5 | 294 | 0.08 | 1.0000 | 1 | 0.9933 | 0.0067 |
| 23.5 | 3427 | 0.36 | 1.0000 | 1 | 0.9869 | 0.0131 |
| 23.6 | 966 | 29.77 | 1.0000 | 2 | 0.0000 | 1.0000 |
| 23.6 | 2680 | 0.59 | 1.0000 | 1 | 0.9940 | 0.0060 |
| 23.7 | 1230 | 0.51 | 1.0000 | 1 | 0.9961 | 0.0039 |
| 23.8 | 1137 | 1.18 | 1.0000 | 1 | 0.9954 | 0.0046 |
| 24 | 1698 | 53.11 | 1.0000 | 2 | 0.0000 | 1.0000 |
| 24 | 5560 | 0.17 | 1.0000 | 2 | 0.4627 | 0.5373 |
| 24.1 | 2573 | 5.87 | 1.0000 | 2 | 0.0084 | 0.9916 |
| 24.1 | 2605 | 0.26 | 1.0000 | 1 | 0.9939 | 0.0061 |
| 24.4 | 790 | 200 | 1.0000 | 2 | 0.0000 | 1.0000 |
| 24.4 | 3748 | 4.54 | 1.0000 | 2 | 0.1427 | 0.8573 |
| 24.5 | 826 | 2.71 | 1.0000 | 1 | 0.9759 | 0.0241 |
| 24.6 | 4634 | 27.65 | 1.0000 | 2 | 0.0000 | 1.0000 |
| 24.6 | 10000 | 200 | 1.0000 | 2 | 0.0000 | 1.0000 |
| 24.9 | 2700 | 42.34 | 1.0000 | 2 | 0.0000 | 1.0000 |
| 24.9 | 3922 | 0.09 | 1.0000 | 1 | 0.9669 | 0.0331 |
| 24.9 | 4316 | 11.03 | 1.0000 | 2 | 0.0000 | 1.0000 |
| 24.9 | 6570 | 3.69 | 1.0000 | 2 | 0.0011 | 0.9989 |
| 24.9 | 9510 | 0.32 | 1.0000 | 2 | 0.0000 | 1.0000 |
| 25 | 708 | 20.12 | 1.0000 | 2 | 0.0000 | 1.0000 |
| 25 | 934 | 25.01 | 1.0000 | 2 | 0.0000 | 1.0000 |
| 25 | 1830 | 0.31 | 1.0000 | 1 | 0.9954 | 0.0046 |
| 25 | 6110 | 18.08 | 1.0000 | 2 | 0.0000 | 1.0000 |
| 25.1 | 2619 | 10.24 | 1.0000 | 2 | 0.0000 | 1.0000 |
| 25.2 | 1364 | 13.71 | 1.0000 | 2 | 0.0000 | 1.0000 |
| 25.2 | 3468 | 1.23 | 1.0000 | 1 | 0.9810 | 0.0190 |
| 25.3 | 1717 | 0.88 | 1.0000 | 1 | 0.9953 | 0.0047 |
| 25.4 | 2320 | 20.79 | 1.0000 | 2 | 0.0000 | 1.0000 |
| 25.5 | 4420 | 0.45 | 1.0000 | 1 | 0.9221 | 0.0779 |
| 25.6 | 1027 | 8.72 | 1.0000 | 2 | 0.0000 | 1.0000 |
| 25.9 | 5950 | 1.75 | 1.0000 | 2 | 0.1258 | 0.8742 |
| 26.1 | 47 | 0.18 | 1.0000 | 1 | 0.9893 | 0.0107 |
| 26.1 | 2883 | 25.47 | 1.0000 | 2 | 0.0000 | 1.0000 |
| 26.3 | 731 | 0.98 | 1.0000 | 1 | 0.9935 | 0.0065 |
| 26.6 | 1076 | 0.98 | 1.0000 | 1 | 0.9942 | 0.0058 |
| 26.6 | 3115 | 35.42 | 1.0000 | 2 | 0.0000 | 1.0000 |
| 26.7 | 1167 | 34.45 | 1.0000 | 2 | 0.0000 | 1.0000 |
| 26.8 | 647 | 7.56 | 1.0000 | 2 | 0.0000 | 1.0000 |
| 27 | 793 | 1.13 | 1.0000 | 1 | 0.9928 | 0.0072 |
| 27 | 3225 | 21.09 | 1.0000 | 2 | 0.0000 | 1.0000 |
| 27.1 | 1120 | 0.85 | 1.0000 | 1 | 0.9941 | 0.0059 |
| 27.1 | 1800 | 0.13 | 1.0000 | 1 | 0.9940 | 0.0060 |
| 27.5 | 1487 | 2.97 | 1.0000 | 1 | 0.9576 | 0.0424 |
| 27.5 | 2982 | 200 | 1.0000 | 2 | 0.0000 | 1.0000 |
| 27.6 | 5270 | 20.52 | 1.0000 | 2 | 0.0000 | 1.0000 |
| 27.8 | 3328 | 70.15 | 1.0000 | 2 | 0.0000 | 1.0000 |
| 28 | 1593 | 0.31 | 1.0000 | 1 | 0.9938 | 0.0062 |
| 28.2 | 2783 | 0.28 | 1.0000 | 1 | 0.9890 | 0.0110 |
| 28.3 | 176 | 0.81 | 1.0000 | 1 | 0.9883 | 0.0117 |
| 28.3 | 2033 | 0.2 | 1.0000 | 1 | 0.9928 | 0.0072 |
| 28.4 | 2644 | 34.35 | 1.0000 | 2 | 0.0000 | 1.0000 |
| 28.4 | 4633 | 61.35 | 1.0000 | 2 | 0.0000 | 1.0000 |
| 28.5 | 817 | 0.55 | 1.0000 | 1 | 0.9925 | 0.0075 |
| 28.5 | 1252 | 53.31 | 1.0000 | 2 | 0.0000 | 1.0000 |
| 28.6 | 1244 | 1.79 | 1.0000 | 1 | 0.9888 | 0.0112 |
| 28.6 | 1422 | 0.24 | 1.0000 | 1 | 0.9933 | 0.0067 |
| 28.7 | 397 | 0.27 | 1.0000 | 1 | 0.9897 | 0.0103 |
| 28.8 | 7230 | 12.2 | 1.0000 | 2 | 0.0000 | 1.0000 |
| 28.9 | 3242 | 5.15 | 1.0000 | 2 | 0.0309 | 0.9691 |
| 29 | 1587 | 1.72 | 1.0000 | 1 | 0.9891 | 0.0109 |
| 29.1 | 2875 | 1.52 | 1.0000 | 1 | 0.9822 | 0.0178 |
| 29.3 | 1887 | 1.01 | 1.0000 | 1 | 0.9921 | 0.0079 |
| 29.5 | 1591 | 0.79 | 1.0000 | 1 | 0.9927 | 0.0073 |
| 29.5 | 2185 | 49.36 | 1.0000 | 2 | 0.0000 | 1.0000 |
| 29.5 | 2609 | 2.86 | 1.0000 | 1 | 0.9341 | 0.0659 |
| 29.5 | 4502 | 71.2 | 1.0000 | 2 | 0.0000 | 1.0000 |
| 29.7 | 986 | 4.14 | 1.0000 | 1 | 0.5738 | 0.4262 |
| 29.9 | 249 | 0.69 | 1.0000 | 1 | 0.9869 | 0.0131 |
| 29.9 | 964 | 32.05 | 1.0000 | 2 | 0.0000 | 1.0000 |
| 29.9 | 1616 | 0.18 | 1.0000 | 1 | 0.9920 | 0.0080 |
| 30 | 1384 | 31.73 | 1.0000 | 2 | 0.0000 | 1.0000 |
| 30 | 4688 | 0.93 | 1.0000 | 1 | 0.7936 | 0.2064 |
| 30.2 | 906 | 0.94 | 1.0000 | 1 | 0.9907 | 0.0093 |
| 30.2 | 1712 | 1.68 | 1.0000 | 1 | 0.9877 | 0.0123 |
| 30.5 | 762 | 1.82 | 1.0000 | 1 | 0.9829 | 0.0171 |
| 30.7 | 2468 | 5.34 | 1.0000 | 2 | 0.0273 | 0.9727 |
| 30.7 | 5020 | 24.37 | 1.0000 | 2 | 0.0000 | 1.0000 |
| 30.8 | 1121 | 5.31 | 1.0000 | 2 | 0.0383 | 0.9617 |
| 31 | 1569 | 23.95 | 1.0000 | 2 | 0.0000 | 1.0000 |
| 31.1 | 2284 | 2.66 | 1.0000 | 1 | 0.9514 | 0.0486 |
| 31.1 | 2765 | 98 | 1.0000 | 2 | 0.0000 | 1.0000 |
| 31.4 | 4852 | 38.8 | 1.0000 | 2 | 0.0000 | 1.0000 |
| 31.6 | 1687 | 29.18 | 1.0000 | 2 | 0.0000 | 1.0000 |
| 31.9 | 1386 | 18.68 | 1.0000 | 2 | 0.0000 | 1.0000 |
| 32.3 | 13230 | 8.93 | 1.0000 | 2 | 0.0000 | 1.0000 |
| 32.5 | 1180 | 12.95 | 1.0000 | 2 | 0.0000 | 1.0000 |
| 32.5 | 3037 | 0.47 | 1.0000 | 1 | 0.9771 | 0.0229 |
| 32.5 | 4058 | 10.14 | 1.0000 | 2 | 0.0000 | 1.0000 |
| 32.5 | 5880 | 56.79 | 1.0000 | 2 | 0.0000 | 1.0000 |
| 32.8 | 2450 | 1.92 | 1.0000 | 1 | 0.9724 | 0.0276 |
| 33.5 | 5000 | 0.37 | 1.0000 | 1 | 0.5559 | 0.4441 |
| 33.8 | 4902 | 11.59 | 1.0000 | 2 | 0.0000 | 1.0000 |
| 33.9 | 5082 | 0.14 | 1.0000 | 2 | 0.4808 | 0.5192 |
| 34 | 1704 | 0.14 | 1.0000 | 1 | 0.9864 | 0.0136 |
| 34.1 | 3498 | 6.58 | 1.0000 | 2 | 0.0001 | 0.9999 |
| 34.3 | 1927 | 0.08 | 1.0000 | 1 | 0.9849 | 0.0151 |
| 34.6 | 1788 | 12.27 | 1.0000 | 2 | 0.0000 | 1.0000 |
| 34.7 | 4270 | 7.14 | 1.0000 | 2 | 0.0000 | 1.0000 |
| 34.9 | 1206 | 1.45 | 1.0000 | 1 | 0.9807 | 0.0193 |
| 35.7 | 830 | 7.25 | 1.0000 | 2 | 0.0000 | 1.0000 |
| 36 | 729 | 2.63 | 1.0000 | 1 | 0.9143 | 0.0857 |
| 36.3 | 3264 | 0.64 | 1.0000 | 1 | 0.9520 | 0.0480 |
| 36.9 | 1046 | 5.26 | 1.0000 | 2 | 0.0201 | 0.9799 |
| 37 | 1887 | 35.08 | 1.0000 | 2 | 0.0000 | 1.0000 |
| 37.8 | 1507 | 4.5 | 1.0000 | 2 | 0.1639 | 0.8361 |
| 37.9 | 2830 | 0.85 | 1.0000 | 1 | 0.9606 | 0.0394 |
| 38 | 546 | 5.26 | 1.0000 | 2 | 0.0135 | 0.9865 |
| 39 | 1836 | 12.52 | 1.0000 | 2 | 0.0000 | 1.0000 |
| 39 | 2409 | 2.64 | 1.0000 | 1 | 0.8653 | 0.1347 |
| 39.1 | 1807 | 55.61 | 1.0000 | 2 | 0.0000 | 1.0000 |
| 39.3 | 1805 | 0.84 | 1.0000 | 1 | 0.9723 | 0.0277 |
| 39.7 | 1274 | 1.78 | 1.0000 | 1 | 0.9532 | 0.0468 |
| 40.1 | 1092 | 28.06 | 1.0000 | 2 | 0.0000 | 1.0000 |
| 40.5 | 1813 | 25.54 | 1.0000 | 2 | 0.0000 | 1.0000 |
| 40.5 | 7810 | 7.01 | 1.0000 | 2 | 0.0000 | 1.0000 |
| 40.6 | 2821 | 2.06 | 1.0000 | 1 | 0.8849 | 0.1151 |
| 40.8 | 737 | 1.52 | 1.0000 | 1 | 0.9458 | 0.0542 |
| 41.4 | 799 | 1.05 | 1.0000 | 1 | 0.9543 | 0.0457 |
| 41.6 | 5127 | 117.9 | 1.0000 | 2 | 0.0000 | 1.0000 |
| 41.7 | 3725 | 77.96 | 1.0000 | 2 | 0.0000 | 1.0000 |
| 42.5 | 2381 | 1.77 | 1.0000 | 1 | 0.9132 | 0.0868 |
| 43.9 | 1771 | 0.76 | 1.0000 | 1 | 0.9463 | 0.0537 |
| 44.6 | 2328 | 3.68 | 1.0000 | 2 | 0.2968 | 0.7032 |
| 44.7 | 1160 | 0.26 | 1.0000 | 1 | 0.9372 | 0.0628 |
| 45.1 | 1142 | 5.71 | 1.0000 | 2 | 0.0013 | 0.9987 |
| 48.4 | 1266 | 1.47 | 1.0000 | 1 | 0.8632 | 0.1368 |
| 51.8 | 8253 | 24.35 | 1.0000 | 2 | 0.0000 | 1.0000 |

#### Model3 - LL = -11133.0585

|  |  |  |  |
| --- | --- | --- | --- |
| **2-Cluster 1-CFactor Model** | | | |
|  |  |  |  |
| **Number of cases** | 719 |  |  |
| **Number of parameters (Npar)** | 16 |  |  |
| **Activated Constraints** | 0 |  |  |
| **Robustness Effect** | 1.8169 |  |  |
| **Random Seed** | 480308 |  |  |
| **Best Start Seed** | 144217 |  |  |
|  |  |  |  |
| **Log-likelihood Statistics** |  |  |  |
| **Log-likelihood (LL)** | -11133.0585 |  |  |
| **Log-prior** | -126.9644 |  |  |
| **Log-posterior** | -11260.0229 |  |  |
| **BIC (based on LL)** | 22371.3627 |  |  |
| **AIC (based on LL)** | 22298.1169 |  |  |
| **AIC3 (based on LL)** | 22314.1169 |  |  |
| **CAIC (based on LL)** | 22387.3627 |  |  |
|  |  |  |  |
| **Classification Statistics** | **Clusters** | **CFactor1** |  |
| **Classification errors** | 0.0189 |  |  |
| **Reduction of errors (Lambda)** | 0.9287 |  |  |
| **Entropy R-squared** | 0.9097 |  |  |
| **Standard R-squared** | 0.9271 | 0.2556 |  |
| **Classification log-likelihood** | -11170.5952 |  |  |
| **AWE** | 22599.6819 |  |  |
|  |  |  |  |
| **Classification Table** | **Modal** |  |  |
| **Probabilistic** | **Cluster1** | **Cluster2** | **Total** |
| **Cluster1** | 523.1920 | 5.7560 | 528.9480 |
| **Cluster2** | 7.8080 | 182.2440 | 190.0520 |
| **Total** | 531.0000 | 188.0000 | 719.0000 |
|  |  |  |  |
| **Files** |  |  |  |
| **Infile** | C:\Users\Fabian Jaimes\Documents\Johana\Disepsis\DISEPSIS\Clases latentes\Clases latentes\_dic16\disepsis\_definitiva\_CL.sav | | |
| **Output** | C:\Users\Fabian Jaimes\Documents\Johana\Disepsis\DISEPSIS\Clases latentes\Clases latentes\_dic16\data3.sav | | |
|  |  |  |  |
| **Variable Detail** |  |  |  |
| **3 Indicators** |  |  |  |
| **prote�na\_cm1** | Continuous |  |  |
| **d�mero\_dm1** | Continuous |  |  |
| **procalcitonina\_m1** | Continuous |  |  |

#### Parameters

|  |  |  |  |  |  |
| --- | --- | --- | --- | --- | --- |
| **Models for Indicators** |  |  |  |  |  |
|  | **Cluster1** | **Cluster2** | **Wald** | **p-value** | **R�** |
| **prote�na\_cm1** |  |  |  |  |  |
|  | -3.9723 | 3.9723 | 54.2568 | 1.8e-13 | 0.2717 |
| **d�mero\_dm1** |  |  |  |  |  |
|  | -1226.2253 | 1226.2253 | 51.2658 | 8.1e-13 | 0.1606 |
| **procalcitonina\_m1** |  |  |  |  |  |
|  | -14.7164 | 14.7164 | 73.9924 | 7.8e-18 | 0.2672 |
| **CFactor1 : prote�na\_cm1** |  |  |  |  |  |
|  | 4.4828 | 4.4828 | 3.5062 | 0.061 |  |
| **CFactor1 : d�mero\_dm1** |  |  |  |  |  |
|  | 131.7103 | 131.7103 | 3.2082 | 0.073 |  |
| **CFactor1 : procalcitonina\_m1** |  |  |  |  |  |
|  | 0.6730 | 0.6730 | 2.9663 | 0.085 |  |
|  |  |  |  |  |  |
| **Intercepts** | **Overall** | **Wald** | **p-value** |  |  |
| **prote�na\_cm1** |  |  |  |  |  |
|  | 14.3501 | 922.0819 | 1.6e-202 |  |  |
| **d�mero\_dm1** |  |  |  |  |  |
|  | 2922.6966 | 270.0871 | 1.1e-60 |  |  |
| **procalcitonina\_m1** |  |  |  |  |  |
|  | 15.3247 | 77.1701 | 1.6e-18 |  |  |
|  |  |  |  |  |  |
| **Error Variances** | **Cluster1** | **Cluster2** |  |  |  |
| **prote�na\_cm1** | 78.8252 | 108.9223 |  |  |  |
| **d�mero\_dm1** | 1333989.0888 | 19779957.7873 |  |  |  |
| **procalcitonina\_m1** | 1.0558 | 1750.8147 |  |  |  |
|  |  |  |  |  |  |
|  |  |  |  |  |  |
| **Model for Clusters** |  |  |  |  |  |
| **Intercept** | **Cluster1** | **Cluster2** | **Wald** | **p-value** |  |
|  | 0.5110 | -0.5110 | 99.7936 | 1.7e-23 |  |
|  |  |  |  |  |  |

#### Loadings

|  |  |  |  |
| --- | --- | --- | --- |
| **Loadings** | **Clusters** | **CFactor1** | **R�** |
| **prote�na\_cm1** | 0.3211 | 0.4107 | 0.2717 |
| **d�mero\_dm1** | 0.3978 | 0.0484 | 0.1606 |
| **procalcitonina\_m1** | 0.5162 | 0.0268 | 0.2672 |

#### Profile

|  |  |  |
| --- | --- | --- |
|  | **Cluster1** | **Cluster2** |
| **Cluster Size** | 0.7353 | 0.2647 |
| **Indicators** |  |  |
| **prote�na\_cm1** |  |  |
| **Mean** | 10.3778 | 18.3224 |
| **d�mero\_dm1** |  |  |
| **Mean** | 1696.4713 | 4148.9219 |
| **procalcitonina\_m1** |  |  |
| **Mean** | 0.6083 | 30.0411 |

#### ProbMeans

|  |  |  |  |
| --- | --- | --- | --- |
|  | **Cluster1** | **Cluster2** | **CFactor1** |
| **Overall** | 0.7353 | 0.2647 | 0.0000 |
| **Indicators** |  |  |  |
| **prote�na\_cm1** |  |  |  |
| **0.100 - 2.200** | 0.9189 | 0.0811 | -0.4576 |
| **2.300 - 6.500** | 0.8241 | 0.1759 | -0.2840 |
| **6.600 - 13** | 0.7583 | 0.2417 | -0.0972 |
| **13.10 - 22.60** | 0.6831 | 0.3169 | 0.1908 |
| **22.90 - 51.80** | 0.4921 | 0.5079 | 0.6513 |
| **d�mero\_dm1** |  |  |  |
| **25 - 870** | 0.8862 | 0.1138 | -0.1439 |
| **879 - 1308** | 0.8305 | 0.1695 | -0.0175 |
| **1311 - 1966** | 0.8079 | 0.1921 | 0.0056 |
| **1975 - 3182** | 0.7442 | 0.2558 | 0.0823 |
| **3190 - 3e+004** | 0.4105 | 0.5895 | 0.0725 |
| **procalcitonina\_m1** |  |  |  |
| **0.0400 - 0.0500** | 0.9718 | 0.0282 | -0.4224 |
| **0.0600 - 0.230** | 0.9515 | 0.0485 | -0.2266 |
| **0.240 - 0.790** | 0.9601 | 0.0399 | -0.0012 |
| **0.810 - 6.520** | 0.7964 | 0.2036 | 0.6120 |
| **6.580 - 200** | 0.0000 | 1.0000 | 0.0325 |

#### Bivariate Residuals

|  |  |  |  |
| --- | --- | --- | --- |
| **Indicators** | **prote�na\_cm1** | **d�mero\_dm1** | **procalcitonina\_m1** |
| **prote�na\_cm1** | . |  |  |
| **d�mero\_dm1** | 2.5169 | . |  |
| **procalcitonina\_m1** | 0.0938 | 0.3993 | . |

#### Classification

|  |  |  |  |  |  |  |  |
| --- | --- | --- | --- | --- | --- | --- | --- |
| **prote�na\_cm1** | **d�mero\_dm1** | **procalcitonina\_m1** | **ObsFreq** | **Modal** | **Cluster1** | **Cluster2** | **CFactor1** |
| 0.1 | 209 | 0.17 | 1.0000 | 1 | 0.9987 | 0.0013 | -0.5957 |
| 0.1 | 233 | 14.62 | 1.0000 | 2 | 0.0000 | 1.0000 | -0.6595 |
| 0.1 | 295 | 0.05 | 1.0000 | 1 | 0.9988 | 0.0012 | -0.6357 |
| 0.1 | 805 | 0.05 | 1.0000 | 1 | 0.9991 | 0.0009 | -0.6061 |
| 0.1 | 975 | 0.05 | 1.0000 | 1 | 0.9992 | 0.0008 | -0.5962 |
| 0.1 | 1010 | 0.28 | 1.0000 | 1 | 0.9992 | 0.0008 | -0.5078 |
| 0.1 | 1038 | 0.16 | 1.0000 | 1 | 0.9992 | 0.0008 | -0.5512 |
| 0.1 | 1117 | 0.1 | 1.0000 | 1 | 0.9992 | 0.0008 | -0.5692 |
| 0.1 | 1212 | 0.1 | 1.0000 | 1 | 0.9992 | 0.0008 | -0.5636 |
| 0.1 | 1454 | 0.05 | 1.0000 | 1 | 0.9993 | 0.0007 | -0.5683 |
| 0.1 | 1546 | 0.26 | 1.0000 | 1 | 0.9993 | 0.0007 | -0.4841 |
| 0.1 | 1604 | 0.11 | 1.0000 | 1 | 0.9993 | 0.0007 | -0.5371 |
| 0.1 | 1891 | 1.13 | 1.0000 | 1 | 0.9990 | 0.0010 | -0.1376 |
| 0.1 | 2648 | 0.05 | 1.0000 | 1 | 0.9988 | 0.0012 | -0.4990 |
| 0.1 | 4787 | 0.05 | 1.0000 | 1 | 0.9655 | 0.0345 | -0.3835 |
| 0.2 | 187 | 0.05 | 1.0000 | 1 | 0.9987 | 0.0013 | -0.6387 |
| 0.2 | 217 | 0.05 | 1.0000 | 1 | 0.9987 | 0.0013 | -0.6369 |
| 0.2 | 841 | 0.15 | 1.0000 | 1 | 0.9992 | 0.0008 | -0.5631 |
| 0.2 | 911 | 0.05 | 1.0000 | 1 | 0.9992 | 0.0008 | -0.5966 |
| 0.2 | 955 | 0.05 | 1.0000 | 1 | 0.9992 | 0.0008 | -0.5940 |
| 0.2 | 1046 | 0.27 | 1.0000 | 1 | 0.9992 | 0.0008 | -0.5061 |
| 0.2 | 1297 | 0.05 | 1.0000 | 1 | 0.9992 | 0.0008 | -0.5741 |
| 0.2 | 1690 | 0.05 | 1.0000 | 1 | 0.9992 | 0.0008 | -0.5513 |
| 0.2 | 1815 | 0.05 | 1.0000 | 1 | 0.9992 | 0.0008 | -0.5440 |
| 0.2 | 2571 | 0.05 | 1.0000 | 1 | 0.9988 | 0.0012 | -0.5001 |
| 0.2 | 2587 | 17.12 | 1.0000 | 2 | 0.0000 | 1.0000 | -0.6420 |
| 0.2 | 2803 | 0.05 | 1.0000 | 1 | 0.9986 | 0.0014 | -0.4866 |
| 0.2 | 2999 | 0.1 | 1.0000 | 1 | 0.9983 | 0.0017 | -0.4566 |
| 0.2 | 3107 | 1.82 | 1.0000 | 1 | 0.9960 | 0.0040 | 0.1928 |
| 0.2 | 4170 | 0.3 | 1.0000 | 1 | 0.9907 | 0.0093 | -0.3160 |
| 0.3 | 369 | 0.26 | 1.0000 | 1 | 0.9989 | 0.0011 | -0.5459 |
| 0.3 | 577 | 0.52 | 1.0000 | 1 | 0.9990 | 0.0010 | -0.4362 |
| 0.3 | 821 | 0.5 | 1.0000 | 1 | 0.9991 | 0.0009 | -0.4295 |
| 0.3 | 852 | 0.05 | 1.0000 | 1 | 0.9992 | 0.0008 | -0.5966 |
| 0.3 | 884 | 0.05 | 1.0000 | 1 | 0.9992 | 0.0008 | -0.5948 |
| 0.3 | 919 | 0.09 | 1.0000 | 1 | 0.9992 | 0.0008 | -0.5777 |
| 0.3 | 934 | 0.05 | 1.0000 | 1 | 0.9992 | 0.0008 | -0.5919 |
| 0.3 | 1097 | 0.05 | 1.0000 | 1 | 0.9992 | 0.0008 | -0.5824 |
| 0.3 | 2418 | 0.05 | 1.0000 | 1 | 0.9990 | 0.0010 | -0.5056 |
| 0.3 | 2980 | 0.22 | 1.0000 | 1 | 0.9983 | 0.0017 | -0.4093 |
| 0.3 | 3715 | 0.05 | 1.0000 | 1 | 0.9955 | 0.0045 | -0.4309 |
| 0.4 | 162 | 0.18 | 1.0000 | 1 | 0.9987 | 0.0013 | -0.5846 |
| 0.4 | 791 | 0.05 | 1.0000 | 1 | 0.9991 | 0.0009 | -0.5968 |
| 0.4 | 1062 | 0.27 | 1.0000 | 1 | 0.9992 | 0.0008 | -0.4985 |
| 0.4 | 1101 | 0.07 | 1.0000 | 1 | 0.9992 | 0.0008 | -0.5713 |
| 0.5 | 87 | 0.05 | 1.0000 | 1 | 0.9986 | 0.0014 | -0.6344 |
| 0.5 | 161 | 114.8 | 1.0000 | 2 | 0.0000 | 1.0000 | -0.6136 |
| 0.5 | 778 | 0.05 | 1.0000 | 1 | 0.9991 | 0.0009 | -0.5942 |
| 0.5 | 808 | 0.05 | 1.0000 | 1 | 0.9991 | 0.0009 | -0.5925 |
| 0.5 | 1180 | 1.11 | 1.0000 | 1 | 0.9990 | 0.0010 | -0.1731 |
| 0.5 | 1444 | 0.07 | 1.0000 | 1 | 0.9992 | 0.0008 | -0.5480 |
| 0.5 | 1617 | 0.1 | 1.0000 | 1 | 0.9992 | 0.0008 | -0.5267 |
| 0.5 | 1726 | 0.05 | 1.0000 | 1 | 0.9992 | 0.0008 | -0.5391 |
| 0.5 | 1966 | 0.05 | 1.0000 | 1 | 0.9992 | 0.0008 | -0.5252 |
| 0.5 | 20000 | 0.05 | 1.0000 | 2 | 0.0000 | 1.0000 | -0.5394 |
| 0.6 | 884 | 0.18 | 1.0000 | 1 | 0.9992 | 0.0008 | -0.5359 |
| 0.6 | 1147 | 0.06 | 1.0000 | 1 | 0.9992 | 0.0008 | -0.5657 |
| 0.6 | 1160 | 0.05 | 1.0000 | 1 | 0.9992 | 0.0008 | -0.5687 |
| 0.6 | 1187 | 0.05 | 1.0000 | 1 | 0.9992 | 0.0008 | -0.5671 |
| 0.6 | 1954 | 0.83 | 1.0000 | 1 | 0.9991 | 0.0009 | -0.2298 |
| 0.6 | 2799 | 0.2 | 1.0000 | 1 | 0.9986 | 0.0014 | -0.4172 |
| 0.6 | 3431 | 4.92 | 1.0000 | 2 | 0.1929 | 0.8071 | -0.2377 |
| 0.6 | 3675 | 0.21 | 1.0000 | 1 | 0.9959 | 0.0041 | -0.3633 |
| 0.6 | 10000 | 0.1 | 1.0000 | 2 | 0.0000 | 1.0000 | -0.5920 |
| 0.7 | 224 | 0.05 | 1.0000 | 1 | 0.9987 | 0.0013 | -0.6197 |
| 0.7 | 704 | 0.05 | 1.0000 | 1 | 0.9991 | 0.0009 | -0.5918 |
| 0.7 | 1360 | 0.05 | 1.0000 | 1 | 0.9992 | 0.0008 | -0.5537 |
| 0.7 | 1842 | 18.52 | 1.0000 | 2 | 0.0000 | 1.0000 | -0.6284 |
| 0.8 | 232 | 0.17 | 1.0000 | 1 | 0.9987 | 0.0013 | -0.5709 |
| 0.8 | 435 | 0.05 | 1.0000 | 1 | 0.9989 | 0.0011 | -0.6041 |
| 0.8 | 930 | 0.05 | 1.0000 | 1 | 0.9992 | 0.0008 | -0.5753 |
| 0.8 | 3070 | 2.92 | 1.0000 | 1 | 0.9819 | 0.0181 | 0.6047 |
| 0.9 | 302 | 0.05 | 1.0000 | 1 | 0.9988 | 0.0012 | -0.6085 |
| 0.9 | 1246 | 0.35 | 1.0000 | 1 | 0.9992 | 0.0008 | -0.4410 |
| 0.9 | 1406 | 0.05 | 1.0000 | 1 | 0.9992 | 0.0008 | -0.5443 |
| 0.9 | 1630 | 1.05 | 1.0000 | 1 | 0.9991 | 0.0009 | -0.1560 |
| 0.9 | 1771 | 0.21 | 1.0000 | 1 | 0.9992 | 0.0008 | -0.4630 |
| 0.9 | 2232 | 0.15 | 1.0000 | 1 | 0.9991 | 0.0009 | -0.4588 |
| 0.9 | 2882 | 0.05 | 1.0000 | 1 | 0.9984 | 0.0016 | -0.4586 |
| 0.9 | 3574 | 0.17 | 1.0000 | 1 | 0.9964 | 0.0036 | -0.3739 |
| 0.9 | 4947 | 124.3 | 1.0000 | 2 | 0.0000 | 1.0000 | -0.5697 |
| 1 | 249 | 0.05 | 1.0000 | 1 | 0.9987 | 0.0013 | -0.6082 |
| 1 | 737 | 0.37 | 1.0000 | 1 | 0.9991 | 0.0009 | -0.4598 |
| 1 | 1008 | 0.05 | 1.0000 | 1 | 0.9992 | 0.0008 | -0.5641 |
| 1 | 1559 | 0.05 | 1.0000 | 1 | 0.9992 | 0.0008 | -0.5321 |
| 1 | 1963 | 0.76 | 1.0000 | 1 | 0.9991 | 0.0009 | -0.2421 |
| 1 | 2069 | 0.27 | 1.0000 | 1 | 0.9991 | 0.0009 | -0.4198 |
| 1 | 3104 | 0.05 | 1.0000 | 1 | 0.9980 | 0.0020 | -0.4424 |
| 1.1 | 285 | 0.05 | 1.0000 | 1 | 0.9988 | 0.0012 | -0.6028 |
| 1.1 | 489 | 0.16 | 1.0000 | 1 | 0.9989 | 0.0011 | -0.5497 |
| 1.1 | 1313 | 0.05 | 1.0000 | 1 | 0.9992 | 0.0008 | -0.5430 |
| 1.1 | 1447 | 1.83 | 1.0000 | 1 | 0.9982 | 0.0018 | 0.1322 |
| 1.2 | 199 | 0.26 | 1.0000 | 1 | 0.9987 | 0.0013 | -0.5257 |
| 1.2 | 895 | 0.05 | 1.0000 | 1 | 0.9991 | 0.0009 | -0.5640 |
| 1.2 | 964 | 0.05 | 1.0000 | 1 | 0.9992 | 0.0008 | -0.5600 |
| 1.2 | 1546 | 0.05 | 1.0000 | 1 | 0.9992 | 0.0008 | -0.5261 |
| 1.2 | 1779 | 43.39 | 1.0000 | 2 | 0.0000 | 1.0000 | -0.6033 |
| 1.2 | 2122 | 0.13 | 1.0000 | 1 | 0.9991 | 0.0009 | -0.4626 |
| 1.2 | 2341 | 0.12 | 1.0000 | 1 | 0.9990 | 0.0010 | -0.4537 |
| 1.2 | 4093 | 0.05 | 1.0000 | 1 | 0.9913 | 0.0087 | -0.3798 |
| 1.2 | 5011 | 0.05 | 1.0000 | 1 | 0.9403 | 0.0597 | -0.3408 |
| 1.2 | 5018 | 3.51 | 1.0000 | 2 | 0.3927 | 0.6073 | 0.0199 |
| 1.3 | 1149 | 0.05 | 1.0000 | 1 | 0.9992 | 0.0008 | -0.5458 |
| 1.4 | 2659 | 0.05 | 1.0000 | 1 | 0.9987 | 0.0013 | -0.4548 |
| 1.4 | 3086 | 0.05 | 1.0000 | 1 | 0.9980 | 0.0020 | -0.4301 |
| 1.5 | 1084 | 0.05 | 1.0000 | 1 | 0.9992 | 0.0008 | -0.5429 |
| 1.5 | 1131 | 0.37 | 1.0000 | 1 | 0.9992 | 0.0008 | -0.4201 |
| 1.5 | 1323 | 0.05 | 1.0000 | 1 | 0.9992 | 0.0008 | -0.5290 |
| 1.5 | 2330 | 0.05 | 1.0000 | 1 | 0.9990 | 0.0010 | -0.4705 |
| 1.5 | 3452 | 4.18 | 1.0000 | 1 | 0.6833 | 0.3167 | 0.5945 |
| 1.5 | 3517 | 0.05 | 1.0000 | 1 | 0.9965 | 0.0035 | -0.4020 |
| 1.5 | 5036 | 1.77 | 1.0000 | 1 | 0.8981 | 0.1019 | 0.2393 |
| 1.6 | 1188 | 0.05 | 1.0000 | 1 | 0.9992 | 0.0008 | -0.5335 |
| 1.6 | 2004 | 0.05 | 1.0000 | 1 | 0.9991 | 0.0009 | -0.4861 |
| 1.6 | 3075 | 1.62 | 1.0000 | 1 | 0.9968 | 0.0032 | 0.1636 |
| 1.7 | 613 | 0.53 | 1.0000 | 1 | 0.9990 | 0.0010 | -0.3835 |
| 1.7 | 2419 | 0.21 | 1.0000 | 1 | 0.9989 | 0.0011 | -0.3986 |
| 1.7 | 4450 | 0.05 | 1.0000 | 1 | 0.9823 | 0.0177 | -0.3447 |
| 1.7 | 4893 | 0.05 | 1.0000 | 1 | 0.9537 | 0.0463 | -0.3269 |
| 1.8 | 1160 | 0.93 | 1.0000 | 1 | 0.9991 | 0.0009 | -0.1982 |
| 1.8 | 1172 | 0.05 | 1.0000 | 1 | 0.9992 | 0.0008 | -0.5278 |
| 1.8 | 1865 | 0.24 | 1.0000 | 1 | 0.9992 | 0.0008 | -0.4162 |
| 1.8 | 2171 | 0.08 | 1.0000 | 1 | 0.9990 | 0.0010 | -0.4584 |
| 1.8 | 2479 | 0.05 | 1.0000 | 1 | 0.9988 | 0.0012 | -0.4518 |
| 1.8 | 2506 | 0.05 | 1.0000 | 1 | 0.9988 | 0.0012 | -0.4502 |
| 1.9 | 1506 | 0.14 | 1.0000 | 1 | 0.9992 | 0.0008 | -0.4712 |
| 1.9 | 1646 | 0.05 | 1.0000 | 1 | 0.9992 | 0.0008 | -0.4968 |
| 1.9 | 1766 | 0.05 | 1.0000 | 1 | 0.9992 | 0.0008 | -0.4899 |
| 1.9 | 3178 | 0.05 | 1.0000 | 1 | 0.9977 | 0.0023 | -0.4080 |
| 2 | 891 | 0.06 | 1.0000 | 1 | 0.9991 | 0.0009 | -0.5336 |
| 2 | 1885 | 0.52 | 1.0000 | 1 | 0.9992 | 0.0008 | -0.3032 |
| 2.1 | 175 | 0.05 | 1.0000 | 1 | 0.9986 | 0.0014 | -0.5757 |
| 2.1 | 412 | 0.1 | 1.0000 | 1 | 0.9988 | 0.0012 | -0.5431 |
| 2.1 | 1712 | 0.05 | 1.0000 | 1 | 0.9992 | 0.0008 | -0.4863 |
| 2.1 | 1897 | 0.05 | 1.0000 | 1 | 0.9991 | 0.0009 | -0.4756 |
| 2.1 | 6030 | 0.05 | 1.0000 | 2 | 0.4631 | 0.5369 | -0.4107 |
| 2.2 | 254 | 0.18 | 1.0000 | 1 | 0.9987 | 0.0013 | -0.5190 |
| 2.2 | 349 | 0.05 | 1.0000 | 1 | 0.9988 | 0.0012 | -0.5622 |
| 2.2 | 707 | 0.32 | 1.0000 | 1 | 0.9990 | 0.0010 | -0.4401 |
| 2.2 | 845 | 0.17 | 1.0000 | 1 | 0.9991 | 0.0009 | -0.4883 |
| 2.2 | 895 | 1.02 | 1.0000 | 1 | 0.9989 | 0.0011 | -0.1665 |
| 2.2 | 986 | 0.05 | 1.0000 | 1 | 0.9991 | 0.0009 | -0.5252 |
| 2.2 | 2492 | 5.34 | 1.0000 | 2 | 0.1269 | 0.8731 | -0.3070 |
| 2.3 | 1188 | 0.05 | 1.0000 | 1 | 0.9992 | 0.0008 | -0.5101 |
| 2.3 | 1953 | 0.05 | 1.0000 | 1 | 0.9991 | 0.0009 | -0.4656 |
| 2.3 | 3345 | 0.08 | 1.0000 | 1 | 0.9972 | 0.0028 | -0.3738 |
| 2.3 | 3774 | 3.37 | 1.0000 | 1 | 0.9071 | 0.0929 | 0.7525 |
| 2.4 | 1250 | 0.08 | 1.0000 | 1 | 0.9992 | 0.0008 | -0.4919 |
| 2.4 | 1434 | 0.63 | 1.0000 | 1 | 0.9992 | 0.0008 | -0.2747 |
| 2.6 | 817 | 0.1 | 1.0000 | 1 | 0.9990 | 0.0010 | -0.5028 |
| 2.6 | 937 | 6.78 | 1.0000 | 2 | 0.0004 | 0.9996 | -0.5703 |
| 2.6 | 1182 | 0.28 | 1.0000 | 1 | 0.9992 | 0.0008 | -0.4140 |
| 2.6 | 1515 | 1.81 | 1.0000 | 1 | 0.9983 | 0.0017 | 0.1789 |
| 2.6 | 3269 | 0.34 | 1.0000 | 1 | 0.9975 | 0.0025 | -0.2707 |
| 2.7 | 924 | 40.46 | 1.0000 | 2 | 0.0000 | 1.0000 | -0.5570 |
| 2.7 | 1294 | 0.06 | 1.0000 | 1 | 0.9992 | 0.0008 | -0.4867 |
| 2.8 | 131 | 0.27 | 1.0000 | 1 | 0.9985 | 0.0015 | -0.4723 |
| 2.8 | 1007 | 4.43 | 1.0000 | 1 | 0.7426 | 0.2574 | 0.7023 |
| 2.8 | 2023 | 0.16 | 1.0000 | 1 | 0.9991 | 0.0009 | -0.4035 |
| 2.8 | 2620 | 0.05 | 1.0000 | 1 | 0.9987 | 0.0013 | -0.4101 |
| 2.9 | 269 | 0.28 | 1.0000 | 1 | 0.9986 | 0.0014 | -0.4571 |
| 2.9 | 467 | 0.17 | 1.0000 | 1 | 0.9988 | 0.0012 | -0.4869 |
| 2.9 | 704 | 0.05 | 1.0000 | 1 | 0.9990 | 0.0010 | -0.5181 |
| 2.9 | 791 | 0.26 | 1.0000 | 1 | 0.9990 | 0.0010 | -0.4342 |
| 2.9 | 1605 | 0.19 | 1.0000 | 1 | 0.9992 | 0.0008 | -0.4132 |
| 2.9 | 2477 | 0.24 | 1.0000 | 1 | 0.9988 | 0.0012 | -0.3438 |
| 2.9 | 4247 | 11.51 | 1.0000 | 2 | 0.0000 | 1.0000 | -0.5408 |
| 3 | 244 | 0.7 | 1.0000 | 1 | 0.9985 | 0.0015 | -0.2977 |
| 3 | 1011 | 0.33 | 1.0000 | 1 | 0.9991 | 0.0009 | -0.3918 |
| 3 | 1343 | 0.37 | 1.0000 | 1 | 0.9992 | 0.0008 | -0.3575 |
| 3 | 1701 | 0.1 | 1.0000 | 1 | 0.9991 | 0.0009 | -0.4380 |
| 3.1 | 402 | 0.05 | 1.0000 | 1 | 0.9987 | 0.0013 | -0.5290 |
| 3.1 | 2794 | 0.05 | 1.0000 | 1 | 0.9984 | 0.0016 | -0.3900 |
| 3.1 | 3956 | 0.05 | 1.0000 | 1 | 0.9926 | 0.0074 | -0.3238 |
| 3.2 | 1023 | 0.27 | 1.0000 | 1 | 0.9991 | 0.0009 | -0.4069 |
| 3.2 | 2511 | 0.26 | 1.0000 | 1 | 0.9988 | 0.0012 | -0.3243 |
| 3.3 | 882 | 2.65 | 1.0000 | 1 | 0.9937 | 0.0063 | 0.4759 |
| 3.3 | 2350 | 0.4 | 1.0000 | 1 | 0.9989 | 0.0011 | -0.2777 |
| 3.3 | 3175 | 11.49 | 1.0000 | 2 | 0.0000 | 1.0000 | -0.5329 |
| 3.4 | 1630 | 0.05 | 1.0000 | 1 | 0.9991 | 0.0009 | -0.4475 |
| 3.4 | 2037 | 0.05 | 1.0000 | 1 | 0.9990 | 0.0010 | -0.4239 |
| 3.4 | 8550 | 0.42 | 1.0000 | 2 | 0.0000 | 1.0000 | -0.5029 |
| 3.5 | 2849 | 0.06 | 1.0000 | 1 | 0.9983 | 0.0017 | -0.3697 |
| 3.5 | 3750 | 200 | 1.0000 | 2 | 0.0000 | 1.0000 | -0.4617 |
| 3.5 | 4986 | 2.82 | 1.0000 | 1 | 0.7305 | 0.2695 | 0.4386 |
| 3.6 | 316 | 0.18 | 1.0000 | 1 | 0.9986 | 0.0014 | -0.4684 |
| 3.6 | 900 | 4.32 | 1.0000 | 1 | 0.7943 | 0.2057 | 0.7796 |
| 3.6 | 1388 | 0.05 | 1.0000 | 1 | 0.9991 | 0.0009 | -0.4549 |
| 3.6 | 1474 | 0.05 | 1.0000 | 1 | 0.9991 | 0.0009 | -0.4499 |
| 3.6 | 2521 | 0.17 | 1.0000 | 1 | 0.9987 | 0.0013 | -0.3440 |
| 3.7 | 162 | 34.37 | 1.0000 | 2 | 0.0000 | 1.0000 | -0.5286 |
| 3.7 | 758 | 0.1 | 1.0000 | 1 | 0.9990 | 0.0010 | -0.4694 |
| 3.7 | 1730 | 0.05 | 1.0000 | 1 | 0.9991 | 0.0009 | -0.4316 |
| 3.7 | 2138 | 9.48 | 1.0000 | 2 | 0.0000 | 1.0000 | -0.5255 |
| 3.8 | 349 | 0.22 | 1.0000 | 1 | 0.9987 | 0.0013 | -0.4448 |
| 3.8 | 1340 | 0.85 | 1.0000 | 1 | 0.9991 | 0.0009 | -0.1507 |
| 3.8 | 2222 | 0.08 | 1.0000 | 1 | 0.9989 | 0.0011 | -0.3885 |
| 3.8 | 3344 | 0.21 | 1.0000 | 1 | 0.9971 | 0.0029 | -0.2749 |
| 3.9 | 125 | 0.2 | 1.0000 | 1 | 0.9984 | 0.0016 | -0.4620 |
| 3.9 | 199 | 0.15 | 1.0000 | 1 | 0.9985 | 0.0015 | -0.4765 |
| 3.9 | 1254 | 0.12 | 1.0000 | 1 | 0.9991 | 0.0009 | -0.4263 |
| 3.9 | 2318 | 0.05 | 1.0000 | 1 | 0.9988 | 0.0012 | -0.3908 |
| 4.1 | 789 | 15.58 | 1.0000 | 2 | 0.0000 | 1.0000 | -0.5173 |
| 4.1 | 816 | 0.05 | 1.0000 | 1 | 0.9990 | 0.0010 | -0.4714 |
| 4.1 | 1410 | 0.05 | 1.0000 | 1 | 0.9991 | 0.0009 | -0.4368 |
| 4.1 | 5150 | 17.69 | 1.0000 | 2 | 0.0000 | 1.0000 | -0.4921 |
| 4.2 | 590 | 0.41 | 1.0000 | 1 | 0.9989 | 0.0011 | -0.3461 |
| 4.2 | 2041 | 0.05 | 1.0000 | 1 | 0.9990 | 0.0010 | -0.3968 |
| 4.2 | 4937 | 0.16 | 1.0000 | 1 | 0.9457 | 0.0543 | -0.2037 |
| 4.3 | 956 | 0.05 | 1.0000 | 1 | 0.9990 | 0.0010 | -0.4565 |
| 4.4 | 483 | 0.28 | 1.0000 | 1 | 0.9988 | 0.0012 | -0.3944 |
| 4.4 | 950 | 8.2 | 1.0000 | 2 | 0.0000 | 1.0000 | -0.5083 |
| 4.4 | 2120 | 1.36 | 1.0000 | 1 | 0.9986 | 0.0014 | 0.1059 |
| 4.5 | 394 | 1.05 | 1.0000 | 1 | 0.9984 | 0.0016 | -0.1075 |
| 4.5 | 3500 | 27.92 | 1.0000 | 2 | 0.0000 | 1.0000 | -0.4841 |
| 4.5 | 3772 | 5.84 | 1.0000 | 2 | 0.0081 | 0.9919 | -0.4704 |
| 4.5 | 22290 | 0.36 | 1.0000 | 2 | 0.0000 | 1.0000 | -0.3875 |
| 4.6 | 366 | 0.29 | 1.0000 | 1 | 0.9986 | 0.0014 | -0.3908 |
| 4.6 | 5180 | 0.14 | 1.0000 | 1 | 0.9028 | 0.0972 | -0.1973 |
| 4.7 | 261 | 0.74 | 1.0000 | 1 | 0.9984 | 0.0016 | -0.2248 |
| 4.7 | 674 | 0.05 | 1.0000 | 1 | 0.9988 | 0.0012 | -0.4595 |
| 4.7 | 1258 | 0.59 | 1.0000 | 1 | 0.9991 | 0.0009 | -0.2229 |
| 4.7 | 1262 | 0.13 | 1.0000 | 1 | 0.9991 | 0.0009 | -0.3953 |
| 4.7 | 1747 | 0.6 | 1.0000 | 1 | 0.9991 | 0.0009 | -0.1907 |
| 4.8 | 242 | 0.05 | 1.0000 | 1 | 0.9984 | 0.0016 | -0.4813 |
| 4.8 | 1497 | 0.05 | 1.0000 | 1 | 0.9991 | 0.0009 | -0.4083 |
| 4.8 | 5790 | 15.07 | 1.0000 | 2 | 0.0000 | 1.0000 | -0.4650 |
| 4.9 | 1407 | 0.12 | 1.0000 | 1 | 0.9991 | 0.0009 | -0.3839 |
| 4.9 | 1646 | 0.13 | 1.0000 | 1 | 0.9991 | 0.0009 | -0.3663 |
| 4.9 | 1842 | 0.33 | 1.0000 | 1 | 0.9991 | 0.0009 | -0.2798 |
| 5 | 485 | 0.27 | 1.0000 | 1 | 0.9987 | 0.0013 | -0.3779 |
| 5 | 1255 | 0.08 | 1.0000 | 1 | 0.9990 | 0.0010 | -0.4044 |
| 5 | 2054 | 0.05 | 1.0000 | 1 | 0.9989 | 0.0011 | -0.3693 |
| 5 | 2854 | 3.92 | 1.0000 | 1 | 0.8986 | 0.1014 | 0.9680 |
| 5.1 | 179 | 0.05 | 1.0000 | 1 | 0.9983 | 0.0017 | -0.4749 |
| 5.1 | 1045 | 0.86 | 1.0000 | 1 | 0.9989 | 0.0011 | -0.1206 |
| 5.1 | 1455 | 3.9 | 1.0000 | 1 | 0.9370 | 0.0630 | 0.9494 |
| 5.1 | 1951 | 0.05 | 1.0000 | 1 | 0.9990 | 0.0010 | -0.3719 |
| 5.1 | 2142 | 0.96 | 1.0000 | 1 | 0.9988 | 0.0012 | -0.0194 |
| 5.1 | 4699 | 0.23 | 1.0000 | 1 | 0.9674 | 0.0326 | -0.1548 |
| 5.1 | 7150 | 5.91 | 1.0000 | 2 | 0.0000 | 1.0000 | -0.4499 |
| 5.2 | 907 | 0.51 | 1.0000 | 1 | 0.9990 | 0.0010 | -0.2566 |
| 5.2 | 1210 | 0.05 | 1.0000 | 1 | 0.9990 | 0.0010 | -0.4116 |
| 5.2 | 2367 | 0.05 | 1.0000 | 1 | 0.9987 | 0.0013 | -0.3444 |
| 5.3 | 255 | 1.69 | 1.0000 | 1 | 0.9971 | 0.0029 | 0.1505 |
| 5.3 | 1639 | 1.12 | 1.0000 | 1 | 0.9989 | 0.0011 | 0.0182 |
| 5.3 | 1712 | 0.49 | 1.0000 | 1 | 0.9991 | 0.0009 | -0.2139 |
| 5.3 | 3060 | 0.18 | 1.0000 | 1 | 0.9978 | 0.0022 | -0.2522 |
| 5.3 | 3847 | 0.13 | 1.0000 | 1 | 0.9933 | 0.0067 | -0.2263 |
| 5.3 | 10000 | 0.05 | 1.0000 | 2 | 0.0000 | 1.0000 | -0.4289 |
| 5.4 | 1358 | 0.1 | 1.0000 | 1 | 0.9990 | 0.0010 | -0.3775 |
| 5.4 | 1857 | 0.08 | 1.0000 | 1 | 0.9990 | 0.0010 | -0.3560 |
| 5.4 | 2018 | 22.09 | 1.0000 | 2 | 0.0000 | 1.0000 | -0.4631 |
| 5.5 | 25 | 0.05 | 1.0000 | 1 | 0.9980 | 0.0020 | -0.4704 |
| 5.5 | 817 | 0.14 | 1.0000 | 1 | 0.9989 | 0.0011 | -0.3906 |
| 5.5 | 959 | 0.2 | 1.0000 | 1 | 0.9990 | 0.0010 | -0.3599 |
| 5.5 | 1090 | 0.05 | 1.0000 | 1 | 0.9990 | 0.0010 | -0.4085 |
| 5.6 | 882 | 0.05 | 1.0000 | 1 | 0.9989 | 0.0011 | -0.4173 |
| 5.6 | 1208 | 0.1 | 1.0000 | 1 | 0.9990 | 0.0010 | -0.3796 |
| 5.7 | 573 | 0.12 | 1.0000 | 1 | 0.9987 | 0.0013 | -0.4056 |
| 5.7 | 954 | 0.05 | 1.0000 | 1 | 0.9989 | 0.0011 | -0.4097 |
| 5.7 | 2465 | 4.4 | 1.0000 | 1 | 0.7719 | 0.2281 | 0.9090 |
| 5.8 | 927 | 0.51 | 1.0000 | 1 | 0.9989 | 0.0011 | -0.2353 |
| 5.9 | 1348 | 0.05 | 1.0000 | 1 | 0.9990 | 0.0010 | -0.3801 |
| 5.9 | 1420 | 0.06 | 1.0000 | 1 | 0.9990 | 0.0010 | -0.3722 |
| 5.9 | 2136 | 0.41 | 1.0000 | 1 | 0.9989 | 0.0011 | -0.1992 |
| 5.9 | 2145 | 5.1 | 1.0000 | 2 | 0.3192 | 0.6808 | 0.1923 |
| 5.9 | 10000 | 5.07 | 1.0000 | 2 | 0.0000 | 1.0000 | -0.4064 |
| 6 | 3154 | 4.18 | 1.0000 | 1 | 0.7836 | 0.2164 | 0.9073 |
| 6 | 3182 | 0.05 | 1.0000 | 1 | 0.9972 | 0.0028 | -0.2705 |
| 6.1 | 338 | 0.17 | 1.0000 | 1 | 0.9985 | 0.0015 | -0.3871 |
| 6.1 | 14800 | 2.58 | 1.0000 | 2 | 0.0000 | 1.0000 | -0.3734 |
| 6.2 | 172 | 0.11 | 1.0000 | 1 | 0.9982 | 0.0018 | -0.4159 |
| 6.2 | 2268 | 0.1 | 1.0000 | 1 | 0.9988 | 0.0012 | -0.2979 |
| 6.2 | 3224 | 15 | 1.0000 | 2 | 0.0000 | 1.0000 | -0.4309 |
| 6.2 | 3774 | 0.43 | 1.0000 | 1 | 0.9942 | 0.0058 | -0.0881 |
| 6.2 | 15160 | 2.27 | 1.0000 | 2 | 0.0000 | 1.0000 | -0.3680 |
| 6.3 | 171 | 0.74 | 1.0000 | 1 | 0.9981 | 0.0019 | -0.1764 |
| 6.3 | 947 | 0.17 | 1.0000 | 1 | 0.9989 | 0.0011 | -0.3450 |
| 6.3 | 2276 | 0.5 | 1.0000 | 1 | 0.9988 | 0.0012 | -0.1440 |
| 6.3 | 4638 | 0.05 | 1.0000 | 1 | 0.9680 | 0.0320 | -0.1832 |
| 6.4 | 386 | 0.07 | 1.0000 | 1 | 0.9985 | 0.0015 | -0.4118 |
| 6.4 | 1038 | 1.1 | 1.0000 | 1 | 0.9988 | 0.0012 | 0.0125 |
| 6.5 | 121 | 0.35 | 1.0000 | 1 | 0.9981 | 0.0019 | -0.3189 |
| 6.5 | 1464 | 0.83 | 1.0000 | 1 | 0.9990 | 0.0010 | -0.0606 |
| 6.5 | 1769 | 0.44 | 1.0000 | 1 | 0.9990 | 0.0010 | -0.1892 |
| 6.5 | 2719 | 0.05 | 1.0000 | 1 | 0.9982 | 0.0018 | -0.2804 |
| 6.5 | 10000 | 29.02 | 1.0000 | 2 | 0.0000 | 1.0000 | -0.3779 |
| 6.6 | 390 | 2.22 | 1.0000 | 1 | 0.9952 | 0.0048 | 0.3988 |
| 6.6 | 1607 | 0.05 | 1.0000 | 1 | 0.9990 | 0.0010 | -0.3416 |
| 6.6 | 1961 | 0.06 | 1.0000 | 1 | 0.9989 | 0.0011 | -0.3173 |
| 6.6 | 5970 | 0.38 | 1.0000 | 2 | 0.4920 | 0.5080 | -0.1885 |
| 6.7 | 1167 | 0.05 | 1.0000 | 1 | 0.9989 | 0.0011 | -0.3638 |
| 6.7 | 1698 | 0.14 | 1.0000 | 1 | 0.9990 | 0.0010 | -0.2992 |
| 6.7 | 2988 | 0.14 | 1.0000 | 1 | 0.9977 | 0.0023 | -0.2245 |
| 6.7 | 3074 | 0.25 | 1.0000 | 1 | 0.9976 | 0.0024 | -0.1783 |
| 6.8 | 1265 | 0.39 | 1.0000 | 1 | 0.9990 | 0.0010 | -0.2272 |
| 6.8 | 4053 | 0.36 | 1.0000 | 1 | 0.9904 | 0.0096 | -0.0793 |
| 6.9 | 547 | 2.56 | 1.0000 | 1 | 0.9931 | 0.0069 | 0.5431 |
| 6.9 | 1190 | 0.19 | 1.0000 | 1 | 0.9989 | 0.0011 | -0.3033 |
| 6.9 | 1829 | 0.05 | 1.0000 | 1 | 0.9989 | 0.0011 | -0.3187 |
| 6.9 | 3737 | 2.11 | 1.0000 | 1 | 0.9873 | 0.0127 | 0.5539 |
| 7 | 378 | 0.05 | 1.0000 | 1 | 0.9984 | 0.0016 | -0.3997 |
| 7 | 810 | 0.05 | 1.0000 | 1 | 0.9988 | 0.0012 | -0.3745 |
| 7 | 1524 | 0.04 | 1.0000 | 1 | 0.9989 | 0.0011 | -0.3368 |
| 7.1 | 1048 | 0.32 | 1.0000 | 1 | 0.9989 | 0.0011 | -0.2560 |
| 7.1 | 1433 | 0.42 | 1.0000 | 1 | 0.9990 | 0.0010 | -0.1961 |
| 7.2 | 739 | 0.09 | 1.0000 | 1 | 0.9987 | 0.0013 | -0.3570 |
| 7.2 | 783 | 0.05 | 1.0000 | 1 | 0.9987 | 0.0013 | -0.3694 |
| 7.2 | 3855 | 2.9 | 1.0000 | 1 | 0.9571 | 0.0429 | 0.8253 |
| 7.3 | 702 | 0.05 | 1.0000 | 1 | 0.9987 | 0.0013 | -0.3708 |
| 7.3 | 2069 | 0.61 | 1.0000 | 1 | 0.9989 | 0.0011 | -0.0812 |
| 7.4 | 1412 | 0.05 | 1.0000 | 1 | 0.9989 | 0.0011 | -0.3261 |
| 7.4 | 7774 | 0.05 | 1.0000 | 2 | 0.0010 | 0.9990 | -0.3681 |
| 7.4 | 10000 | 0.12 | 1.0000 | 2 | 0.0000 | 1.0000 | -0.3560 |
| 7.5 | 457 | 0.05 | 1.0000 | 1 | 0.9984 | 0.0016 | -0.3783 |
| 7.5 | 1773 | 0.21 | 1.0000 | 1 | 0.9989 | 0.0011 | -0.2418 |
| 7.5 | 5018 | 0.05 | 1.0000 | 1 | 0.9215 | 0.0785 | -0.1339 |
| 7.5 | 5880 | 0.87 | 1.0000 | 1 | 0.5593 | 0.4407 | -0.0283 |
| 7.6 | 101 | 0.05 | 1.0000 | 1 | 0.9979 | 0.0021 | -0.3957 |
| 7.6 | 291 | 0.28 | 1.0000 | 1 | 0.9983 | 0.0017 | -0.2984 |
| 7.6 | 2092 | 6.62 | 1.0000 | 2 | 0.0017 | 0.9983 | -0.3871 |
| 7.7 | 1235 | 31.96 | 1.0000 | 2 | 0.0000 | 1.0000 | -0.3845 |
| 7.7 | 2173 | 0.23 | 1.0000 | 1 | 0.9987 | 0.0013 | -0.2043 |
| 7.7 | 2402 | 0.35 | 1.0000 | 1 | 0.9986 | 0.0014 | -0.1460 |
| 7.7 | 2985 | 0.05 | 1.0000 | 1 | 0.9975 | 0.0025 | -0.2249 |
| 7.7 | 10000 | 0.08 | 1.0000 | 2 | 0.0000 | 1.0000 | -0.3456 |
| 7.8 | 1313 | 29.95 | 1.0000 | 2 | 0.0000 | 1.0000 | -0.3812 |
| 7.8 | 1323 | 56.71 | 1.0000 | 2 | 0.0000 | 1.0000 | -0.3725 |
| 7.8 | 1562 | 0.06 | 1.0000 | 1 | 0.9989 | 0.0011 | -0.3003 |
| 7.8 | 2679 | 5.74 | 1.0000 | 2 | 0.0426 | 0.9574 | -0.2843 |
| 7.9 | 440 | 0.2 | 1.0000 | 1 | 0.9984 | 0.0016 | -0.3096 |
| 7.9 | 3510 | 0.48 | 1.0000 | 1 | 0.9957 | 0.0043 | -0.0272 |
| 7.9 | 4038 | 0.14 | 1.0000 | 1 | 0.9895 | 0.0105 | -0.1253 |
| 8 | 1311 | 0.29 | 1.0000 | 1 | 0.9989 | 0.0011 | -0.2218 |
| 8.1 | 1070 | 0.53 | 1.0000 | 1 | 0.9989 | 0.0011 | -0.1425 |
| 8.1 | 2124 | 2.24 | 1.0000 | 1 | 0.9966 | 0.0034 | 0.5583 |
| 8.2 | 215 | 0.14 | 1.0000 | 1 | 0.9980 | 0.0020 | -0.3352 |
| 8.2 | 1671 | 0.12 | 1.0000 | 1 | 0.9989 | 0.0011 | -0.2580 |
| 8.2 | 3908 | 2.83 | 1.0000 | 1 | 0.9584 | 0.0416 | 0.8382 |
| 8.3 | 981 | 43.49 | 1.0000 | 2 | 0.0000 | 1.0000 | -0.3613 |
| 8.3 | 1663 | 57.58 | 1.0000 | 2 | 0.0000 | 1.0000 | -0.3529 |
| 8.3 | 1768 | 1.97 | 1.0000 | 1 | 0.9977 | 0.0023 | 0.4442 |
| 8.3 | 4616 | 142.4 | 1.0000 | 2 | 0.0000 | 1.0000 | -0.3088 |
| 8.4 | 655 | 0.43 | 1.0000 | 1 | 0.9986 | 0.0014 | -0.1941 |
| 8.4 | 958 | 140.2 | 1.0000 | 2 | 0.0000 | 1.0000 | -0.3266 |
| 8.4 | 2389 | 113.6 | 1.0000 | 2 | 0.0000 | 1.0000 | -0.3272 |
| 8.4 | 2538 | 0.85 | 1.0000 | 1 | 0.9984 | 0.0016 | 0.0728 |
| 8.4 | 2810 | 3.4 | 1.0000 | 1 | 0.9687 | 0.0313 | 1.0032 |
| 8.5 | 3976 | 10.91 | 1.0000 | 2 | 0.0000 | 1.0000 | -0.3481 |
| 8.6 | 239 | 0.65 | 1.0000 | 1 | 0.9981 | 0.0019 | -0.1292 |
| 8.6 | 1500 | 0.05 | 1.0000 | 1 | 0.9988 | 0.0012 | -0.2808 |
| 8.7 | 903 | 0.25 | 1.0000 | 1 | 0.9987 | 0.0013 | -0.2371 |
| 8.9 | 1380 | 0.05 | 1.0000 | 1 | 0.9988 | 0.0012 | -0.2777 |
| 8.9 | 2205 | 0.05 | 1.0000 | 1 | 0.9985 | 0.0015 | -0.2298 |
| 9 | 1860 | 0.05 | 1.0000 | 1 | 0.9987 | 0.0013 | -0.2465 |
| 9 | 2569 | 0.15 | 1.0000 | 1 | 0.9982 | 0.0018 | -0.1679 |
| 9.1 | 988 | 0.1 | 1.0000 | 1 | 0.9987 | 0.0013 | -0.2751 |
| 9.1 | 8325 | 18.7 | 1.0000 | 2 | 0.0000 | 1.0000 | -0.3004 |
| 9.2 | 1301 | 31.75 | 1.0000 | 2 | 0.0000 | 1.0000 | -0.3321 |
| 9.2 | 1537 | 0.09 | 1.0000 | 1 | 0.9988 | 0.0012 | -0.2436 |
| 9.2 | 2642 | 9.1 | 1.0000 | 2 | 0.0000 | 1.0000 | -0.3319 |
| 9.2 | 2920 | 13.05 | 1.0000 | 2 | 0.0000 | 1.0000 | -0.3291 |
| 9.3 | 2627 | 1.26 | 1.0000 | 1 | 0.9980 | 0.0020 | 0.2616 |
| 9.3 | 5490 | 0.16 | 1.0000 | 1 | 0.7646 | 0.2354 | -0.0618 |
| 9.4 | 851 | 1.46 | 1.0000 | 1 | 0.9982 | 0.0018 | 0.2369 |
| 9.4 | 1525 | 7.15 | 1.0000 | 2 | 0.0002 | 0.9998 | -0.3314 |
| 9.6 | 704 | 3.2 | 1.0000 | 1 | 0.9814 | 0.0186 | 0.8671 |
| 9.6 | 1243 | 0.99 | 1.0000 | 1 | 0.9987 | 0.0013 | 0.0904 |
| 9.6 | 1381 | 2.14 | 1.0000 | 1 | 0.9971 | 0.0029 | 0.5285 |
| 9.6 | 17875 | 199.3 | 1.0000 | 2 | 0.0000 | 1.0000 | -0.1708 |
| 9.7 | 1073 | 0.13 | 1.0000 | 1 | 0.9987 | 0.0013 | -0.2388 |
| 9.9 | 1387 | 3.79 | 1.0000 | 1 | 0.9538 | 0.0462 | 1.0930 |
| 10.1 | 784 | 0.96 | 1.0000 | 1 | 0.9985 | 0.0015 | 0.0691 |
| 10.2 | 1141 | 0.06 | 1.0000 | 1 | 0.9986 | 0.0014 | -0.2443 |
| 10.3 | 295 | 0.06 | 1.0000 | 1 | 0.9978 | 0.0022 | -0.2901 |
| 10.3 | 331 | 0.15 | 1.0000 | 1 | 0.9979 | 0.0021 | -0.2543 |
| 10.3 | 699 | 0.2 | 1.0000 | 1 | 0.9984 | 0.0016 | -0.2142 |
| 10.3 | 2545 | 2.86 | 1.0000 | 1 | 0.9899 | 0.0101 | 0.8806 |
| 10.4 | 870 | 0.33 | 1.0000 | 1 | 0.9986 | 0.0014 | -0.1521 |
| 10.4 | 20000 | 0.53 | 1.0000 | 2 | 0.0000 | 1.0000 | -0.1956 |
| 10.5 | 848 | 0.05 | 1.0000 | 1 | 0.9984 | 0.0016 | -0.2551 |
| 10.5 | 1204 | 0.37 | 1.0000 | 1 | 0.9987 | 0.0013 | -0.1143 |
| 10.5 | 1230 | 0.05 | 1.0000 | 1 | 0.9986 | 0.0014 | -0.2328 |
| 10.5 | 2893 | 0.21 | 1.0000 | 1 | 0.9975 | 0.0025 | -0.0764 |
| 10.6 | 979 | 0.05 | 1.0000 | 1 | 0.9985 | 0.0015 | -0.2441 |
| 10.6 | 1929 | 0.75 | 1.0000 | 1 | 0.9987 | 0.0013 | 0.0737 |
| 10.6 | 3210 | 11.9 | 1.0000 | 2 | 0.0000 | 1.0000 | -0.2792 |
| 10.7 | 3122 | 20.35 | 1.0000 | 2 | 0.0000 | 1.0000 | -0.2735 |
| 10.8 | 417 | 0.05 | 1.0000 | 1 | 0.9979 | 0.0021 | -0.2701 |
| 10.8 | 3810 | 0.43 | 1.0000 | 1 | 0.9922 | 0.0078 | 0.0675 |
| 10.9 | 1280 | 0.18 | 1.0000 | 1 | 0.9986 | 0.0014 | -0.1678 |
| 10.9 | 2497 | 0.33 | 1.0000 | 1 | 0.9982 | 0.0018 | -0.0409 |
| 11 | 250 | 0.67 | 1.0000 | 1 | 0.9978 | 0.0022 | -0.0407 |
| 11.1 | 2532 | 10.14 | 1.0000 | 2 | 0.0000 | 1.0000 | -0.2662 |
| 11.1 | 3190 | 0.19 | 1.0000 | 1 | 0.9964 | 0.0036 | -0.0468 |
| 11.2 | 4482 | 0.07 | 1.0000 | 1 | 0.9696 | 0.0304 | -0.0199 |
| 11.3 | 803 | 0.05 | 1.0000 | 1 | 0.9983 | 0.0017 | -0.2309 |
| 11.4 | 912 | 0.05 | 1.0000 | 1 | 0.9984 | 0.0016 | -0.2212 |
| 11.4 | 2305 | 19.17 | 1.0000 | 2 | 0.0000 | 1.0000 | -0.2542 |
| 11.4 | 12480 | 0.09 | 1.0000 | 2 | 0.0000 | 1.0000 | -0.2032 |
| 11.5 | 1196 | 3.04 | 1.0000 | 1 | 0.9888 | 0.0112 | 0.9087 |
| 11.5 | 1319 | 0.05 | 1.0000 | 1 | 0.9985 | 0.0015 | -0.1942 |
| 11.6 | 4579 | 6.93 | 1.0000 | 2 | 0.0000 | 1.0000 | -0.2383 |
| 11.7 | 1003 | 2.63 | 1.0000 | 1 | 0.9937 | 0.0063 | 0.7570 |
| 11.7 | 1443 | 118.7 | 1.0000 | 2 | 0.0000 | 1.0000 | -0.2163 |
| 11.7 | 2912 | 0.29 | 1.0000 | 1 | 0.9973 | 0.0027 | -0.0052 |
| 11.8 | 2205 | 24.3 | 1.0000 | 2 | 0.0000 | 1.0000 | -0.2392 |
| 11.9 | 1130 | 0.05 | 1.0000 | 1 | 0.9984 | 0.0016 | -0.1918 |
| 11.9 | 3369 | 7.68 | 1.0000 | 2 | 0.0000 | 1.0000 | -0.2345 |
| 12 | 497 | 3.67 | 1.0000 | 1 | 0.9447 | 0.0553 | 1.0583 |
| 12 | 3504 | 39.56 | 1.0000 | 2 | 0.0000 | 1.0000 | -0.2200 |
| 12.1 | 1083 | 0.18 | 1.0000 | 1 | 0.9984 | 0.0016 | -0.1390 |
| 12.1 | 2047 | 0.24 | 1.0000 | 1 | 0.9984 | 0.0016 | -0.0605 |
| 12.2 | 4346 | 11.93 | 1.0000 | 2 | 0.0000 | 1.0000 | -0.2173 |
| 12.2 | 4732 | 0.07 | 1.0000 | 1 | 0.9457 | 0.0543 | 0.0217 |
| 12.3 | 1848 | 0.12 | 1.0000 | 1 | 0.9984 | 0.0016 | -0.1104 |
| 12.5 | 755 | 0.15 | 1.0000 | 1 | 0.9981 | 0.0019 | -0.1559 |
| 12.5 | 759 | 0.51 | 1.0000 | 1 | 0.9983 | 0.0017 | -0.0207 |
| 12.5 | 1340 | 0.05 | 1.0000 | 1 | 0.9984 | 0.0016 | -0.1594 |
| 12.5 | 2237 | 0.09 | 1.0000 | 1 | 0.9981 | 0.0019 | -0.0924 |
| 12.5 | 3416 | 0.05 | 1.0000 | 1 | 0.9944 | 0.0056 | -0.0395 |
| 12.5 | 7430 | 9.47 | 1.0000 | 2 | 0.0000 | 1.0000 | -0.1904 |
| 12.6 | 31 | 0.66 | 1.0000 | 1 | 0.9969 | 0.0031 | -0.0037 |
| 12.6 | 851 | 0.68 | 1.0000 | 1 | 0.9983 | 0.0017 | 0.0518 |
| 12.7 | 1701 | 0.54 | 1.0000 | 1 | 0.9986 | 0.0014 | 0.0521 |
| 12.8 | 1053 | 1.17 | 1.0000 | 1 | 0.9983 | 0.0017 | 0.2539 |
| 12.8 | 3730 | 0.44 | 1.0000 | 1 | 0.9922 | 0.0078 | 0.1336 |
| 12.9 | 190 | 0.55 | 1.0000 | 1 | 0.9973 | 0.0027 | -0.0255 |
| 12.9 | 1816 | 0.09 | 1.0000 | 1 | 0.9983 | 0.0017 | -0.1034 |
| 13 | 879 | 0.05 | 1.0000 | 1 | 0.9981 | 0.0019 | -0.1695 |
| 13 | 1229 | 0.3 | 1.0000 | 1 | 0.9984 | 0.0016 | -0.0554 |
| 13.1 | 1088 | 0.15 | 1.0000 | 1 | 0.9983 | 0.0017 | -0.1165 |
| 13.1 | 1246 | 0.38 | 1.0000 | 1 | 0.9985 | 0.0015 | -0.0210 |
| 13.1 | 1600 | 0.28 | 1.0000 | 1 | 0.9985 | 0.0015 | -0.0380 |
| 13.1 | 2070 | 0.12 | 1.0000 | 1 | 0.9982 | 0.0018 | -0.0707 |
| 13.2 | 788 | 25.91 | 1.0000 | 2 | 0.0000 | 1.0000 | -0.1980 |
| 13.2 | 3843 | 0.33 | 1.0000 | 1 | 0.9901 | 0.0099 | 0.1119 |
| 13.3 | 2833 | 0.05 | 1.0000 | 1 | 0.9969 | 0.0031 | -0.0461 |
| 13.4 | 501 | 0.09 | 1.0000 | 1 | 0.9976 | 0.0024 | -0.1631 |
| 13.6 | 1142 | 0.06 | 1.0000 | 1 | 0.9982 | 0.0018 | -0.1303 |
| 13.6 | 1350 | 0.86 | 1.0000 | 1 | 0.9985 | 0.0015 | 0.1818 |
| 13.6 | 1980 | 0.13 | 1.0000 | 1 | 0.9982 | 0.0018 | -0.0554 |
| 13.8 | 427 | 0.2 | 1.0000 | 1 | 0.9975 | 0.0025 | -0.1127 |
| 13.8 | 4830 | 0.12 | 1.0000 | 1 | 0.9275 | 0.0725 | 0.0935 |
| 14.1 | 4915 | 7.57 | 1.0000 | 2 | 0.0000 | 1.0000 | -0.1495 |
| 14.2 | 1216 | 11.06 | 1.0000 | 2 | 0.0000 | 1.0000 | -0.1657 |
| 14.2 | 2675 | 0.68 | 1.0000 | 1 | 0.9975 | 0.0025 | 0.2111 |
| 14.3 | 1721 | 1.47 | 1.0000 | 1 | 0.9980 | 0.0020 | 0.4554 |
| 14.4 | 974 | 0.2 | 1.0000 | 1 | 0.9981 | 0.0019 | -0.0608 |
| 14.4 | 1421 | 0.19 | 1.0000 | 1 | 0.9982 | 0.0018 | -0.0386 |
| 14.5 | 808 | 0.14 | 1.0000 | 1 | 0.9979 | 0.0021 | -0.0896 |
| 14.5 | 1944 | 0.81 | 1.0000 | 1 | 0.9983 | 0.0017 | 0.2277 |
| 14.6 | 1034 | 0.05 | 1.0000 | 1 | 0.9980 | 0.0020 | -0.1069 |
| 14.6 | 3350 | 0.51 | 1.0000 | 1 | 0.9949 | 0.0051 | 0.1990 |
| 14.7 | 270 | 0.98 | 1.0000 | 1 | 0.9970 | 0.0030 | 0.2004 |
| 14.7 | 844 | 80 | 1.0000 | 2 | 0.0000 | 1.0000 | -0.1281 |
| 14.7 | 1484 | 1.52 | 1.0000 | 1 | 0.9979 | 0.0021 | 0.4737 |
| 14.7 | 2464 | 0.51 | 1.0000 | 1 | 0.9978 | 0.0022 | 0.1519 |
| 14.8 | 271 | 26.81 | 1.0000 | 2 | 0.0000 | 1.0000 | -0.1451 |
| 14.9 | 337 | 0.33 | 1.0000 | 1 | 0.9972 | 0.0028 | -0.0324 |
| 14.9 | 1039 | 0.82 | 1.0000 | 1 | 0.9982 | 0.0018 | 0.1922 |
| 14.9 | 2114 | 0.29 | 1.0000 | 1 | 0.9980 | 0.0020 | 0.0559 |
| 15 | 1550 | 0.96 | 1.0000 | 1 | 0.9983 | 0.0017 | 0.2778 |
| 15.1 | 751 | 16.41 | 1.0000 | 2 | 0.0000 | 1.0000 | -0.1354 |
| 15.1 | 1814 | 0.14 | 1.0000 | 1 | 0.9981 | 0.0019 | -0.0111 |
| 15.1 | 2117 | 0.43 | 1.0000 | 1 | 0.9981 | 0.0019 | 0.1153 |
| 15.2 | 1106 | 1.87 | 1.0000 | 1 | 0.9971 | 0.0029 | 0.5991 |
| 15.2 | 1256 | 0.17 | 1.0000 | 1 | 0.9981 | 0.0019 | -0.0289 |
| 15.3 | 779 | 3.53 | 1.0000 | 1 | 0.9647 | 0.0353 | 1.1617 |
| 15.3 | 1975 | 12.61 | 1.0000 | 2 | 0.0000 | 1.0000 | -0.1228 |
| 15.3 | 2560 | 1.58 | 1.0000 | 1 | 0.9970 | 0.0030 | 0.5781 |
| 15.3 | 2709 | 0.28 | 1.0000 | 1 | 0.9970 | 0.0030 | 0.0999 |
| 15.5 | 2013 | 2.53 | 1.0000 | 1 | 0.9944 | 0.0056 | 0.9062 |
| 15.5 | 2054 | 0.31 | 1.0000 | 1 | 0.9980 | 0.0020 | 0.0800 |
| 15.6 | 3606 | 0.25 | 1.0000 | 1 | 0.9916 | 0.0084 | 0.1493 |
| 15.7 | 494 | 0.28 | 1.0000 | 1 | 0.9973 | 0.0027 | -0.0152 |
| 15.7 | 944 | 0.66 | 1.0000 | 1 | 0.9980 | 0.0020 | 0.1535 |
| 15.7 | 1283 | 0.05 | 1.0000 | 1 | 0.9979 | 0.0021 | -0.0555 |
| 15.7 | 1650 | 18.61 | 1.0000 | 2 | 0.0000 | 1.0000 | -0.1088 |
| 15.9 | 2911 | 2.25 | 1.0000 | 1 | 0.9931 | 0.0069 | 0.8657 |
| 15.9 | 3338 | 0.33 | 1.0000 | 1 | 0.9942 | 0.0058 | 0.1744 |
| 16 | 1623 | 0.13 | 1.0000 | 1 | 0.9979 | 0.0021 | 0.0043 |
| 16.1 | 514 | 0.41 | 1.0000 | 1 | 0.9973 | 0.0027 | 0.0481 |
| 16.1 | 2078 | 0.32 | 1.0000 | 1 | 0.9979 | 0.0021 | 0.1052 |
| 16.1 | 9310 | 5.39 | 1.0000 | 2 | 0.0000 | 1.0000 | -0.0562 |
| 16.2 | 2905 | 200 | 1.0000 | 2 | 0.0000 | 1.0000 | -0.0256 |
| 16.3 | 426 | 6.68 | 1.0000 | 2 | 0.0011 | 0.9989 | -0.0959 |
| 16.4 | 491 | 0.16 | 1.0000 | 1 | 0.9970 | 0.0030 | -0.0369 |
| 16.4 | 1379 | 0.08 | 1.0000 | 1 | 0.9978 | 0.0022 | -0.0153 |
| 16.4 | 33440 | 0.21 | 1.0000 | 2 | 0.0000 | 1.0000 | 0.0881 |
| 16.6 | 1194 | 7.39 | 1.0000 | 2 | 0.0001 | 0.9999 | -0.0835 |
| 16.6 | 4595 | 14.86 | 1.0000 | 2 | 0.0000 | 1.0000 | -0.0622 |
| 16.7 | 1308 | 0.05 | 1.0000 | 1 | 0.9977 | 0.0023 | -0.0206 |
| 16.7 | 1382 | 44.14 | 1.0000 | 2 | 0.0000 | 1.0000 | -0.0673 |
| 16.7 | 1702 | 0.06 | 1.0000 | 1 | 0.9977 | 0.0023 | 0.0060 |
| 16.7 | 4326 | 10.36 | 1.0000 | 2 | 0.0000 | 1.0000 | -0.0617 |
| 16.8 | 1623 | 0.05 | 1.0000 | 1 | 0.9977 | 0.0023 | 0.0011 |
| 16.8 | 2284 | 20.1 | 1.0000 | 2 | 0.0000 | 1.0000 | -0.0665 |
| 16.8 | 3000 | 0.4 | 1.0000 | 1 | 0.9958 | 0.0042 | 0.2117 |
| 16.9 | 444 | 0.56 | 1.0000 | 1 | 0.9970 | 0.0030 | 0.1270 |
| 17.1 | 702 | 0.19 | 1.0000 | 1 | 0.9972 | 0.0028 | 0.0101 |
| 17.1 | 2988 | 15.09 | 1.0000 | 2 | 0.0000 | 1.0000 | -0.0538 |
| 17.2 | 1028 | 0.23 | 1.0000 | 1 | 0.9976 | 0.0024 | 0.0474 |
| 17.2 | 1739 | 0.79 | 1.0000 | 1 | 0.9980 | 0.0020 | 0.2987 |
| 17.2 | 2918 | 0.08 | 1.0000 | 1 | 0.9954 | 0.0046 | 0.1005 |
| 17.2 | 2997 | 0.25 | 1.0000 | 1 | 0.9954 | 0.0046 | 0.1687 |
| 17.3 | 1427 | 0.25 | 1.0000 | 1 | 0.9978 | 0.0022 | 0.0814 |
| 17.4 | 1835 | 0.58 | 1.0000 | 1 | 0.9979 | 0.0021 | 0.2322 |
| 17.4 | 4236 | 0.5 | 1.0000 | 1 | 0.9753 | 0.0247 | 0.3329 |
| 17.7 | 603 | 0.42 | 1.0000 | 1 | 0.9971 | 0.0029 | 0.1106 |
| 17.7 | 1141 | 1.99 | 1.0000 | 1 | 0.9963 | 0.0037 | 0.7293 |
| 17.7 | 4812 | 3.35 | 1.0000 | 1 | 0.6312 | 0.3688 | 0.9096 |
| 17.8 | 2050 | 0.49 | 1.0000 | 1 | 0.9977 | 0.0023 | 0.2243 |
| 18 | 327 | 0.26 | 1.0000 | 1 | 0.9961 | 0.0039 | 0.0446 |
| 18 | 2055 | 60.68 | 1.0000 | 2 | 0.0000 | 1.0000 | -0.0130 |
| 18.1 | 1697 | 51.24 | 1.0000 | 2 | 0.0000 | 1.0000 | -0.0146 |
| 18.3 | 4573 | 0.69 | 1.0000 | 1 | 0.9521 | 0.0479 | 0.4410 |
| 18.3 | 6800 | 19.56 | 1.0000 | 2 | 0.0000 | 1.0000 | 0.0107 |
| 18.4 | 1136 | 1.45 | 1.0000 | 1 | 0.9973 | 0.0027 | 0.5509 |
| 18.4 | 2636 | 0.29 | 1.0000 | 1 | 0.9964 | 0.0036 | 0.2032 |
| 18.5 | 1764 | 17.23 | 1.0000 | 2 | 0.0000 | 1.0000 | -0.0114 |
| 18.6 | 311 | 1.89 | 1.0000 | 1 | 0.9941 | 0.0059 | 0.6723 |
| 18.6 | 2269 | 0.23 | 1.0000 | 1 | 0.9970 | 0.0030 | 0.1662 |
| 18.7 | 2334 | 11.71 | 1.0000 | 2 | 0.0000 | 1.0000 | -0.0030 |
| 18.7 | 2606 | 4.69 | 1.0000 | 1 | 0.6492 | 0.3508 | 1.2096 |
| 18.8 | 1547 | 5.6 | 1.0000 | 2 | 0.1257 | 0.8743 | 0.2649 |
| 19 | 1171 | 0.42 | 1.0000 | 1 | 0.9975 | 0.0025 | 0.1872 |
| 19.2 | 996 | 0.05 | 1.0000 | 1 | 0.9968 | 0.0032 | 0.0451 |
| 19.2 | 1007 | 5.76 | 1.0000 | 2 | 0.0637 | 0.9363 | 0.1441 |
| 19.3 | 20000 | 28.92 | 1.0000 | 2 | 0.0000 | 1.0000 | 0.1226 |
| 19.4 | 3517 | 11.43 | 1.0000 | 2 | 0.0000 | 1.0000 | 0.0278 |
| 19.4 | 5139 | 21.97 | 1.0000 | 2 | 0.0000 | 1.0000 | 0.0403 |
| 19.6 | 2936 | 20.09 | 1.0000 | 2 | 0.0000 | 1.0000 | 0.0343 |
| 19.7 | 1779 | 0.59 | 1.0000 | 1 | 0.9975 | 0.0025 | 0.3096 |
| 19.8 | 964 | 0.3 | 1.0000 | 1 | 0.9970 | 0.0030 | 0.1569 |
| 19.8 | 1400 | 0.47 | 1.0000 | 1 | 0.9974 | 0.0026 | 0.2460 |
| 19.8 | 1504 | 3.49 | 1.0000 | 1 | 0.9726 | 0.0274 | 1.3499 |
| 19.8 | 3632 | 0.24 | 1.0000 | 1 | 0.9875 | 0.0125 | 0.2869 |
| 19.8 | 5120 | 3.68 | 1.0000 | 2 | 0.3162 | 0.6838 | 0.5606 |
| 19.9 | 1170 | 3.1 | 1.0000 | 1 | 0.9846 | 0.0154 | 1.2061 |
| 20 | 829 | 5.63 | 1.0000 | 2 | 0.0879 | 0.9121 | 0.2187 |
| 20 | 3707 | 31.48 | 1.0000 | 2 | 0.0000 | 1.0000 | 0.0562 |
| 20.1 | 933 | 7.74 | 1.0000 | 2 | 0.0000 | 1.0000 | 0.0365 |
| 20.3 | 3138 | 0.23 | 1.0000 | 1 | 0.9930 | 0.0070 | 0.2727 |
| 20.4 | 1193 | 0.1 | 1.0000 | 1 | 0.9967 | 0.0033 | 0.1154 |
| 20.4 | 3670 | 0.05 | 1.0000 | 1 | 0.9844 | 0.0156 | 0.2382 |
| 20.4 | 6500 | 5.2 | 1.0000 | 2 | 0.0003 | 0.9997 | 0.0779 |
| 20.7 | 1114 | 5.24 | 1.0000 | 2 | 0.2893 | 0.7107 | 0.6345 |
| 20.8 | 700 | 0.23 | 1.0000 | 1 | 0.9960 | 0.0040 | 0.1488 |
| 20.8 | 1504 | 0.28 | 1.0000 | 1 | 0.9970 | 0.0030 | 0.2143 |
| 20.8 | 2142 | 6.52 | 1.0000 | 2 | 0.0052 | 0.9948 | 0.0803 |
| 21 | 2990 | 0.51 | 1.0000 | 1 | 0.9943 | 0.0057 | 0.3925 |
| 21.2 | 1014 | 2.82 | 1.0000 | 1 | 0.9885 | 0.0115 | 1.1413 |
| 21.2 | 2180 | 12.81 | 1.0000 | 2 | 0.0000 | 1.0000 | 0.0832 |
| 21.2 | 3173 | 53.78 | 1.0000 | 2 | 0.0000 | 1.0000 | 0.1021 |
| 21.3 | 4440 | 75.54 | 1.0000 | 2 | 0.0000 | 1.0000 | 0.1197 |
| 21.4 | 984 | 0.07 | 1.0000 | 1 | 0.9960 | 0.0040 | 0.1255 |
| 21.5 | 396 | 0.11 | 1.0000 | 1 | 0.9944 | 0.0056 | 0.1097 |
| 21.5 | 1231 | 0.32 | 1.0000 | 1 | 0.9967 | 0.0033 | 0.2369 |
| 21.6 | 737 | 0.11 | 1.0000 | 1 | 0.9955 | 0.0045 | 0.1329 |
| 21.6 | 1160 | 0.5 | 1.0000 | 1 | 0.9968 | 0.0032 | 0.3035 |
| 21.6 | 6910 | 21.97 | 1.0000 | 2 | 0.0000 | 1.0000 | 0.1267 |
| 21.7 | 1091 | 21.75 | 1.0000 | 2 | 0.0000 | 1.0000 | 0.0974 |
| 21.7 | 2368 | 0.22 | 1.0000 | 1 | 0.9957 | 0.0043 | 0.2719 |
| 21.8 | 8350 | 106.4 | 1.0000 | 2 | 0.0000 | 1.0000 | 0.1691 |
| 21.9 | 1562 | 12.36 | 1.0000 | 2 | 0.0000 | 1.0000 | 0.1039 |
| 22 | 3406 | 0.62 | 1.0000 | 1 | 0.9903 | 0.0097 | 0.4897 |
| 22.1 | 1227 | 4.49 | 1.0000 | 1 | 0.7777 | 0.2223 | 1.4419 |
| 22.1 | 3890 | 12.87 | 1.0000 | 2 | 0.0000 | 1.0000 | 0.1241 |
| 22.3 | 1104 | 1.02 | 1.0000 | 1 | 0.9967 | 0.0033 | 0.5184 |
| 22.4 | 605 | 0.25 | 1.0000 | 1 | 0.9951 | 0.0049 | 0.2044 |
| 22.4 | 3222 | 9.08 | 1.0000 | 2 | 0.0000 | 1.0000 | 0.1295 |
| 22.5 | 581 | 0.57 | 1.0000 | 1 | 0.9955 | 0.0045 | 0.3260 |
| 22.6 | 1394 | 3.03 | 1.0000 | 1 | 0.9854 | 0.0146 | 1.2846 |
| 22.6 | 3356 | 0.73 | 1.0000 | 1 | 0.9907 | 0.0093 | 0.5480 |
| 22.9 | 1229 | 0.99 | 1.0000 | 1 | 0.9967 | 0.0033 | 0.5345 |
| 22.9 | 2419 | 0.22 | 1.0000 | 1 | 0.9950 | 0.0050 | 0.3150 |
| 22.9 | 2909 | 3.59 | 1.0000 | 1 | 0.9441 | 0.0559 | 1.5282 |
| 22.9 | 10000 | 20 | 1.0000 | 2 | 0.0000 | 1.0000 | 0.1885 |
| 23 | 1653 | 0.96 | 1.0000 | 1 | 0.9968 | 0.0032 | 0.5513 |
| 23.1 | 993 | 1.22 | 1.0000 | 1 | 0.9962 | 0.0038 | 0.6134 |
| 23.1 | 1475 | 8.37 | 1.0000 | 2 | 0.0000 | 1.0000 | 0.1438 |
| 23.1 | 1955 | 79.35 | 1.0000 | 2 | 0.0000 | 1.0000 | 0.1695 |
| 23.2 | 4092 | 7.09 | 1.0000 | 2 | 0.0001 | 0.9999 | 0.1619 |
| 23.3 | 488 | 26.99 | 1.0000 | 2 | 0.0000 | 1.0000 | 0.1512 |
| 23.3 | 2135 | 0.86 | 1.0000 | 1 | 0.9963 | 0.0037 | 0.5517 |
| 23.4 | 1176 | 0.6 | 1.0000 | 1 | 0.9963 | 0.0037 | 0.4021 |
| 23.4 | 1312 | 120.4 | 1.0000 | 2 | 0.0000 | 1.0000 | 0.1896 |
| 23.4 | 1802 | 0.44 | 1.0000 | 1 | 0.9962 | 0.0038 | 0.3785 |
| 23.4 | 4819 | 0.35 | 1.0000 | 1 | 0.8690 | 0.1310 | 0.4751 |
| 23.5 | 294 | 0.08 | 1.0000 | 1 | 0.9923 | 0.0077 | 0.1596 |
| 23.5 | 3427 | 0.36 | 1.0000 | 1 | 0.9873 | 0.0127 | 0.4436 |
| 23.6 | 966 | 29.77 | 1.0000 | 2 | 0.0000 | 1.0000 | 0.1652 |
| 23.6 | 2680 | 0.59 | 1.0000 | 1 | 0.9946 | 0.0054 | 0.4917 |
| 23.7 | 1230 | 0.51 | 1.0000 | 1 | 0.9961 | 0.0039 | 0.3816 |
| 23.8 | 1137 | 1.18 | 1.0000 | 1 | 0.9962 | 0.0038 | 0.6303 |
| 24 | 1698 | 53.11 | 1.0000 | 2 | 0.0000 | 1.0000 | 0.1908 |
| 24 | 5560 | 0.17 | 1.0000 | 2 | 0.4634 | 0.5366 | 0.3443 |
| 24.1 | 2573 | 5.87 | 1.0000 | 2 | 0.0511 | 0.9489 | 0.3015 |
| 24.1 | 2605 | 0.26 | 1.0000 | 1 | 0.9938 | 0.0062 | 0.3807 |
| 24.4 | 790 | 200 | 1.0000 | 2 | 0.0000 | 1.0000 | 0.2472 |
| 24.4 | 3748 | 4.54 | 1.0000 | 2 | 0.4679 | 0.5321 | 1.0735 |
| 24.5 | 826 | 2.71 | 1.0000 | 1 | 0.9868 | 0.0132 | 1.1986 |
| 24.6 | 4634 | 27.65 | 1.0000 | 2 | 0.0000 | 1.0000 | 0.2199 |
| 24.6 | 10000 | 200 | 1.0000 | 2 | 0.0000 | 1.0000 | 0.3059 |
| 24.9 | 2700 | 42.34 | 1.0000 | 2 | 0.0000 | 1.0000 | 0.2242 |
| 24.9 | 3922 | 0.09 | 1.0000 | 1 | 0.9650 | 0.0350 | 0.4143 |
| 24.9 | 4316 | 11.03 | 1.0000 | 2 | 0.0000 | 1.0000 | 0.2231 |
| 24.9 | 6570 | 3.69 | 1.0000 | 2 | 0.0066 | 0.9934 | 0.2446 |
| 24.9 | 9510 | 0.32 | 1.0000 | 2 | 0.0000 | 1.0000 | 0.2488 |
| 25 | 708 | 20.12 | 1.0000 | 2 | 0.0000 | 1.0000 | 0.2093 |
| 25 | 934 | 25.01 | 1.0000 | 2 | 0.0000 | 1.0000 | 0.2121 |
| 25 | 1830 | 0.31 | 1.0000 | 1 | 0.9953 | 0.0047 | 0.3849 |
| 25 | 6110 | 18.08 | 1.0000 | 2 | 0.0000 | 1.0000 | 0.2389 |
| 25.1 | 2619 | 10.24 | 1.0000 | 2 | 0.0000 | 1.0000 | 0.2203 |
| 25.2 | 1364 | 13.71 | 1.0000 | 2 | 0.0000 | 1.0000 | 0.2178 |
| 25.2 | 3468 | 1.23 | 1.0000 | 1 | 0.9870 | 0.0130 | 0.8255 |
| 25.3 | 1717 | 0.88 | 1.0000 | 1 | 0.9960 | 0.0040 | 0.6018 |
| 25.4 | 2320 | 20.79 | 1.0000 | 2 | 0.0000 | 1.0000 | 0.2324 |
| 25.5 | 4420 | 0.45 | 1.0000 | 1 | 0.9297 | 0.0703 | 0.5802 |
| 25.6 | 1027 | 8.72 | 1.0000 | 2 | 0.0000 | 1.0000 | 0.2282 |
| 25.9 | 5950 | 1.75 | 1.0000 | 2 | 0.2460 | 0.7540 | 0.4934 |
| 26.1 | 47 | 0.18 | 1.0000 | 1 | 0.9875 | 0.0125 | 0.2696 |
| 26.1 | 2883 | 25.47 | 1.0000 | 2 | 0.0000 | 1.0000 | 0.2614 |
| 26.3 | 731 | 0.98 | 1.0000 | 1 | 0.9942 | 0.0058 | 0.6149 |
| 26.6 | 1076 | 0.98 | 1.0000 | 1 | 0.9950 | 0.0050 | 0.6453 |
| 26.6 | 3115 | 35.42 | 1.0000 | 2 | 0.0000 | 1.0000 | 0.2833 |
| 26.7 | 1167 | 34.45 | 1.0000 | 2 | 0.0000 | 1.0000 | 0.2755 |
| 26.8 | 647 | 7.56 | 1.0000 | 2 | 0.0000 | 1.0000 | 0.2674 |
| 27 | 793 | 1.13 | 1.0000 | 1 | 0.9940 | 0.0060 | 0.6979 |
| 27 | 3225 | 21.09 | 1.0000 | 2 | 0.0000 | 1.0000 | 0.2931 |
| 27.1 | 1120 | 0.85 | 1.0000 | 1 | 0.9947 | 0.0053 | 0.6159 |
| 27.1 | 1800 | 0.13 | 1.0000 | 1 | 0.9933 | 0.0067 | 0.3861 |
| 27.5 | 1487 | 2.97 | 1.0000 | 1 | 0.9828 | 0.0172 | 1.4290 |
| 27.5 | 2982 | 200 | 1.0000 | 2 | 0.0000 | 1.0000 | 0.3671 |
| 27.6 | 5270 | 20.52 | 1.0000 | 2 | 0.0000 | 1.0000 | 0.3253 |
| 27.8 | 3328 | 70.15 | 1.0000 | 2 | 0.0000 | 1.0000 | 0.3374 |
| 28 | 1593 | 0.31 | 1.0000 | 1 | 0.9935 | 0.0065 | 0.4715 |
| 28.2 | 2783 | 0.28 | 1.0000 | 1 | 0.9888 | 0.0112 | 0.5348 |
| 28.3 | 176 | 0.81 | 1.0000 | 1 | 0.9887 | 0.0113 | 0.5848 |
| 28.3 | 2033 | 0.2 | 1.0000 | 1 | 0.9922 | 0.0078 | 0.4657 |
| 28.4 | 2644 | 34.35 | 1.0000 | 2 | 0.0000 | 1.0000 | 0.3428 |
| 28.4 | 4633 | 61.35 | 1.0000 | 2 | 0.0000 | 1.0000 | 0.3627 |
| 28.5 | 817 | 0.55 | 1.0000 | 1 | 0.9924 | 0.0076 | 0.5327 |
| 28.5 | 1252 | 53.31 | 1.0000 | 2 | 0.0000 | 1.0000 | 0.3446 |
| 28.6 | 1244 | 1.79 | 1.0000 | 1 | 0.9931 | 0.0069 | 1.0235 |
| 28.6 | 1422 | 0.24 | 1.0000 | 1 | 0.9926 | 0.0074 | 0.4554 |
| 28.7 | 397 | 0.27 | 1.0000 | 1 | 0.9882 | 0.0118 | 0.4104 |
| 28.8 | 7230 | 12.2 | 1.0000 | 2 | 0.0000 | 1.0000 | 0.3752 |
| 28.9 | 3242 | 5.15 | 1.0000 | 2 | 0.2336 | 0.7664 | 0.8358 |
| 29 | 1587 | 1.72 | 1.0000 | 1 | 0.9935 | 0.0065 | 1.0309 |
| 29.1 | 2875 | 1.52 | 1.0000 | 1 | 0.9897 | 0.0103 | 1.0315 |
| 29.3 | 1887 | 1.01 | 1.0000 | 1 | 0.9938 | 0.0062 | 0.7934 |
| 29.5 | 1591 | 0.79 | 1.0000 | 1 | 0.9936 | 0.0064 | 0.7008 |
| 29.5 | 2185 | 49.36 | 1.0000 | 2 | 0.0000 | 1.0000 | 0.3832 |
| 29.5 | 2609 | 2.86 | 1.0000 | 1 | 0.9781 | 0.0219 | 1.5143 |
| 29.5 | 4502 | 71.2 | 1.0000 | 2 | 0.0000 | 1.0000 | 0.4033 |
| 29.7 | 986 | 4.14 | 1.0000 | 1 | 0.8485 | 0.1515 | 1.6959 |
| 29.9 | 249 | 0.69 | 1.0000 | 1 | 0.9869 | 0.0131 | 0.5977 |
| 29.9 | 964 | 32.05 | 1.0000 | 2 | 0.0000 | 1.0000 | 0.3847 |
| 29.9 | 1616 | 0.18 | 1.0000 | 1 | 0.9910 | 0.0090 | 0.4877 |
| 30 | 1384 | 31.73 | 1.0000 | 2 | 0.0000 | 1.0000 | 0.3904 |
| 30 | 4688 | 0.93 | 1.0000 | 1 | 0.8538 | 0.1462 | 0.8716 |
| 30.2 | 906 | 0.94 | 1.0000 | 1 | 0.9920 | 0.0080 | 0.7400 |
| 30.2 | 1712 | 1.68 | 1.0000 | 1 | 0.9928 | 0.0072 | 1.0629 |
| 30.5 | 762 | 1.82 | 1.0000 | 1 | 0.9896 | 0.0104 | 1.0681 |
| 30.7 | 2468 | 5.34 | 1.0000 | 2 | 0.2277 | 0.7723 | 0.8885 |
| 30.7 | 5020 | 24.37 | 1.0000 | 2 | 0.0000 | 1.0000 | 0.4327 |
| 30.8 | 1121 | 5.31 | 1.0000 | 2 | 0.2315 | 0.7685 | 0.8731 |
| 31 | 1569 | 23.95 | 1.0000 | 2 | 0.0000 | 1.0000 | 0.4236 |
| 31.1 | 2284 | 2.66 | 1.0000 | 1 | 0.9831 | 0.0169 | 1.4812 |
| 31.1 | 2765 | 98 | 1.0000 | 2 | 0.0000 | 1.0000 | 0.4578 |
| 31.4 | 4852 | 38.8 | 1.0000 | 2 | 0.0000 | 1.0000 | 0.4607 |
| 31.6 | 1687 | 29.18 | 1.0000 | 2 | 0.0000 | 1.0000 | 0.4468 |
| 31.9 | 1386 | 18.68 | 1.0000 | 2 | 0.0000 | 1.0000 | 0.4521 |
| 32.3 | 13230 | 8.93 | 1.0000 | 2 | 0.0000 | 1.0000 | 0.5294 |
| 32.5 | 1180 | 12.95 | 1.0000 | 2 | 0.0000 | 1.0000 | 0.4699 |
| 32.5 | 3037 | 0.47 | 1.0000 | 1 | 0.9790 | 0.0210 | 0.7613 |
| 32.5 | 4058 | 10.14 | 1.0000 | 2 | 0.0000 | 1.0000 | 0.4852 |
| 32.5 | 5880 | 56.79 | 1.0000 | 2 | 0.0000 | 1.0000 | 0.5105 |
| 32.8 | 2450 | 1.92 | 1.0000 | 1 | 0.9875 | 0.0125 | 1.2780 |
| 33.5 | 5000 | 0.37 | 1.0000 | 1 | 0.5914 | 0.4086 | 0.7323 |
| 33.8 | 4902 | 11.59 | 1.0000 | 2 | 0.0000 | 1.0000 | 0.5355 |
| 33.9 | 5082 | 0.14 | 1.0000 | 2 | 0.4816 | 0.5184 | 0.6678 |
| 34 | 1704 | 0.14 | 1.0000 | 1 | 0.9839 | 0.0161 | 0.6146 |
| 34.1 | 3498 | 6.58 | 1.0000 | 2 | 0.0023 | 0.9977 | 0.5423 |
| 34.3 | 1927 | 0.08 | 1.0000 | 1 | 0.9817 | 0.0183 | 0.6151 |
| 34.6 | 1788 | 12.27 | 1.0000 | 2 | 0.0000 | 1.0000 | 0.5460 |
| 34.7 | 4270 | 7.14 | 1.0000 | 2 | 0.0001 | 0.9999 | 0.5620 |
| 34.9 | 1206 | 1.45 | 1.0000 | 1 | 0.9879 | 0.0121 | 1.1027 |
| 35.7 | 830 | 7.25 | 1.0000 | 2 | 0.0002 | 0.9998 | 0.5778 |
| 36 | 729 | 2.63 | 1.0000 | 1 | 0.9683 | 0.0317 | 1.5309 |
| 36.3 | 3264 | 0.64 | 1.0000 | 1 | 0.9611 | 0.0389 | 0.9578 |
| 36.9 | 1046 | 5.26 | 1.0000 | 2 | 0.2134 | 0.7866 | 1.0417 |
| 37 | 1887 | 35.08 | 1.0000 | 2 | 0.0000 | 1.0000 | 0.6373 |
| 37.8 | 1507 | 4.5 | 1.0000 | 1 | 0.6726 | 0.3274 | 1.8078 |
| 37.9 | 2830 | 0.85 | 1.0000 | 1 | 0.9712 | 0.0288 | 1.0671 |
| 38 | 546 | 5.26 | 1.0000 | 2 | 0.1500 | 0.8500 | 0.9475 |
| 39 | 1836 | 12.52 | 1.0000 | 2 | 0.0000 | 1.0000 | 0.6991 |
| 39 | 2409 | 2.64 | 1.0000 | 1 | 0.9652 | 0.0348 | 1.7268 |
| 39.1 | 1807 | 55.61 | 1.0000 | 2 | 0.0000 | 1.0000 | 0.7164 |
| 39.3 | 1805 | 0.84 | 1.0000 | 1 | 0.9779 | 0.0221 | 1.0547 |
| 39.7 | 1274 | 1.78 | 1.0000 | 1 | 0.9776 | 0.0224 | 1.3830 |
| 40.1 | 1092 | 28.06 | 1.0000 | 2 | 0.0000 | 1.0000 | 0.7381 |
| 40.5 | 1813 | 25.54 | 1.0000 | 2 | 0.0000 | 1.0000 | 0.7553 |
| 40.5 | 7810 | 7.01 | 1.0000 | 2 | 0.0000 | 1.0000 | 0.7829 |
| 40.6 | 2821 | 2.06 | 1.0000 | 1 | 0.9622 | 0.0378 | 1.5908 |
| 40.8 | 737 | 1.52 | 1.0000 | 1 | 0.9680 | 0.0320 | 1.2884 |
| 41.4 | 799 | 1.05 | 1.0000 | 1 | 0.9646 | 0.0354 | 1.1399 |
| 41.6 | 5127 | 117.9 | 1.0000 | 2 | 0.0000 | 1.0000 | 0.8420 |
| 41.7 | 3725 | 77.96 | 1.0000 | 2 | 0.0000 | 1.0000 | 0.8246 |
| 42.5 | 2381 | 1.77 | 1.0000 | 1 | 0.9657 | 0.0343 | 1.5276 |
| 43.9 | 1771 | 0.76 | 1.0000 | 1 | 0.9551 | 0.0449 | 1.1704 |
| 44.6 | 2328 | 3.68 | 1.0000 | 1 | 0.8358 | 0.1642 | 2.1004 |
| 44.7 | 1160 | 0.26 | 1.0000 | 1 | 0.9209 | 0.0791 | 0.9804 |
| 45.1 | 1142 | 5.71 | 1.0000 | 2 | 0.0500 | 0.9500 | 1.0119 |
| 48.4 | 1266 | 1.47 | 1.0000 | 1 | 0.9274 | 0.0726 | 1.5327 |
| 51.8 | 8253 | 24.35 | 1.0000 | 2 | 0.0000 | 1.0000 | 1.1833 |
